# Hydantoin-bridged medium ring scaffolds by migratory insertion of urea-tethered nitrile anions into aromatic C–N bonds†

**DOI:** 10.1039/d0sc06188c

**Published:** 2020-12-14

**Authors:** Makenzie J. Millward, Emily Ellis, John W. Ward, Jonathan Clayden

**Affiliations:** School of Chemistry, University of Bristol Cantock's Close Bristol BS8 1TS UK j.clayden@bristol.ac.uk

## Abstract

Bicyclic or tricyclic nitrogen-containing heterocyclic scaffolds were constructed rapidly by intramolecular nucleophilic aromatic substitution of metallated nitriles tethered by a urea linkage to a series of electronically unactivated heterocyclic precursors. The substitution reaction constitutes a ring expansion, enabled by the conformationally constrained tether between the nitrile and the heterocycle. Attack of the metallated urea leaving group on the nitrile generates a hydantoin that bridges the polycyclic products. X-ray crystallography reveals ring-dependant strain within the hydantoin.

## Abstract 2

Bicyclic or tricyclic nitrogen-containing heterocyclic scaffolds were constructed rapidly by ring expanding intramolecular S_N_Ar on a series of electronically unactivated heterocyclic precursors.

## Graphical abstract

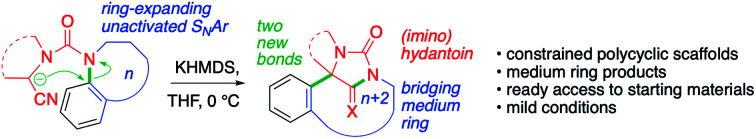

## Introduction

Medium (8–12 membered) ring heterocycles are attractive target structures for medicinal chemistry: their limited conformational mobility limits unfavourable binding entropy while allowing three-dimensional organisation of functionality within the cyclic scaffold.^1,2^ Medium rings lie at the core of a range of natural products having biological activities, such as antiviral, anticancer and anticoagulant agents.^1–3^ Nonetheless, very few marketed pharmaceutical agents contain medium rings.^4,5^ This can be attributed principally to difficulties associated with their synthesis, namely the unfavourable transannular interactions and entropic factors that disfavour the corresponding cyclisation reactions.^6^ Simple new synthetic routes to functionally diverse medium ring heterocycles capable of further functionalisation are therefore of particular utility.^7^

Ring expansion is a valuable strategy for the formation of medium rings, avoiding the difficulties associated with unfavourable cyclisations. Ring-expanding nucleophilic substitutions have been at the forefront of recent developments in this area.^8^ We previously reported an operationally simple approach to the expansion of nitrogen heterocycles (ring size *n*) into medium rings (ring size *n* + 3 or *n* + 4) by exploiting conformationally directed intramolecular nucleophilic aromatic substitution reactions.^9–11^ Previous work employed powerfully basic urea-substituted α-aryl organolithium derivatives or allyllithium derivatives as carbon nucleophiles towards the unreactive, unactivated aromatic rings.^12,13^

**Scheme 1:**
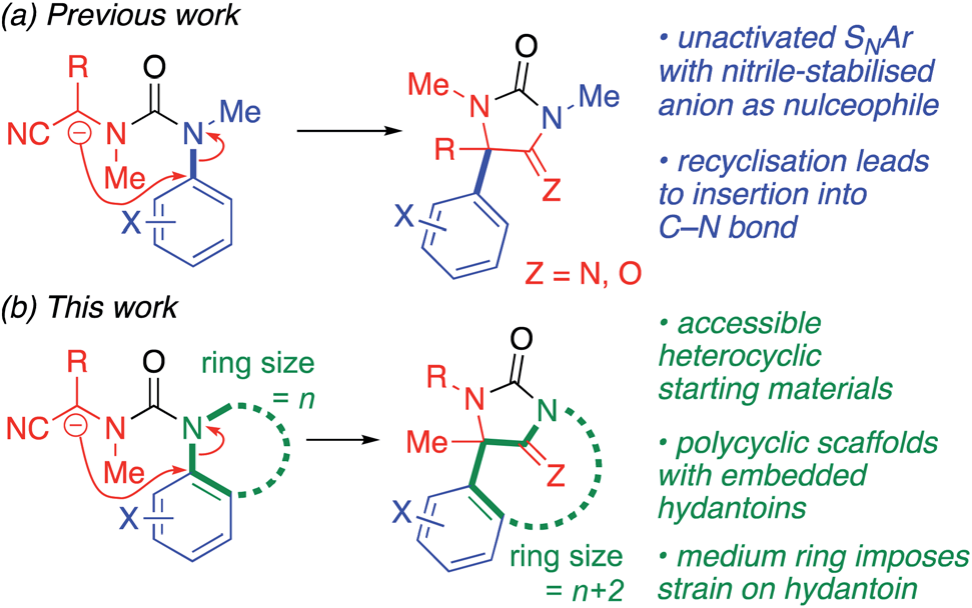
Insertion reactions of nitrile-stabilised anions into aromatic C–N bonds (a) in acyclic systems and (b) leading to the ring-expanding formation of bridged hydantoins (this work).

C–Arylation using N to C migrations of aromatic rings, in the manner of the Truce–Smiles rearrangement,^14,15^ is turning out to be a promising method for the electronically versatile, transition-metal-free introduction of aromatic substituents,^16^ also to less basic, more versatile enolate-type nucleophiles: we recently demonstrated that amino acids may be arylated stereoselectively in this way.^17–19^ These reactions work because the conformation of substituted ureas (or amides) bearing an *N*-aryl substituent typically favours conformations in which the carbonyl and aryl group lie *trans* across the urea (or amide) linkage.^20–24^ Given this conformational constraint, nitrile-stabilised carbanions^25^ will attack an unactivated C–N bond, directed by the conformational preference of a urea function (Scheme 1a), yielding iminohydantoin and hydantoin products.^26–28^ We now show that the use of such anions in a ring-expansion reaction of nitrogen heterocycles leads to a two-carbon insertion into an aromatic C–N bond, with tandem formation of a bridging (imino)hydantoin ring (Scheme 1b).

## Results and discussion

**Scheme 2:**
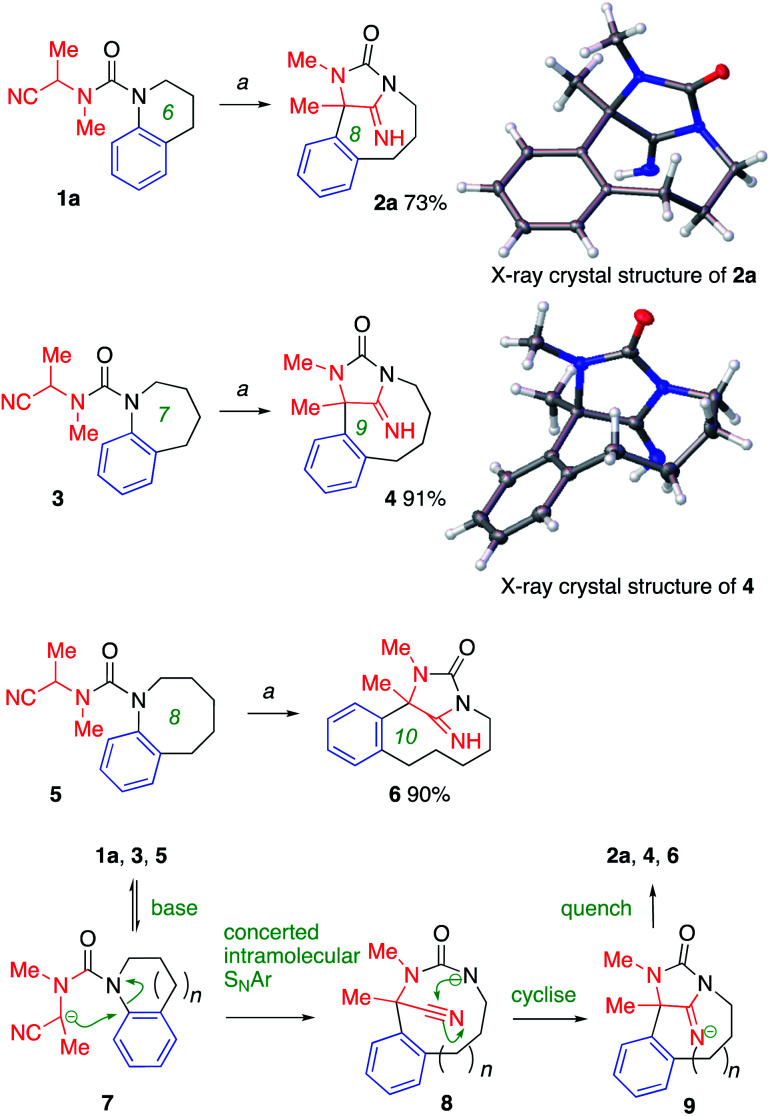
Ring-expansion of metallated nitriles. Conditions: *a* (1) KHMDS (2 equiv.), THF, 0 °C, 0.5–1.5 h; (2) MeOH. CCDC deposition numbers for X-day data: 2a1993726; 41993727.†

A general ring-expansion method utilising this type of Truce–Smiles rearrangement requires an (otherwise unreactive) precursor heterocycle tethered through a conformationally preorganised urea to a nucleophile. We therefore chose to start with the α-cyanourea, 1a, synthesised in three steps from commercially available starting materials. Treatment with base, optimally 2 equiv. KHMDS in THF at 0 °C, initiated a deprotonation–rearrangement-cyclisation cascade that led directly to the tricyclic iminohydantoin 2a in 73% yield (Scheme 2), the structure of which was confirmed by X-ray crystallography. Similar ring-expanding rearrangements to remarkable tricyclic products 4 and 6 occurred in even higher yield, starting from the 7- and 8-membered heterocycles 3 and 5.

Deprotonation by KHMDS must generate from each of these starting materials, at least to some extent, the anion 7, which attacks the adjacent electron-rich aromatic ring in a conformationally enforced intramolecular S_N_Ar reaction (Scheme 2). The lack of electron-withdrawing substituent on the arene does not stop this reaction taking place, and we propose that this proceeds by the partially concerted addition–elimination mechanism that is typical for S_N_Ar reactions of less activated arenes.^17,29–31^ The product urea anion 8 cyclises onto the cyano group^25^ to generate an iminohydantoin anion 9, which is protonated to yield the product.

**Fig. 1:**
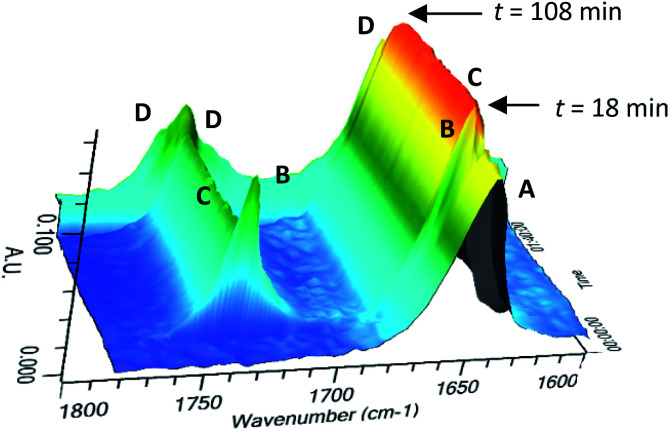
Time-course of the reaction of 3 with KHMDS followed at −5 °C in THF by *in situ* infra-red spectroscopy. A: 3 in THF at −5 °C; B: (*t* = 18 min) KHMDS (2 equiv.) added; C: reaction mixture at −5 °C. D: (*t* = 108 min): reaction quenched by addition of MeOH.

The time course of the reaction of 3 with KHMDS was followed by *in situ* infra-red spectroscopy (ReactIR) at −5 °C (Fig. 1 and ESI†). The carbonyl absorption of 3 at 1654 cm^−1^ (A) shifted transiently to 1663 cm^−1^ (B) on addition of KHMDS (*t* = 18 min) and then immediately (within 15 s) back to 1660 cm^−1^ (C) for the remainder of the reaction period. The shift was accompanied by the appearance of an absorption at 1737 cm^−1^ (B, C). The final quench (*t* = 108 min) forms the product 4 with absorptions at 1662, 1736 and 1743 cm^−1^ (D). The absorptions of C were assigned to anion 9 by comparison of C with the IR spectrum produced on treatment of 4 with KHMDS and are analogous to those seen in related unbridged hydantoins, but at frequencies about 15 cm^−1^ higher. The deprotonation to give 7 and rearrangement to 8 and cyclisation to 9 appear to be instantaneous under these conditions, as no absorptions typical of urea anions (7 and 8) were detected. The product resulting from direct protonation of 8 was not observed.

**Scheme 3:**
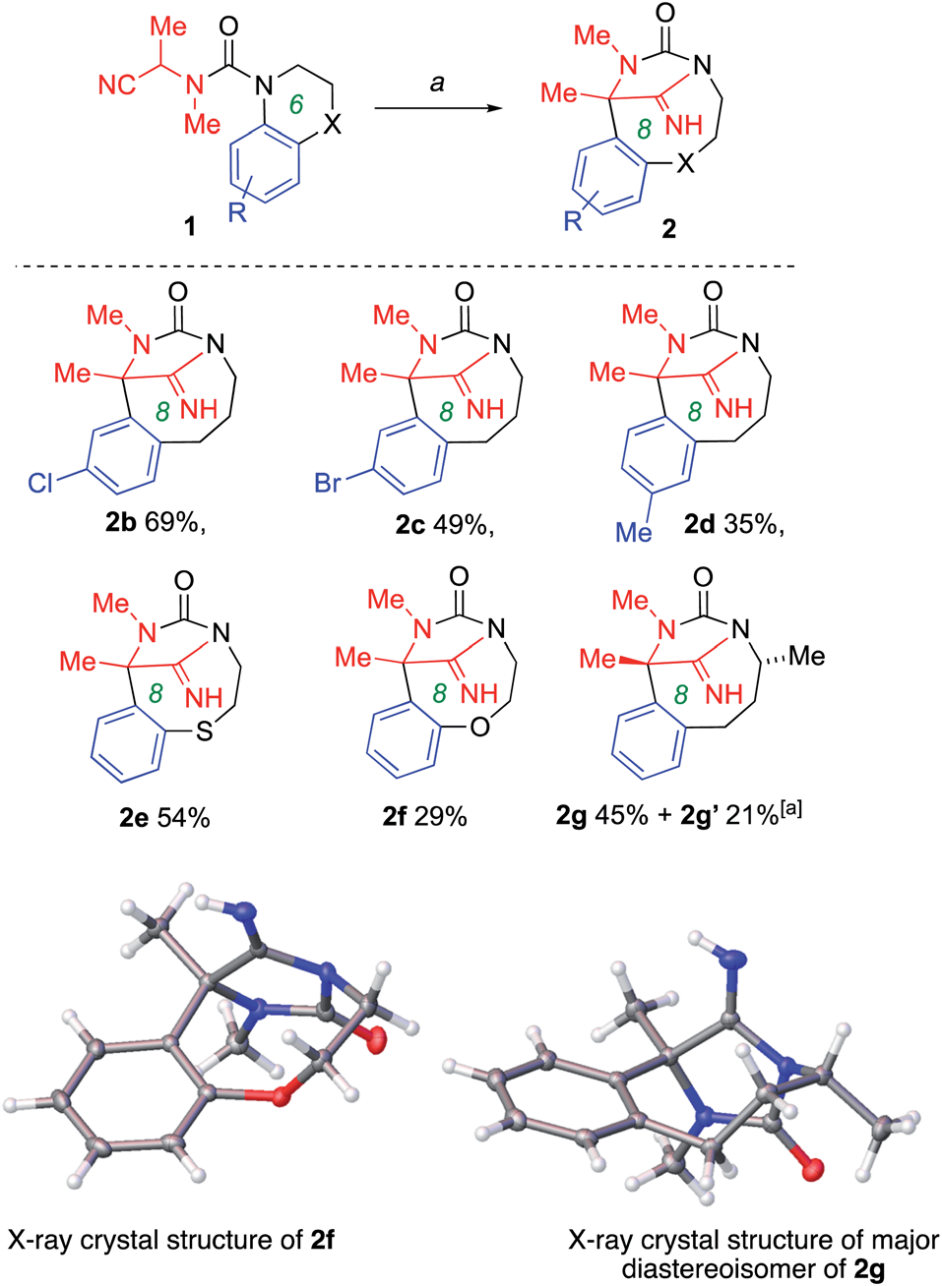
Ring-expansion of substituted tetrahydroquinolines. Conditions: *a* (1) KHMDS (2 equiv.), THF, 0 °C, 1–2 h; (2) MeOH.^*a*^2g′ is the diastereoisomer of 2g shown. CCDC deposition numbers for X-day data: 2f1993728; 2g1993729.†

The versatility of the ring-expansion method was explored by varying the starting tetrahydroquinoline scaffold 1. Related structures 2b–2d (Scheme 3) were formed in moderate yield from starting materials carrying either electron-donating or electron-withdrawing substituents on the migrating aromatic ring, consistent with the tolerance of related rearrangements of ureas to both electron-rich and electron-deficient rings. Substituted rings in which one of the atoms of the tether was replaced with a heteroatom also migrated, to give the bridged thiazocane and oxazocane, 2e and 2f, although competing elimination reactions reduced the yield of these reactions.^32^ Substitution on the aliphatic ring of the starting material was tolerated, with 2-methylquinoline derivative 1g giving the ring-expanded product 2g as a mixture of diastereoisomers, the major one being identified by X-ray crystallography. For medium ring products 2a, 2e and 2f cleavage of the urea also occurred and the starting tetrahydroquinoline was isolated in low yield.

**Scheme 4:**
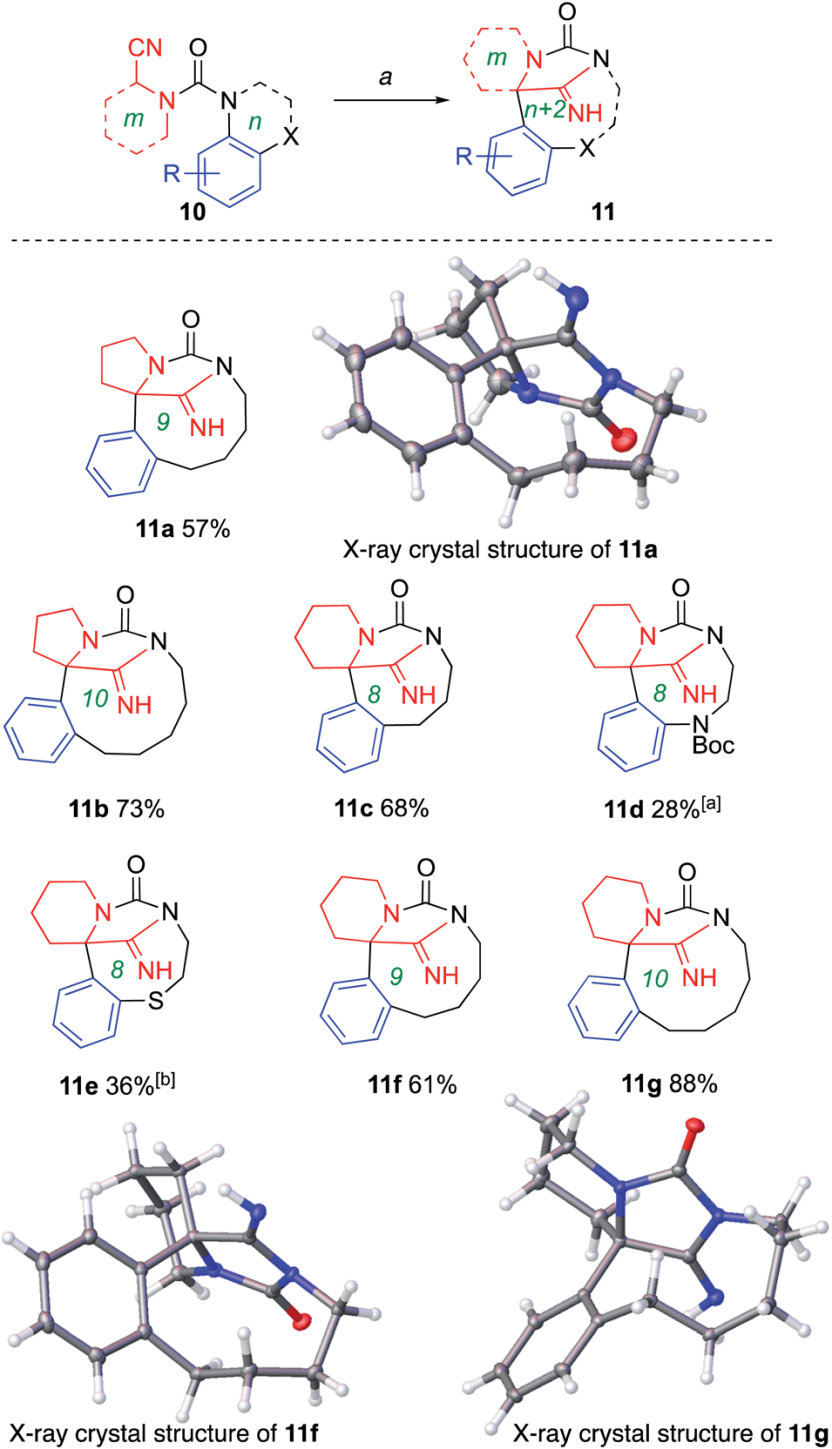
Ring expansion to polycyclic scaffolds. Conditions: *a* (1) KHMDS (2 equiv.), THF, 0 °C, 1–1.5 h; (2) MeOH. ^*a*^Accompanied by products of urea cleavage. ^*b*^A lactam resulting from oxidation of 10 was isolated in 26% yield. CCDC deposition numbers for X-day data: 11a1993730; 11f1993731; 11g1993732.†

**Scheme 5:**
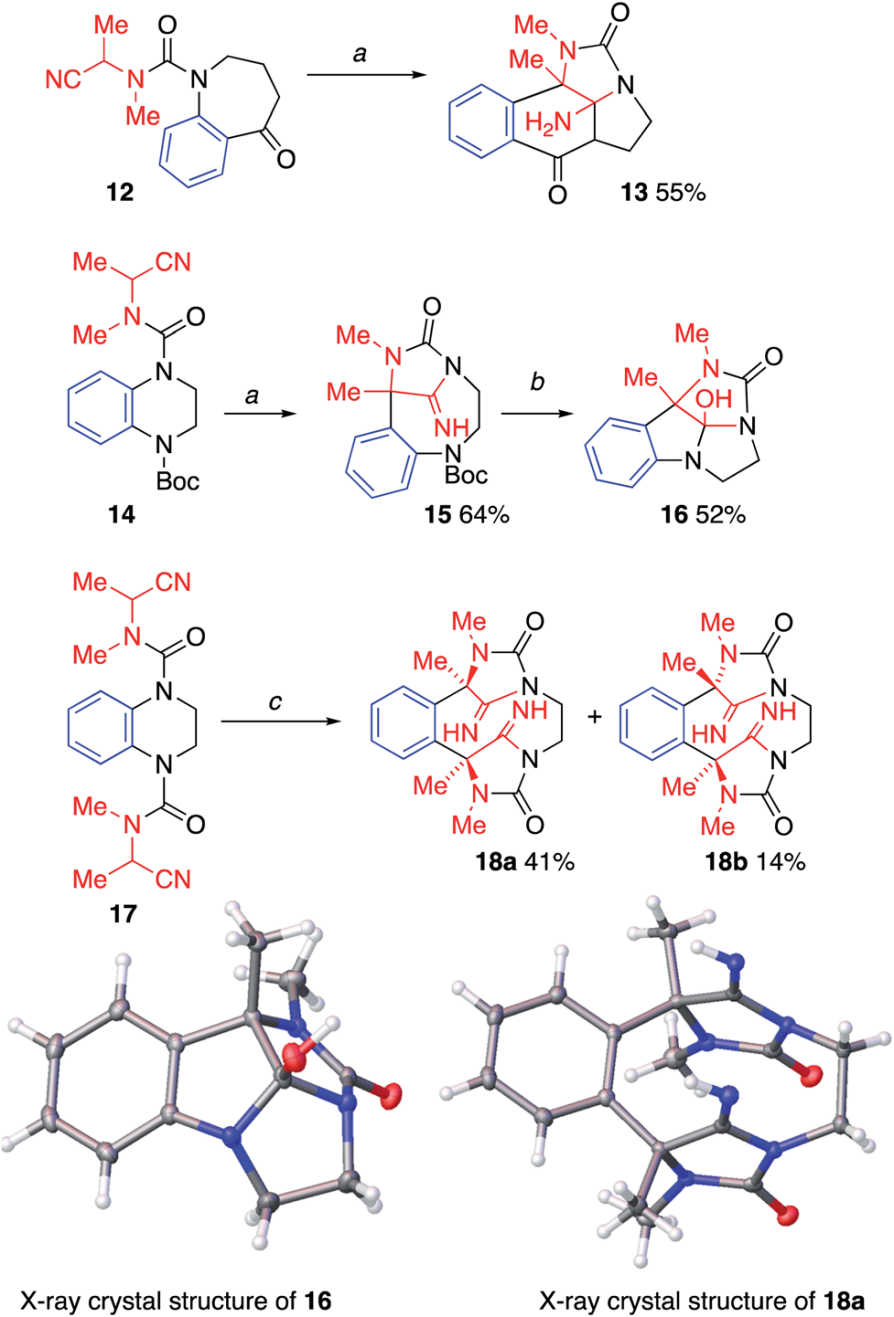
Ring-expansion of substituted tetrahydroquinolines. Conditions: *a* (1) KHMDS (2 equiv.), THF, 0 °C, 1–1.5 h; (2). MeOH; *b* 2 M HCl : MeOH (1 : 1), reflux, 44 h. *c* (1) KHMDS (4 equiv.), THF, 0 °C, 5 h. (2) MeOH. CCDC deposition numbers for X-day data: 161993733; 18a1993734.†

Cyclic aminonitriles are readily available commercially, or by reductive cyanation. Molecular scaffolds with further complexity were formed by employing these cyclic aminonitriles as starting materials. Thus 2-cyanopyrrolidine and piperidine carbamoyl chloride derivatives were coupled with benzo-fused nitrogen heterocycles to provide ureas 10a–g. Treatment with base generated anions that rearranged to the polycyclic structures 11a–g (Scheme 4). Yields were ring-size dependent, with the general trend, parallel to that seen in Schemes 1 and 2, that larger rings formed in greater yield than the smaller. Formation of 11d was accompanied by products of urea cleavage. With 11e, no elimination products were noted but a by-product resulting from oxidation of 10 a to the cyano group and elimination of cyanide was isolated in 26% yield (see ESI†).

More elaborate ring systems were made by incorporating additional nucleophilic sites into the starting structures (Scheme 5). For example, 12, which is the keto-derivative of 3a, underwent a tandem migration-cyclisation to give 13, in which the ketone's enolate has attacked the iminohydantoin, presumably to relieve transannular strain across the intermediate 10-membered ring. A similar transannular interaction was enabled by ring expansion of the benzopiperazine derivative 14. The initial stable Boc-protected product 15 was formed in good yield, but deprotection of the Boc group and concomitant hydrolysis of the imine led directly to the polycycle 16.

An alternative benzopiperazine-derived staring material, 17, incorporates a second cyanoalkyl group that is capable of deprotonation. And indeed, treatment of 17 with four equivalents of KHMDS induced two successive ring expansions, enlarging the six-membered ring of the starting material twice, to form 18 with a peripheral 12-membered ring bridged by two iminohydantoins. Two diastereoisomers were formed in a 74 : 26 ratio, the minor (18b) being the *C*_2_ symmetric diastereoisomer (racemic by HPLC on chiral stationary phase, see ESI†) and the major (18a) being the meso compound (identified by X-ray crystallography).

**Scheme 6:**
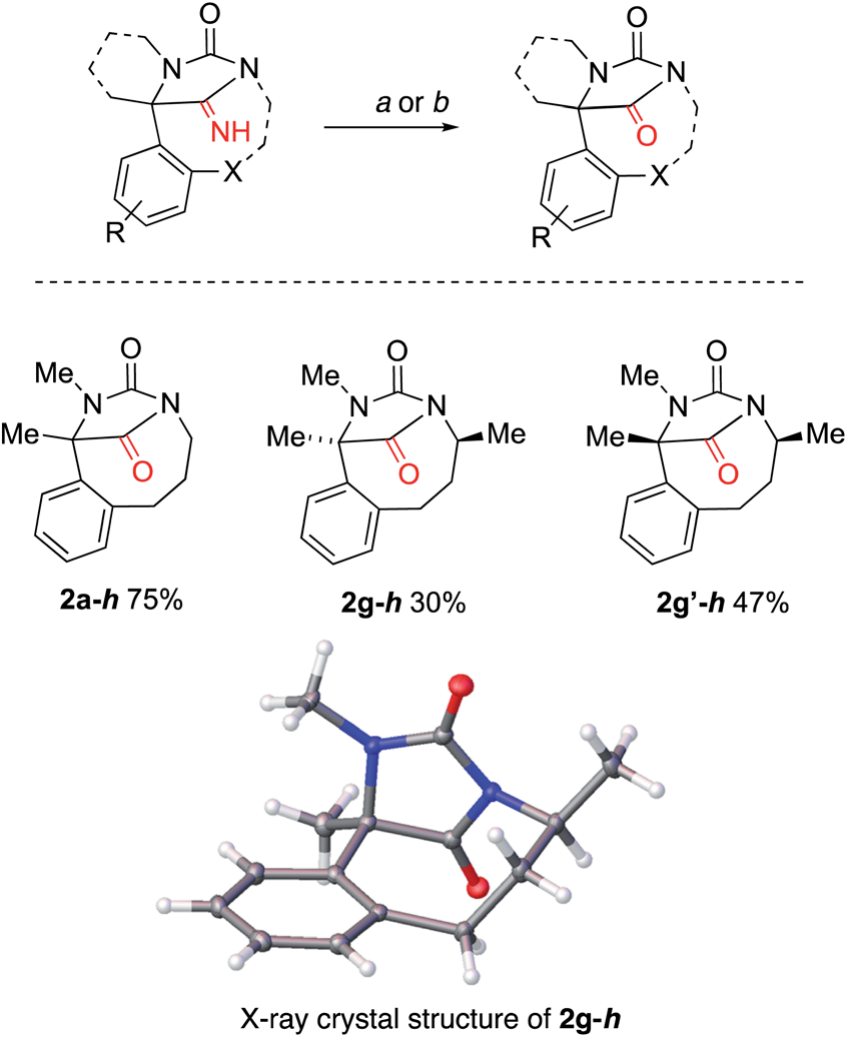
Hydrolysis of fused iminohydantoins to yield hydantoins. Conditions: *a* 2 M HCl : TFA (1 : 9), μW, 120 °C, 2 h; *b* 2 M HCl : MeOH (1 : 1), 70 °C, 44 h. CCDC deposition number for X-day data: 2g–h1993735.†

Both iminohydantoins and hydantoins^33,34^ (including bridged structures^32^) exhibit various biological activities. Representative iminohydantoins 2a, 2g and 2g′ were transformed into their hydantoin congeners by hydrolysis with acid. The iminohydantoins were either refluxed in 1 : 1 mixture of methanol and hydrochloric acid or irradiated in a microwave in a mixture of hydrochloric acid with trifluoroacetic acid, yielding hydantoins in moderate to good yield (Scheme 6).

Typical hydantoins are stable, more or less planar rings. Those formed by this insertion reaction contain a bridgehead nitrogen that is formally sp^2^-hybridised, but for which full planarity would contravene Bredt's rule.^35,36^ To explore the effect of imposing the constraint of a medium ring on the geometry of the hydantoin, bond angles in the X-ray crystal structures of the iminohydantoin and hydantoin products were compared with those from X-ray crystal structures of a sample of 5 simple, monocyclic hydantoins.^37–40^Table 1 tabulates some of these data according to ring size. The most notable structural change occurred at N3. In the monocyclic hydantoins, this atom is planar, but decreasing the ring size of the tether (which links through this atom) led to greater pyramidalization of this N atom. The size of the medium ring linkage had little effect on N1. Both C2 and C4 remained essentially planar even in the smaller, more strained rings. However, increased ring strain had a significant effect on the ^13^C NMR shift of C2, which moved upfield with increasing strain.
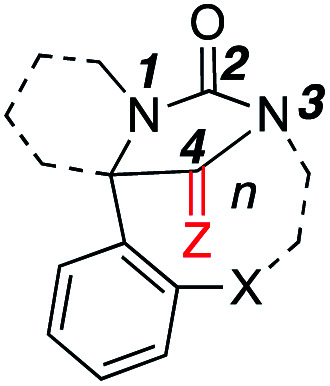


**Table 1:** Structural parameters

Ring size *n*	N1 ΔΣ*θ*^a^	C2 ΔΣ*θ*^a^	C2 Δ*δ*^b^	N3 ΔΣ*θ*^a^	C4 ΔΣ*θ*^a^	C4 Δ*δ*^b^
—c	1.2	0.0	0.0^d^	0.1	0.0	0^e^
10^f^	2.9^g^	0.0	−0.9	2.4	0.2	4.6
9^h^	2.1^g^	0.0	−3.9	4.6	0.1	1.3
8^i^	2.1	0.0	−5.9	13.4	0.0	0.6

a ΔΣ*θ* = deviation from planarity = 360° – sum of bond angles.

b Δ*δ* = Change in chemical shift from the average value of 2 representative hydantoins.

c Values for representative monocyclic hydantoins (structures given in ESI) taken from ref. ^37–40^.

d *δ* = 155.3 ppm (average of 2 values^37^).

e *δ* = 173.7 ppm for iminohydantoins only (average of 2 values^37^).

f Data taken from X-ray crystal structures of 11g and 18a.

g Excluding structures where this atom lies at a ring junction.

h Data taken from X-ray crystal structures of 4, 11a, 11f.

i Data taken from X-ray crystal structures of 2a, 2f, 2g.

## Conclusions

In summary, a urea linkage between an aminonitrile and a benzo-fused nitrogen heterocycle enables the anion of the aminonitrile to act as a nucleophile in an S_N_Ar substitution on the unactivated arene of the heterocycle. A ring expansion by two atoms results, giving an iminohyantoin-bridged medium ring in which the nitrile anion has formally inserted into the ArC–N bond of the heterocycle. The product iminohydantoin ring of the polycycle product may be hydrolysed to a hydantoin, giving functionalised bridged and caged structures of potential utility in medicinal chemistry lead generation.

## Conflicts of interest

There are no conflicts to declare.

## Supplementary Material

SC-012-D0SC06188C-s001

SC-012-D0SC06188C-s002
