# Supplementary material for: Hydantoin-bridged medium ring scaffolds by migratory insertion of urea-tethered nitrile anions into aromatic C–N bonds
Source: Chem Sci. 2020 Dec 14;12(6):2091–6. doi: 10.1039/d0sc06188c (PMC8179327; doi:10.1039/d0sc06188c)
Supplement: SC-012-D0SC06188C-s001 [file SC-012-D0SC06188C-s001.pdf]

Electronic Supplementary Material (ESI) for Chemical Science.
This journal is © The Royal Society of Chemistry 2020

Supporting Information

Hydantoin-bridged medium ring scaffolds by migratory insertion of urea-tethered nitrile anions into aromatic C-N bonds

Makenzie J. Millward, Emily Ellis, John W. Ward, Jonathan Clayden.*

Contents

1. Experimental 3
   - 1.1. General Directions 3
   - 1.2. Analytical Analysis 3
   - 1.3. Literature Known starting Materials 5
2. Synthetic Procedures 5
   - 2.1. General Procedures 5
   - 2.2. Starting Material Synthesis 7
   - 2.3. Ring Expansion Products 22
   - 2.4. Hydrolysis Products 40
   - 2.5. *In situ* ReactIR Experimental Data 44
3. Structural Parameters Raw Data 47
4. ^{1}H and ^{13}C NMR Spectra Products 50
5. X-Ray Crystal Data 75
6. References 86

1. Experimental

1.1. General Directions

Chemicals were purchased (unless otherwise stated) at highest commercial quality and used as received. Non-anhydrous solvents were purchased (unless otherwise stated) at the highest commercial quality and used as received. Anhydrous DCM, MeCN and THF were obtained by passage through a column of activated alumina (A-2). Anhydrous Et_2O was stored over 3 Å molecular sieves and purchased from Acros and used as received. NEt_3 was purchased from Fischer Scientific and stored over KOH pellets. Reactions requiring anhydrous conditions were performed under N_2 and in flame dried or oven-dried glassware. Liquid reagents, solutions or solvents were added *via* syringe through rubber suba-seal. Reactions run in a microwave oven were completed on a Biotage Initiatior+.

1.2. Analytical Analysis

Chromatography: Flash column chromatography was performed on an automated Biotage Isolera^{TM} Spektra Four using gradient elution on prepacked silica gel Biotage SNAP Ultra columns or Biotage Sfär columns or by manual methods using silica gel [Merck, 230-400 mesh (40-63 µm)]. Solvents for flash column chromatography and TLC are listed as volume:volume percentages.

R_f: Reactions were monitored by TLC on Kieselgel $60F_{254}$ (Merck), with UV fluorescence (254 & 366 m), and either by staining with potassium permanganate solution/Δ, ninhydrin/Δ or Seebach stain/Δ. Solvents for TLC are listed as volume:volume percentages.

NMR: NMR data was collected on a 400 MHz Bruker BioSpin GmbH equipped with a Z108618 0884 (PA BBO 400S1 BBF-H-D-05 Z) probe, or on a cryo500MHz Bruker BioSpin GmbH,

equipped with a Z123841 0005 (CP DCH 500S1 C/H-D-05-Z) probe. All NMR experiments were run at 25 °C unless otherwise stated. Spectra collected were analysed and processed using MestReNova 11.0.2-18153. Chemical shifts (δ) are quoted in parts per million (ppm) and referenced to the residual proton signals of $CHCl_3$, DMSO or MeOH (δ 7.26, 2.50 or 3.31 ppm respectively). ^{1}H coupling constants are reported in Hz. Data are reported as follows: chemical shift, multiplicity (s = singlet, d = doublet, t = triplet, q = quartet, quint. = quintet, m = multiplet, dd = doublet of doublets etc …), coupling constants, integration and assignment. 2D experiments of COSY, HSQC and HMBC were used in the assignment of both ^{1}H and ^{13}C NMRs.

HRMS: Mass spectra were obtained by the University of Bristol mass spectrometry service on a Bruker Daltronics MicrOTOF 2 mass spectrometer (ESI – electronspray ionisation) with only molecular ions $[M+H]^+$ and $[M+Na]^+$ reported.

mp: Melting points were measured on a Scientific SMP10 melting point apparatus and are uncorrected.

FT-IT: IR spectra were recorded using a Perkin Elmer (Spectrum One) FT-IR spectrometer (ATR sampling accessory) and were recorded neat. Only strong and selected absorbances are reported.

Crystal for X-ray Sample Preparation: Crystals suitable for X-ray were grown by dissolving the sample in a solvent which it is reasonably soluble in (CH_2Cl_2, MeOH, EtOH, $CHCl_3$, $CDCl_3$) and a second chamber placed outside containing a solvent which the compound is not soluble in (Et_2O, *n*-hexane, *n*-pentane). The solvents slowly mixed causing crystallisation.

1.3. Literature Known Starting Materials:

Urea **1f** *N*-(1-cyanoethyl)-*N*-methyl-2,3-dihydro-4*H*-benzo[*b*][1,4]oxazine-4-carboxamide was synthesised as reported by B.P. Corbet, *et. al.*[1] 7-bromo-1,2,3,4-tetrahydroquinoline was synthesised as reported by M. F. T. Koehler *et. al.,*[2] and used to synthesis urea **1c**. 2-Cyanopyrrolidine-1-carbonyl chloride was synthesised as reported by J. Mas-Roselló *et. al.,*[3] and was used to synthesis ureas 9**a** and **9b**. 2,3,4,5-tetrahydro-1*H*-benzo[*b*]azepine and 3,4,5,6-tetrahydrobenzo[*b*]azocin-2(1*H*)-one were synthesised in accordance with literature procedures by Kenwright and Ren *et. al.,*[4,5] and used in the synthesis of ureas **3a**, **5a**, **9a**, **9b**, **9f** and **9g**.

2. Synthetic Procedures

2.1. General Procedures

General Procedure 1 (GP1) – Carbamoyl Chloride Synthesis:

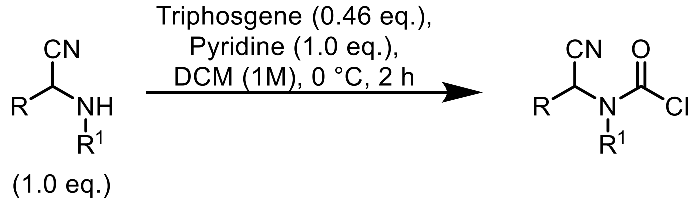

Under an atmosphere of N_2 a solution of triphosgene (0.46 eq.) in anhydrous DCM (1M) was cooled to 0 °C. Anhydrous pyridine (1.0 eq.) was added dropwise followed by the amine (1.0 eq.) whilst maintaining 0 °C. The reaction mixture was stirred for 2 h at 0 °C. The reaction mixture was quenched with HCl (1M) and allowed to warm to room temperature. The reaction mixture was diluted with DCM (30 mL) and washed with HCl (1M, 2 x 30 mL). The combined aqueous layers were extracted into DCM (2 x 30 mL). The combined organic layers

were washed with sat. aq. $NaHCO_3$ (15 mL). The organic layer was dried over $MgSO_4$, filtered and the solvent removed under reduced pressure to afford the crude carbamoyl chloride.

General Procedure 2 (GP2) – Urea synthesis:

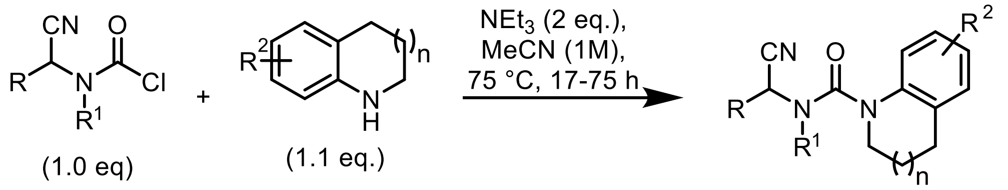

Under an atmosphere of N_2 the amine (1.1 eq.) and anhydrous triethylamine (2.0 eq.) were added to a solution of the carbamoyl chloride (1.0 eq.) in anhydrous MeCN. The reaction mixture was heated to 75 °C. The reaction mixture was cooled to room temperature and sat. aq. $NaHCO_3$ (5 mL) was added. The aqueous layer was extracted into DCM (2 x 20 mL). The combined organic layers were washed with brine (20 mL), dried over Na_2SO_4, filtered and the solvent removed under reduced pressure. Purification via flash column chromatography afforded the title compound.

General Procedure 3 (GP3) – KHMDS Promoted Ring-Expansion:

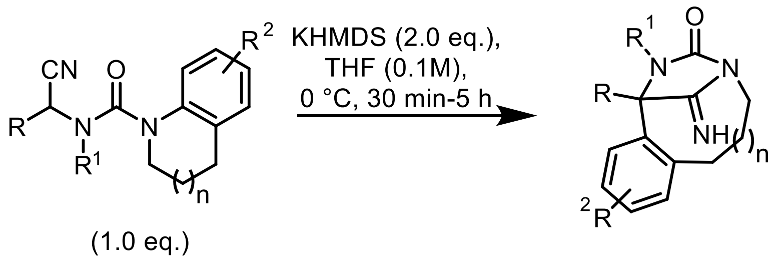

Under an atmosphere of N_2, the urea (1.0 eq.) was dissolved in anhydrous THF (0.1 M) and cooled to 0 °C. KHMDS (1M in THF, 2 eq.) was added dropwise whilst maintaining 0 °C. The reaction mixture was stirred at 0 °C. The reaction mixture was quenched with MeOH (0.5 mL), allowed to warm to room temperature and diluted with EtOAc (20 mL). The organic layer was

washed with sat. aq. $NaHCO_3$ (15 mL) and the aqueous layer was further extracted into EtOAc (2 x 20 mL). The combined organic layers were dried over $MgSO_4$, filtered and the solvent removed under reduced pressure to afford the crude ring expanded product. Purification via flash column chromatography afforded the desired compound.

General Procedure 4 (GP4) – Hydrolysis of iminohydantoin with TFA and HCl:

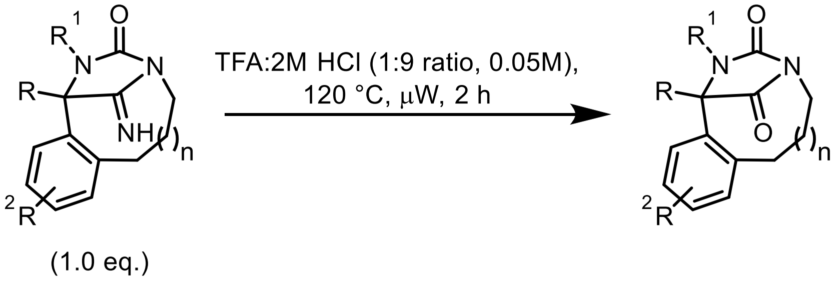

The medium ring (1.0 eq.) was dissolved in TFA:2M HCl (1:9 ratio, 0.05 M) in a microwave vial. The vial was sealed and heated in a microwave reactor at 120 °C. The reaction was basified (pH > 7) with sat. aq. $NaHCO_3$. The product was extracted into DCM. The combined organic layers were dried over Na_2SO_4, filtered and the solvent removed under reduced pressure affording the title compound.

2.2. Starting Material Synthesis

(1-Cyanoethyl)(methyl)carbamic chloride:

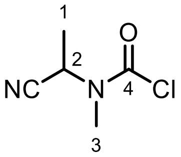

DL-lactonitrile (1.0 mL, 14.1 mmol) and excess $MgSO_4$ were cooled to 0 °C. Methylamine (5.20 mL, 42.2 mmol, 33 wt% in EtOH), was added and the reaction mixture was stirred for 19 h warming to room temperature. The reaction mixture was filtered and the volatiles removed

under reduced pressure to afford the crude title product (745 mg, 63%) as a yellow oil. (Analytical data matched literature.[1])

According to **GP1**, triphosgene (1.01 g, 3.40 mmol), anhydrous pyridine (0.63 mL, 7.82 mmol) and 2-(methylamino)propanenitrile (658 mg, 7.82 mmol) afforded the crude title product (765 mg, 67%) as an orange oil. **^{1}H NMR** (400 MHz, $CDCl_3$): δ_H 5.33 (q, J = 7.2, 1H, H2), 3.20 (s, 3H, H3), 1.56 (d, H = 7.2, 3H, H1). **^{13}C NMR** (101 MHz, $CDCl_3$): δ_C 150.0 (C4), 116.5 (CN), 44.3 (C2), 33.9 (C3), 17.0 (C1).

2-Cyanopiperidine-1-carbonyl chloride:

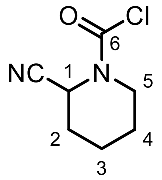

According to **GP1**, triphosgene (1.80 g, 6.07 mmol), anhydrous pyridine (1.06 mL, 13.2 mmol) and piperidine-2-carbonitrile (1.45 g, 13.2 mmol) were stirred for 3 h at 0 °C, affording the crude title product (1.55 g, 68%) as a brown oil. **^{1}H NMR** (400 MHz, $CDCl_3$): δ_H 5.49-5.46 (m, 1H, H1), 4.36 (ddd, J = 13.6, 4.4, 2.3, 1H, CH_2), 3.35 (td, J = 13.6, 2.7, 1H, CH_2), 2.09-1.77 (m, 5H, 3 x CH_2), 1.58-1.50 (m, 1H, CH_2). **^{13}C NMR** (101 MHz, $CDCl_3$): δ_C 149.7 (C6), 116.2 (CN), 46.6 (CH_2), 45.8 (C1), 28.6 (CH_2), 24.9 (CH_2), 20.3 (CH_2).

***N*-(1-cyanoethyl)-*N*-methyl-3,4-dihydroquinoline-1(2*H*)-carboxamide (1a):**

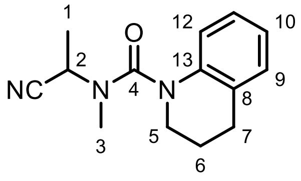

According to **GP2**, (1-cyanoethyl)(methyl)carbamic chloride (357 mg, 2.44 mmol), 1,2,3,4-tetrahydroquinoline (0.34 mL, 2.68 mmol) and triethylamine (0.68 mL, 4.87 mmol) were

refluxed at 75 °C or 42 h. Purification via flash column chromatography eluting with EtOAc:PE (3:10 – 2:3) afforded the title compound (448 mg, 1.84 mmol, 76%) as a brown solid. **R*f*** = 0.19 (1:4 EtOAc:PE). **^{1}H NMR** (400 MHz, $CDCl_3$): δ_H 7.15-7.11 (m, 2H, ArH), 7.00-6.95 (m, 2H, ArH), 5.21 (q, J = 7.3, 1H, H2), 3.67 (ddd, J = 12.6, 6.7, 5.9, 1H, H5a), 3.57 (ddd, J = 12.6, 6.9, 5.9, 1H, H5b), 2.76 (t, J = 6.7, 2H, H7), 2.69 (s, 3H, H3), 2.02-1.95 (m, 2H, H6), 1.54 (d, J = 7.3, 3H, H1). **^{13}C NMR** (101 MHz, $CDCl_3$): δ_C 160.3 (C4), 140.3 (C13), 129.7 (C8), 129.5 (ArH), 127.2 (ArH), 123.6 (ArH), 120.7 (ArH), 119.0 (CN), 46.0 (C5), 44.5 (C2), 330 (C3), 27.3 (C7), 24.2 (C6), 17.5 (C1). **HRMS m/z** (ESI$^+$): calcd. For $C_{14}H_{18}N_3O$ [M+H] Calculated. 244.1444. Found. 244.1455. **IR ν$_{max}$** (ATR)/cm^{-1}: 2995, 2930, 2855, 2239, 1633. **m.p.** 84-88 °C.

***N*-(1-cyanoethyl-*N*-methyl-2,3,4,5-tetrahydro-1*H*-benzo[*b*]azepine-1-carboxamide (3):**

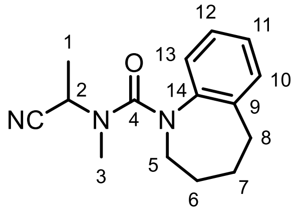

According to **GP2**, (1-cyanoethyl)(methyl)carbamic chloride (166 mg, 1.13 mmol), 2,3,4,5-tetrahydro-1*H*-benzo[*b*]azepine (183 mg, 1.24 mmol) and triethylamine (0.31 mL, 2.26 mmol) were refluxed at 75 °C for 43 h. Purification via flash column chromatography eluting with EtOAc:PE (1:4 – 2:3) afforded the title compound (131 mg, 0.509 mmol, 45%) as a purple solid. **R*f*** = 0.34 (1:4 EtOAc:PE). **^{1}H NMR** (400 MHz, $CDCl_3$): δ_H 7.24-7.11 (m, 3H, ArH), 6.92 (dd, J = 7.6, 1.6, 1H, ArH), 5.18 (q, J = 7.3, 1H, H2), 3.92-3.42 (m, br, 2H, H5), 2.81-2.78 (m, 2H, H7), 2.36 (s, 3H, H3), 1.80-10.74 (m, 2H, CH_2), 1.73-1.56 (m, 2H, CH_2), 1.41 (d, J = 7.3, 3H, H1). **^{13}C NMR** (101 MHz, $CDCl_3$): δ_C 160.3 (C4), 144.8 (Ar), 138.5 (Ar), 130.7 (ArH, br), 127.7 (ArH), 126.6 (ArH), 124.9 (ArH), 48.9 (C5), 44.3 (C2, br), 35.2 (C7), 32.0 (CH_2, br), 30.5 (CH_2), 17.1 (C1, br).

HRMS m/z (ESI^+): calcd. For $C_{15}H_{20}N_3O$ [M+H] Calculated. 258.1601. Found. 258.1590. **IR ν_{max}** (ATR)/cm^{-1}: 2995, 2930, 2855, 2239, 1633. **m.p.** 114-116 °C.

_N_-(1-cyanoethyl)-_N_-methyl-3,4,5,6-tetrahydrobenzo[_b_]azocine-1(2_H_)-carboxamide (5):

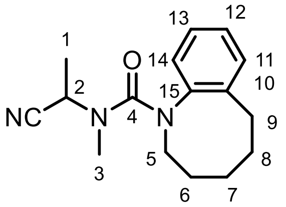

According to **GP2**, (1-cyanoethyl)(methyl)carbamic chloride (124 mg, 0.846 mmol), 1,2,3,4,5,6-hexahydrobenzo[_b_]azocine (150 mg, 0.930 mmol) and triethylamine (0.26 mL, 1.86 mmol) were refluxed at 75 °C for 23 h. The title product (215 mg, 0.830 mmol, 94%) was obtained as a brown oil. **R_f** = 0.58 (1:3 EtOAc:PE). **^{1}H NMR** (400 MHz, $CDCl_3$): δ_H 7.27-7.21 (m, 3H, ArH), 7.01-6.97 (m, 1H, ArH), 5.17 (q, J = 7.2, 1H, H2), 3.71-3.48 (m, br, 2H, H5), 2.69 (td, J = 6.1, 2.0, 2H, H9), 2.43 (s, 3H, H3), 1.76-1.66 (m, br, 2H, H8), 1.55-1.43 (m, br, 4H, H6/7), 1.31 (d, J = 7.2, 3H, H1). **^{13}C NMR** (101 MHz, $CDCl_3$): δ_C 161.9 (C4), 142.4 (Ar), 141.9 (Ar), 130.6 (ArH), 128.2 (ArH), 128.0 (ArH), 127.6 (ArH), 118.9 (CN), 54.5 (C5), 44.6 (C2), 32.0 (C3), 31.8 (C9), 31.7 (C8), 26.9 (CH_2), 26.1 (CH_2), 17.3 (C1). **HRMS m/z** (ESI^+): calcd. For $C_{16}H_{22}N_3O$ [M+H] Calculated. 272.1682. Found. 272.1748. **IR ν_{max}** (ATR)/cm^{-1}: 2925, 2855, 2239, 1641.

7-Chloro-_N_-(1-cyanoethyl)-_N_-methyl-3,4-dihydroquinoline-1(2_H_)-carboxamide (1b):

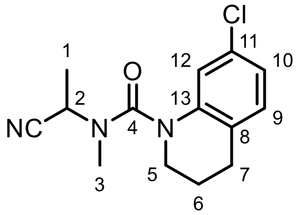

According to **GP2**, (1-cyanoethyl)(methyl)carbamic chloride (131 mg, 0.894 mmol), 7-chloro-1,2,3,4-tetrahydroquinoline (150 mg, 0.895 mmol) and triethylamine (0.25 mL, 1.82 mmol)

were refluxed at 75 °C for 63 h. Purification via flash column chromatography eluting with EtOAc:PE (1:3 – 1:0) afforded the title compound (144 mg, 0.518 mmol, 58%) as a pink solid. **R_f** = 0.50 (3:7 EtOAc:PE). **^{1}H NMR** (400 MHz, $CDCl_3$): δ_H 7.10-6.98 (m, 1H, ArH), 6.98-6.88 (m, 2H, ArH), 5.18 (q, J = 7.2, 1H, H2), 3.66 (ddd, J = 12.4, 6.9, 5.4, 1H, H5a), 3.53 (ddd, J = 12.4, 7.2, 5.3, 1H, H5b), 2.76 (s, 3H, H3), 2.76-2.72 (m, 2H, H7), 2.06-1.88 (m, 2H, H6), 1.57 (d, J = 7.2, 3H, H1). **^{13}C NMR** (101 MHz, $CDCl_3$): δ_C 159.8 (C4), 140.8 (Ar), 132.2 (Ar). 130.4 (ArH), 127.0 (Ar), 123.1 (ArH), 120.2 (ArH), 118.5 (CN). 45.8 (C5), 44.2 (C2), 32.8 (C3), 26.6 (C7), 23.4 (C6), 17.2 (C1). **HRMS m/z** (ESI^+): calcd. For $C_{14}H_{17}ClN_3O$ [M+H] Calculated. 278.1055. Found. 278.1058. **IR ν_{max}** (ATR)/cm^{-1}: 2972, 2919, 2843, 2236, 1649. **m.p.** 119-122 °C.

7-Bromo-*N*-(1-cyanoethyl)-*N*-methyl-3,4-dihydroquinoline-1(2*H*)-carboxamide (1c):

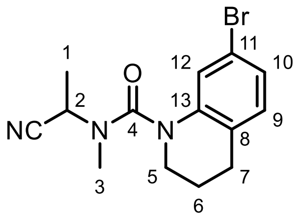

According to **GP2**, (1-cyanoethyl)(methyl)carbamic chloride (23 mg, 0.156 mmol), 7-bromo-1,2,3,4-tetrahydroquinoline (33 mg, 0.156 mmol) and triethylamine (0.03 mL, 0.212 mmol) were refluxed at 75 °C for 42 h. Purification via flash column chromatography eluting with EtOAc:PE (1:3 – 1:0) afforded the title compound (44 mg, 0.137 mmol, 88%) as a brown oil. **R_f** = 0.52 (1:1 EtOAc:PE). **^{1}H NMR** (400 MHz, $CDCl_3$): δ_H 7.11-7.06 (m, 2H, ArH), 6.98 (d, J = 8.1, 1H, ArH), 5.17 (q, J = 7.3, 1H, H2), 3.66 (ddd, J = 12.4, 6.8, 5.5, 1H, H5a), 3.52 (ddd, J = 12.4, 7.2, 5.3, 1H, H5b), 2.75 (s, 3H, H3), 2.72 (td, J = 6.8, 2.4, 2H, H7), 2.01-1.90 (m, 2H, H6), 1.57 (d, J = 7.3, 3H, H1). **^{13}C NMR** (101 MHz, $CDCl_3$): δ_C 159.8 (C4), 141.0 (Ar), 130.7 (ArH), 127.5 (Ar), 125.9 (ArH), 123.1 (ArH), 119.9 (Ar), 118.4 (CN), 45.8 (C5), 44.2 (C2), 32.8 (C3), 26.6 (C7),

23.3 (C6), 17.2 (C1). **HRMS m/z** (ESI$^+$): calcd. For $C_{14}H_{17}BrN_3O$ [M+H] Calculated. 322.0550. Found. 322.0556. **IR ν_{max}** (ATR)/cm^{-1}: 2975, 2921, 2850, 2238, 1645.

***N*-(1-cyanoethyl)-*N*,6-dimethyl-3,4-dihydroquinoline-1(2*H*)-carboxamide (1d):**

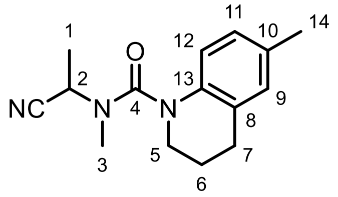

According to **GP2**, (1-cyanoethyl)(methyl)carbamic chloride (200 mg, 1.37 mmol), 6-methyl-1,2,3,4-tetrahydroquinoline (221 mg, 1.50 mmol) and triethylamine (0.38 mL, 2.73 mmol) were refluxed at 75 °C for 60 h. Purification via flash column chromatography eluting with EtOAc:PE (1:9 – 3:2) afforded the title compound (343 mg, 1.33 mmol, 98%) as a brown oil. **R*f*** = 0.11 (1:4 EtOAc:PE). **^{1}H NMR** (400 MHz, $CDCl_3$): δ_H 6.95-6.93 (m, 2H, ArH), 6.87-6.85 (m, 1H, ArH), 5.19 (q, J = 7.3, 1H, H2), 3.66 (ddd, J = 12.6, 6.9, 5.9, 1H, H5a), 3.55 (ddd, J = 12.6, 6.8, 6.0, 1H, H5b), 2.71 (t, J = 6.7, 2H, H7), 2.67 (s, 3H, H3), 2.28 (s, 3H, H14), 2.02-1.91 (m, 2H, H6), 1.54 (d, J = 7.3, 3H, H1). **^{13}C NMR** (101 MHz, $CDCl_3$): δ_C 160.0 (C4), 137.4 (Ar), 132.9 (Ar), 129.7 (ArH), 129.3 (Ar), 127.6 (ArH), 120.4 (ArH), 118.8 (CN), 45.6 (C5), 44.2 (C2), 32.7 (C3), 26.9 (C7), 24.0 (C6), 20.8 (C14), 17.2 (C1). **HRMS m/z** (ESI$^+$): calcd. For $C_{15}H_{20}N_3O$ [M+H] Calculated. 258.1601. Found. 258.1599. **IR ν_{max}** (ATR)/cm^{-1}: 2979, 2941, 2871, 2236, 1643.

***N*-(1-cyanoethyl)-*N*-methyl-2,3-dihydro-4*H*-benzo[*b*][1,4]thiazine-4-carboxamide (1e):**

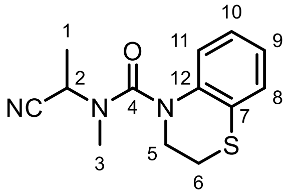

According to **GP2**, (1-cyanoethyl)(methyl)carbamic chloride (110 mg, 0.751 mmol), 3,4-dihydro-2*H*-benzo[*b*][1,4]thiazine (125 mg, 0.827 mmol) and triethylamine (0.21 mL, 1.50 mmol) were refluxed at 75 °C for 40 h. Purification via flash column chromatography eluting with EtOAc:PE (0:1 – 1:0) afforded the title compound (189 mg, 0.724 mmol, 96%) as a yellow oil. **R_f** = 0.34 (3:1 EtOAc:PE). **^{1}H NMR** (400 MHz, $CDCl_3$): δ_H 7.26-7.24 (m, 1H, ArH), 7.11-7.00 (m, 2H, ArH), 6.94 (ddd, J = 8.0, 1.5, 0.5, 1H, ArH), 5.18 (q, J = 7.2, 1H, H2), 4.02 (ddd, J = 13.2, 6.4, 5.3, 1H, H6a), 3.81 (ddd, J = 13.2, 7.0, 5.3, 1H, H6b), 3.27-3.17 (m, 2H, H5), 2.58 (s, 3H, H3), 1.52 (d, J = 7.2, 3H, H1). **^{13}C NMR** (101 MHz, $CDCl_3$): δ_C 159.5 (C4), 140.0 (Ar), 128.5 (ArH), 127.9 (Ar), 126.1 (ArH), 125.2 (ArH), 122.6 (ArH), 118.8 (CN), 44.8 (C6), 44.4 (C2), 33.1 (C3), 30.9 (C5), 17.5 (C1). **HRMS m/z** (ESI^+): calcd. For $C_{13}H_{15}N_3OSNa$ [M+Na] Calculated. 284.0936. Found. 284.0829. **IR ν_{max}** (ATR)/cm^{-1}: 2979, 2928, 2852, 2242, 1642.

***N*-(1-cyanoethyl)-*N*,2-dimethyl-3,4-dihydroquinoline-1(2*H*)-carboxamide (1g):**

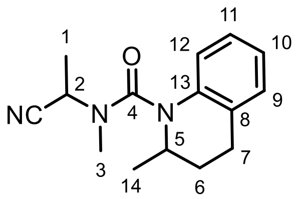

According to **GP2**, (1-cyanoethyl)(methyl)carbamic chloride (181 mg, 1.24 mmol), 2-methyl-1,2,3,4-tetrahydroquinoline (0.20 mL, 1.36 mmol) and triethylamine (0.34 mL, 2.47 mmol) were refluxed at 75 °C for 41 h. Purification via flash column chromatography eluting with

EtOAc:PE (7:13 – 2:3) afforded the title compound (212 mg, 0.824 mmol, 67%) as a mixture of 2 diastereoisomers as a yellow oil. **R*f*** = 0.71/0.62 (3:7 EtOAc:PE). **dr** = 1.0:1.5.

Diastereoisomer 1: ^{1}H NMR (400 MHz, $CDCl_3$): δ_H 7.18-7.11 (m, 2H, ArH), 7.01-6.97 (m, 1H, ArH), 6.93-6.89 (m, 1H, ArH), 5.20 (q, J = 7.1, 1H, H2), 4.28 (qd, J = 6.5, 4.0, 1H, H5), 2.79-2.64 (m, 2H, H7), 2.58 (s, 3H, H3), 2.24-2.11 (m, 1H, H6a), 1.72-1.61 (m, 1H, H6b), 1.54 (d, J = 7.1, 3H, 1H), 1.18 (d, J = 6.5, 1H, H14). **^{13}C NMR** (101 MHz, $CDCl_3$): δ_C 159.5 (C4), 138.4 (Ar), 129.6 (Ar), 128.9 (ArH), 127.0 (ArH), 123.3 (ArH), 121.1 (ArH), 118.5 (CN), 50.3 (C5), 44.1 (C2), 32.8 (C3), 306 (C6), 24.6 (C7), 19.4 (C14), 16.9 (C1). **IR ν_{max}** (ATR)/cm^{-1}: 2953, 2870, 2852, 2263, 1642.

Diastereoisomer 2: ^{1}H NMR (400 MHz, $CDCl_3$): δ_H 7.18-7.11 (m, 2H, ArH), 7.01-6.97 (m, 1H, ArH), 6.93-6.89 (m, 1H, ArH), 5.21 (q, J = 7.4, 1H, H2), 4.35 (qd, J = 6.5, 4.5, 1H, H5), 2.79-2.64 (m, 2H, H7), 2.62 (s, 3H, H3), 2.24-2.11 (m, 1H, H6a), 1.72-1.61 (m, 1H, H6b), 1.51 (d, J = 7.4, 3H, H1), 1.19 (d, J = 6.5, 3H, H14). **^{13}C NMR** (101 MHz, $CDCl_3$): δ_C 158.2 (C4), 138.4 (Ar), 130.0 (Ar), 128.9 (ArH), 127.2 (ArH), 123.4 (ArH), 121.3 (ArH), 118.5 (CN), 50.3 (C5), 44.1 (C2), 32.2 (C3), 30.8 (C6), 24.8 (C7), 19.4 (C14), 17.4 (C1). **IR ν_{max}** (ATR)/cm^{-1}: 3080, 2970, 2870, 2242, 1642.

HRMS m/z (ESI$^+$): calcd. For $C_{15}H_{19}N_3ONa$ [M+Na] Calculated. 280.1528. Found. 280.1420.

1-(2,3,4,5-Tetrahydro-1*H*-benzo[*b*]azepine-1-carbonyl)pyrrolidine-2-carbonitrile (10a):

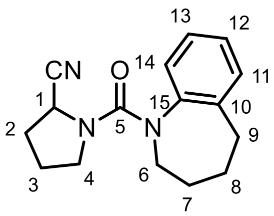

According to GP2, 2-cyanopyrrolidine-1-carbonyl chloride (181 mg, 1.15 mmol), 2,3,4,5-tetrahydro-1*H*-benzo[*b*]azepine (185 mg, 1.26 mmol) and triethylamine (0.32 mL, 2.29 mmol)

were refluxed at 75 °C for 45 h. Purification via flash column chromatography eluting with acetone:DCM (0:1 – 1:4) afforded the title compound (118 mg, 0.438 mmol, 38%) as a colourless solid. **R*f*** = 0.76 (1:9 Acetone:DCM). **^{1}H NMR** (400 MHz, $CDCl_3$): δ_H 7.21-7.10)(m, 3H, ArH), 7.06 (dd, J = 7.4, 1.5, 1H, ArH), 4.70-4.67 (m, br, 1H, H1), 4.52-3.92 (m, br, 1H, CH_2), 3.53-3.02 (m, br, 1H, CH_2), 2.79-2.74 (m, 2H, CH_2), 2.71 (s, br, 1H, CH_2), 2.52 (s, br, 1H, CH_2), 2.15-1.98 (m, 2H, CH_2), 1.91-1.67 (m, br, 5H, 3xCH_2), 1.64-1.50 (m, br, 1H, CH_2). **^{13}C NMR** (101 MHz, $CDCl_3$): δ_C 158.0 (C5), 144.1 (Ar), 139.2 (Ar), 130.4 (ArH), 127.6 (ArH), 126.6 (ArH), 125.9 (ArH), 119.6 (CN), 48.4 (C1), 47.8 (CH_2), 35.0 (CH_2), 30.4 (CH_2), 30.3 (CH_2), 28.7 (CH_2), 25.9 (CH_2), 25.2 (CH_2). **HRMS m/z** (ESI^+): calcd. For $C_{16}H_{19}N_3NaO$ [M+Na] Calculated. 292.1420. Found. 292.1423. **IR ν_{max}** (ATR)/cm^{-1}: 2980, 2932, 2940, 2248, 1639. **m.p**. 131-134 °C.

1-(1,2,3,4,5,6-Hexahydrobenzo[*b*]azocine-1-carbonyl)pyrrolidine-2-carbonitrile (10b):

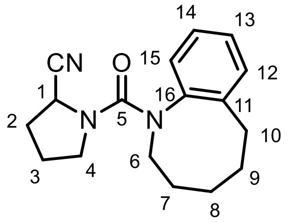

According to GP2, 2-cyanopyrrolidine-1-carbonyl chloride (89 mg, 0.564 mmol), 1,2,3,4,5,6-hexahydrobenzo[*b*]azocine (100 mg, 0.620 mmol) and triethylamine (0.16 mL, 1.13 mmol) were refluxed at 75 °C for 17 h. Purification via flash column chromatography eluting with acetone:DCM (0:1 – 1:4) afforded the title compound (129 mg, 0.456 mmol, 81%) as a yellow oil. **R*f*** = 0.63 (1:9 Acetone:DCM). **^{1}H NMR** (400 MHz, $CDCl_3$): δ_H 7.30-7.20 (m, 3H, ArH), 7.12-7.08 (m, 1H, ArH), 4.72 (t, J = 6.0, 1H, H1), 4.45-3.00 (m, br, 2H, CH_2), 2.78-2.64 (m, br, 3H, 2xCH_2), 2.09-1.98 (m, 2H, CH_2), 1.95-1.84 (m, 1H, CH_2), 1.82-1.73 (m, br, 2H, CH_2), 1.69-1.38 (m, br, 5H, 3xCH_2). **^{13}C NMR** (101 MHz, $CDCl_3$): δ_C 159.1 (C5), 142.5 (Ar), 141.6 (Ar), 130.3 (ArH), 128.5 (ArH), 128.3 (ArH), 127.8 (ArH), 119.7 (CN), 53.8 (CH_2), 49.0 (C1), 47.6 (CH_2), 31.7

(CH_2), 31.5 (CH_2), 30.0 (CH_2), 26.6 (CH_2), 26.2 (CH_2), 25.4 (CH_2). **HRMS m/z** (ESI^+): calcd. For $C_{17}H_{21}N_3ONa$ [M+Na] Calculated. 306.1577. Found. 306.1578. **IR ν_{max}** (ATR)/cm^{-1}: 2924, 2854, 2242, 1633.

1-(1,2,3,4-Tetrahydroquinoline-1-carbonyl)-piperidine-2-carbonitrile (910):

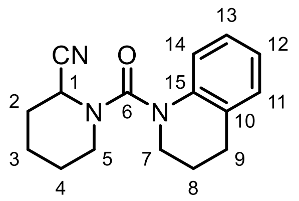

According to GP2, 2-cyanopiperidine-1-carbonyl chloride (118 mg, 0.683 mmol), 1,2,3,4-tetrahydroquinoline (0.10 mL, 0.751 mmol) and triethylamine (0.19 mL, 1.37 mmol) were refluxed at 75 °C for 48 h. Purification via flash column chromatography eluting with EtOAc:PE (1:9 – 7:3) afforded the title compound (75 mg, 0.278 mmol, 41%) as an orange solid. **R_f** = 0.34 (1:3 EtOAc:PE). **^{1}H NMR** (400 MHz, $CDCl_3$): δ_H 7.15-7.08 (m, 3H, ArH), 6.97 (td, J = 7.2, 1.5, 1H, ArH), 5.13 (m, 1H, H1), 3.68 (dt, J = 12.6, 6.5, 1H, H7a), 3.58 (dt, J = 12.6, 6.5, 1H, H7b), 3.41 (d, br, J = 13.6, 1H, H2a), 2.97-2.89 (m, 1H, H2b), 2.75 (t, J = 6.5, 2H, H9), 2.00-1.91 (m, 3H, H5a/8), 1.83-1.63 (m, 3H, H4/5b), 1.53 (d, br, J = 13.5, 1H, H3a), 1.35 (qt, J = 13.5, 3.4, 1H, H3b). **^{13}C NMR** (101 MHz, $CDCl_3$): δ_C 159.8 (C6), 140.0 (Ar), 129.2 (ArH), 129.1 (Ar), 126.8 (ArH), 123.4 (ArH), 120.4 (ArH), 117.8 (CN), 45.7 (C1), 45.6 (C7), 44.8 (C2), 28.5 (C5), 27.0 (C9), 24.5 (C3), 23.9 (C8), 20.6 (C4). **HRMS m/z** (ESI^+): calcd. For $C_{16}H_{20}N_3O$ [M+H] Calculated. 270.1528. Found. 270.1613. **IR ν_{max}** (ATR)/cm^{-1}: 2948, 2865, 2233, 1652. **m.p.** 138-140 °C.

***tert*-Butyl 4-(2-cyanopiperidine-1-carbonyl)-3,4-dihydroquinoxaline-1(2*H*)-carbonxylate (10d):**

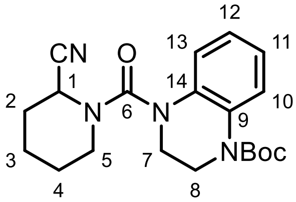

According to GP2, 2-cyanopiperidine-1-carbonyl chloride (201 mg, 1.16 mmol), *tert*-butyl-3,4-dihydroquinoxaline-1(2*H*)-carboxylate (295 mg, 1.28 mmol) and triethylamine (0.32 mL, 2.33 mmol) were refluxed at 75 °C for 21 h. Purification via flash column chromatography eluting with EtOAc:PE (5:95 – 2:3) afforded the title compound (253 mg, 0.684 mmol, 59%) as an orange oil. **R*f*** = 0.29 (1:4 EtOAc:PE). **^{1}H NMR** (400 MHz, $CDCl_3$): δ_H 7.89 (d, J = 7.7, 1H, ArH), 7.09-6.99 (m, 3H, ArH), 5.22-5.17 (m, 1H, H1), 3.87-3.83 (m, 2H, H8), 3.82-3.78 (m, 1H, H7a), 3.70-3.62 (m, 1H, H7b), 3.54-3.51 (m, br, 1H, CH_2), 2.96 (td, J = 13.2, 2.8, 1H, CH_2), 1.99-1.95 (m, 1H, CH_2), 1.84-1.68 (m, 3H, CH_2), 1.61-1.57 (m, 1H, CH_2), 1.54 (s, 9H, Boc-CH_3), 1.42-1.30 (m, 1H, CH_2). **^{13}C NMR** (101 MHz, $CDCl_3$): δ_C 158.4 (C6), 153.4 (Boc-CO), 133.4 (Ar), 130.47 (Ar), 124.3 (2xArH), 123.9 (ArH), 119.6 (ArH), 117.6 (CN), 81.8 (Boc-C(Me)$_3$), 47.4 (C8), 45.7 (C1), 45.2 (C7), 45.1 (CH_2), 28.5 (CH_2/Boc-CH_3), 24.4 (CH_2), 20.6 (CH_2). **HRMS m/z** (ESI^+): calcd. For $C_{20}H_{27}N_4O_3$ [M+H] Calculated. 371.2078. Found. 371.2077. **IR ν_{max}** (ATR)/cm^{-1}: 2978, 2940, 2862, 2238, 1699, 1651.

1-(3,4-Dihydro-2*H*-benzo[*b*][1,4]thiazine-4-carbonyl)piperidine-2-carbonitrile (10e):

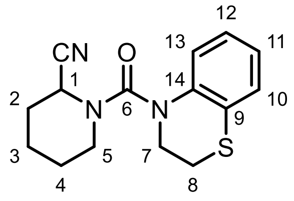

According to GP2, 2-cyanopiperidine-1-carbonyl chloride (214 mg, 1.24 mmol), 3,4-dihydro-2*H*-1,4-benzothiazine (200 mg, 1.37 mmol) and triethylamine (0.35 mL, 2.48 mmol) were

refluxed at 75 °C for 21 h. Purification via flash column chromatography eluting with EtOAc:PE (5:95 – 2:3) afforded the title compound (252 mg, 0.878 mmol, 71%) as an orange oil. **R*f*** = 0.29 (1:4 EtOAc:PE). **^{1}H NMR** (400 MHz, $CDCl_3$): δ_H 7.23 (ddd, J = 7.7, 1.6, 0.6, 1H, ArH), 7.10-6.99 (m, 3H, ArH), 5.14-5.09 (m, 1H, H1), 3.99 (ddd, J = 13.3, 6.4, 5.4, 1H, H7 or 8), 3.82 (ddd, J = 13.3, 6.5, 5.4, 1H, H7 or 8), 3.30 (dddd, J = 13.6, 5.8, 2.6, 1.3, 1H, CH_2), 3.20 (ddd, J = 6.4, 5.4, 1.1, 2H, H7 or 8), 2.84 (ddd, J = 13.6, 12.7, 2.8, 1H, CH_2), 1.97-1.91 (m, 1H, CH_2), 1.79-1.59 (m, 3H, CH_2), 1.50-1.44 (m, 1H, CH_2), 1.31-1.19 (m, 1H, CH_2). **^{13}C NMR** (101 MHz, $CDCl_3$): δ_C 158.9 (C6), 139.4 (Ar), 127.9 (ArH), 126.9 (Ar), 125.4 (ArH), 124.8 (ArH), 122.3 (ArH), 117.4 (CN), 45.7 (C1), 44.7 (C 7 or 8), 44.0 (C7 or 8). 28.3 (CH_2), 24.2 (CH_2), 20.4 (CH_2). **HRMS m/z** (ESI$^+$): calcd. For $C_{15}H_{17}N_3OSNa$ [M+Na] Calculated. 310.0985. Found. 310.0988. **IR ν$_{max}$** (ATR)/cm^{-1}: 2944, 2853, 2238, 1646.

1-(2,3,4,5-Tetrahydro-1*H*-benzo[*b*]azepine-1-carbonyl)piperidine-2-carbonitrile (10f):

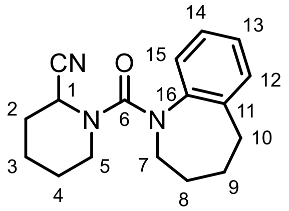

According to GP2, 2-cyanopiperidine-1-carbonyl chloride (101 mg, 0.587 mmol), 2,3,4,5-tetrahydro-1*H*-benzo[*b*]azepine (95 mg, 0.645 mmol) and triethylamine (0.16 mL, 1.17 mmol) were refluxed at 75 °C for 21 h. Purification via flash column chromatography eluting with EtOAc:PE (5:95 – 2:3) afforded the title compound (88 mg, 0311 mmol, 53%) as an orange oil. **R*f*** = 0.30 (1:4 EtOAc:PE). **^{1}H NMR** (400 MHz, $CDCl_3$): δ_H 7.24-7.11 (m, 3H, ArH), 6.99 (dd, J = 7.7, 1.5, 1H, ArH), 5.06 (s, 1H, H1), 4.06-3.36 (m, br, 2H, H7), 3.17-3.13 (m, 1H, CH_2), 2.79-2.76 (m, 2H, H9), 2.68 (t, J = 12.4, 1H, CH_2), 1.89-1.52 (m, 8H, CH_2), 1.33 (d, br, J = 14.0, 1H, CH_2), 1.08-0.95 (m, 1H, CH_2). **^{13}C NMR** (101 MHz, $CDCl_3$): δ_C 160.4 (C6), 145.2 (Ar), 138.5 (Ar), 131.0

(ArH), 127.9 (ArH), 127.0 (ArH), 125.5 (ArH), 118.2 (CN), 49.1 (C1), 46.1 (C7), 44.4 (CH_2), 35.5 (C9), 30.8 (CH_2), 28.7 (CH_2), 26.1 (CH), 24.4 (CH_2), 21.0 (CH_2). **HRMS m/z** (ESI^+): calcd. For $C_{17}H_{22}N_3O$ [M+H] Calculated. 284.1757. Found. 284.1757. **IR ν_{max}** (ATR)/cm^{-1}: 2928, 2856, 2235, 1645.

1-(1,2,3,4,5,6-Hexahydrobenzo[*b*]azocine-1-carbonyl)piperidine-2-carbonitrile (10g):

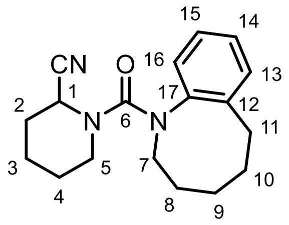

According to GP2, 2-cyanopiperidine-1-carbonyl chloride (97 mg, 0.564 mmol), 1,2,3,4,5,6-hexahydrobenzo[*b*]azocine (100 mg, 0.620 mmol) and triethylamine (0.16 mL, 1.13 mmol) were refluxed at 75 °C for 48 h. Purification via flash column chromatography eluting with EtOAc:PE (0:1 – 1:1) afforded the title compound (129 mg, 0.434 mmol, 77%) as a yellow oil. **R_f** = 0.59 (1:3 EtOAc:PE). **^{1}H NMR** (400 MHz, $CDCl_3$): δ_H 7.30-7.25 (m, 3H, ArH), 7.04-7.02 (m, 1H, ArH), 5.05 (s, br, 1H, H1), 3.62 (s, br, 2H, H7), 3.48 (d, br, J = 14.8, 1H, H2a), 2.77-2.62 (m, 3H, H2b/CH_2), 1.82-1.49 (m, 10H, 5 x CH_2), 1.31 (d, br, J = 13.9, 1H, H3a), 0.88-0.73 (m, 1H, H3b). **^{13}C NMR** (101 MHz, $CDCl_3$): δ_C 161.8 (C6), 142.4 (Ar), 141.4 (Ar), 130.5 (ArH), 128.0 (ArH), 127.8 (ArH), 127.5 (ArH), 117.9 (CN), 54.0 (C7), 45.9 (C1), 44.0 (C2), 31.6 (CH_2), 31.5 (CH_2), 28.4 (CH_2), 26.6 (CH_2), 25.9 (CH_2), 24.0 (C3), 20.5 (CH_2). **HRMS m/z** (ESI^+): calcd. For $C_{18}H_{24}N_3O$ [M+H] Calculated. 298.1841. Found. 298.1923. **IR ν_{max}** (ATR)/cm^{-1}: 2924, 2861, 2242, 1645.

***N*-(1-cyanoethyl)-*N*-methyl5-oxo-2,3,4,5-tetrahydro-1*H*-benzo[*b*]azepine-1-carboxamide (12):**

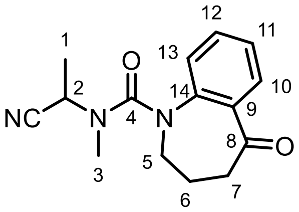

According to GP2, (1-cyanoethyl)(methyl)carbamic chloride (200 mg, 1.37 mmol), 1,2,3,4-tetrahydro-5*H*-benzo[*b*]azepine-5-one (200 mg, 1.34 mmol) and triethylamine (0.38 mL, 2.73 mmol) were refluxed at 75 °C for 75 h. Purification via flash column chromatography eluting with EtOAc:PE (3:7 – 3:2) afforded the title compound (267 mg, 0.985 mmol, 79%) as a white solid. $\mathbf{R_f}$ = 0.51 (1:1 EtOAc:PE). **^{1}H NMR** (400 MHz, $CDCl_3$): δ_H 7.81 (dd, J = 7.8, 1.7, 1H, ArH), 7.52 (td, J = 7.8, 1.7, 1H, ArH), 7.33 (td, J = 7.8, 1.1, 1H, ArH), 7.01 (dd, J = 7.8, 1.1, 1H, ArH), 5.25 (q, J = 7.2, 1H, H2), 3.78 (dt, J = 13.5, 6.8, 1H, H5a), 3.70 (dt, J = 13.5, 6.7, 1H, H5b), 2.76-2.73 (m, 2H, H7), 2.39 (s, 3H, H3), 2.07-1.96 (m, 2H, H6), 1.45 (d, J = 7.2, 3H, H1). **^{13}C NMR** (101 MHz, $CDCl_3$): δ_C 203.4 (C8), 160.9 (C4), 143.4 (Ar), 134.7 (Ar), 130.3 (ArH), 127.2 (ArH), 125.5 (ArH), 118.6 (CN), 49.6 (C5), 44.4 (C2), 40.3 (C7), 32.6 (C3), 23.1 (C6), 17.4 (C1). **HRMS m/z** (ESI$^+$): calcd. For $C_{15}H_{18}N_3O_2$ [M+H] Calculated. 272.1321. Found. 272.1394. **IR ν_{max}** (ATR)/cm^{-1}: 3080, 2967, 2885, 2239, 1675, 1646. **m.p.** 114-117 °C.

***tert*-Butyl 4-((1-cyanoethyl)(methyl)carbamoyl)-3,4-dihydroquinoxaline-1(2*H*)-carboxylate (14):**

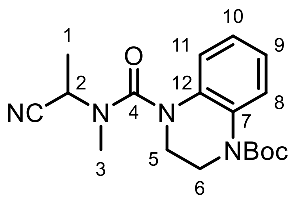

According to GP2, (1-cyanoethyl)(methyl)carbamic chloride (59 mg, 0.400 mmol), *tert*-butyl-3,4-dihydroquinoxaline-1(2*H*)-carboxylate (103 mg, 0.555 mmol) and triethylamine (0.11 mL, 0.800 mmol) were refluxed at 75 °C for 27 h. Purification via flash column chromatography

eluting with EtOAc:PE (2:3 – 9:11) afforded the title compound (108 mg, 0.314 mmol, 78%) as a yellow oil. **R*f*** = 0.20 (1:3 EtOAc:PE). **^{1}H NMR** (400 MHz, $CDCl_3$): δ_H 7.87 (d, br, J = 8.1, 1H, ArH), 7.06 (td, J = 8.1, 1.8, 1H, ArH), 7.01 (td, J = 7.7, 1.8, 1H, ArH), 6.93 (dd, J = 7.7, 1.7, 1H, ArH), 5.226 (q, J = 7.3, 1H, H2), 3.89-3.82 (m, 2H, H6), 3.82-3.77 (m, 1H, H5a), 3.65 (dt, J = 12.6, 6.1, 1H, H5b), 2.74 (s, 3H, H3), 1.59-1.53 (m, 12H, H1/Boc-CH_3). **^{13}C NMR** (101 MHz, $CDCl_3$): δ_C 158.6 (C4), 153.3 (Boc-CO), 133.4 (Ar), 131.0 (Ar), 124.4 (ArH), 124.1 (ArH), 124.0 (ArH), 119.6 (ArH), 118.4 (CN), 81.8 (Boc-C(Me)$_3$), 47.4 (C6), 45.4 (C5), 44.2 (C2), 33.1 (C3), 28.5 (Boc-CH_3), 17.2 (C1). **HRMS m/z** (ESI$^+$): calcd. For $C_{18}H_{24}N_4O_3$ [M+Na] Calculated. 367.1848. Found. 367.1731. **IR ν_{max}** (ATR)/cm^{-1}: 2958, 2923, 2852, 2239, 1706, 1652.

***N*[1],*N*[4]-bis(1-cyanoethyl)-*N*[1],*N*[4]-dimethyl-2,3-dihydroquinoxaline-1,4-dicarboxamide (17):**

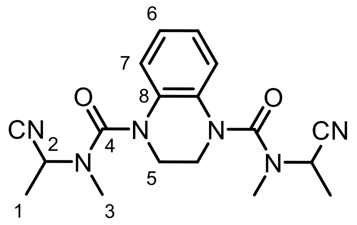

According to GP2, (1-cyanoethyl)(methyl)carbamic chloride (1.35 g, 9.21 mmol), 1,2,3,4-tetrahydroquinoxaline (450 mg, 3.35 mmol) and triethylamine (2.56 mL, 18.4 mmol) were refluxed at 75 °C for 60 h. Purification via flash column chromatography eluting with EtOAc:PE (1:4 – 1:0) afforded the title compound (1.01 g, 2.85 mmol, 85%) as a pink solid. **R*f*** = 0.63 (1:0 EtOAc:PE). **^{1}H NMR** (400 MHz, $CDCl_3$): δ_H 7.09-7.01 (m, 4H, ArH), 5.33-5.24 (m, 2H, H2), 3.87-3.74 (m, 4H, H5), 2.73 (s, 3H, H3), 2.72 (s, 3H, H3), 1.58 (d, J = 7.3, 3H, H1), 1.58 (D, J = 7.3, 3H, H1). **^{13}C NMR** (101 MHz, $CDCl_3$): δ_C 159.9 (C4), 132.8 (Ar), 124.7 (ArH), 120.9 (ArH), 118.3 (CN), 46.9 (C5), 44.2 (C2), 32.9 (C3), 17.1 (C1). **HRMS m/z** (ESI$^+$): calcd. For $C_{18}H_{22}N_6O_2Na$ [M+Na] Calculated 377.1804. Found. 377.1707. **IR ν_{max}** (ATR)/cm^{-1}: 2971, 2880, 2244, 1652. **m.p.** 166-168 °C.

2.3. Ring Expansion Products

12-Imino-1,2-dimethyl-1,5,6,7-tetrahydro-1,4-methanobenzo[*e*][1,3]diazonin-3(2*H*)-one (2a):

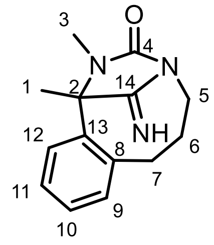

According to GP3, *N*-(1-cyanoethyl)-*N*-methyl-3,4-dihydroquinoline-1(2*H*)-carboxamide (102 mg, 0.419 mmol) and KHMDS (1M in THF, 0.82 mL, 0.822 mmol) in anhydrous THF stirred at 0 °C for 30 min. Purification via flash column chromatography eluting with EtOAc:PE (7:3 – 4:1) afforded the title compound (74 mg, 0.304 mmol, 73%) as a yellow solid. **R_f** = 0.15 (3:1 EtOAc:PE). **^{1}H NMR** (400 MHz, $CDCl_3$): δ_H 7.39-7.37 (m, 1H, ArH), 7.24-7.19 (m, 2H, ArH), 7.12-7.09 (m, 1H, ArH), 3.88 (ddd, J = 13.7, 12.0, 5.8, 1H, H5a), 3.69-3.58 (m, br, 1H, H5b), 2.83-2.72 (m, br, 1H, H7a), 2.66 (s, 3H, H3), 2.53 (dd, J = 16.3, 9.2, 1H, H7b), 2.27-2.15 (m, br, 1H, H6a), 1.95 (ddd, J = 15.0, 10.1, 5.8, 1H, H6b), 1.87 (s, 3H, H1). **^{13}C NMR** (101 MHz, $CDCl_3$): δ_C 172.0 (C14), 161.2 (C4), 140.0 (Ar), 138.4 (Ar), 135.3 (ArH), 128.9 (ArH), 126.5 (ArH), 126.4 (ArH), 65.2 (C2, br), 42.1 (C5, br), 31.5 (C7, br), 25.3 (C3), 24.6 (C6, br), 20.2 (C1, br). **HRMS m/z** (ESI$^+$): calcd. For $C_{14}H_{17}N_3ONa$ [M+Na] Calculated. 266.1269. Found. 266.1276. **IR ν_{max}** (ATR)/cm^{-1}: 3210, 2996, 2954, 2923, 2887, 1732, 1668. **m.p.** 129-133 °C.

13-Imino-1,2-dimethyl-1,2,5,6,7,8-hexahydro-3*H*-1,4-methanobenzo[*e*][1,3]diazecine-3-one (4):

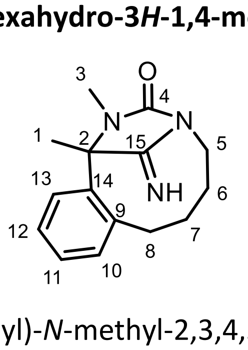

According to GP3, *N*-(1-cyanoethyl)-*N*-methyl-2,3,4,5-tetrahydro-1*H*-benzo[*b*]azepine-1-carboxamide (63 mg, 0.245 mmol) and KHMDS (1M in THF, 0.47 mL, 0.466 mmol) in

anhydrous THF stirred at 0 °C for 1.5 h. The title compound (57 mg, 0.221 mmol, 91%) was obtained as a yellow solid without any further purification. **R*f*** = 0.12 (7:3 EtOAc:PE). **^{1}H NMR** (400 MHz, $CDCl_3$): δ_H 7.43-7.41 (m, 1H, ArH), 7.24-7.22 (m, 2H, ArH), 7.09-7.07 (m, 1H, ArH), 4.03 (ddd, J = 13.9, 12.3, 5.7 Hz, 1H, H5a), 3.80 (dd, J = 13.9, 6.0, 1H, H5b), 2.79 (ddd, J = 15.4, 12.7, 6.7, 1H, H8a), 2.71 (s, 3H, H3), 2.43 (ddd, J = 15.4, 6.4, 1.8, 1H, H8b), 2.10-1.98 (m, 1H, H6a), 1.92-1.74 (m, 2H, H7a/H6b), 1.83 (s, 3H, H1), 1.62-1.53 (m, 1H, H7b). **^{13}C NMR** (101 MHz, $CDCl_3$): δ_C 172.1 (C15), 158.6 (C4), 142.3 (Ar), 136.2 (Ar), 133.9 (ArH), 129.2 (ArH), 127.4 (ArH), 126.5 (ArH), 66.7 (C2), 39.9 (C5), 33.9 (C8), 28.7 (C6), 25.4 (C3), 24.0 (C7), 22.7 (C1). **HRMS m/z** (ESI^+): calcd. For $C_{15}H_{20}N_3O$ [M+H] Calculated. 258.1528. Found. 258.1612. **IR ν_{max}** (ATR)/cm^{-1}: 3275, 2953, 2921, 2897, 2858, 1721, 1651. **m.p.** 170-173 °C.

14-Imino-1,2-dimethyl-1,5,6,7,8,9-hexahydro-1,4-methanobenzo[*e*][1,3]diazacycloundecin-3(2*H*)-one (6):

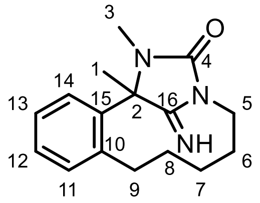

According to GP3, *N*-(1-cyanoethyl)-*N*-methyl-3,4,5,6-tetrahydrobenzo[*b*]azocine-1(2*H*)-carboxamide (50 mg, 0.184 mmol) and KHMDS (1M in THF, 0.37 mL, 0.369 mmol) in anhydrous THF stirred at 0 °C for 1 h. The title compound (45 mg, 0.166 mmol, 90%) was obtained as a yellow oil without any further purification. **R*f*** = 0.18 (7:3 EtOAc:PE). **^{1}H NMR** (400 MHz, $CDCl_3$): δ_H 7.45 (dd, J = 7.5, 1.6, 1H, ArH), 7.29-7.21 (m, 2H, ArH), 7.13 (dd, J = 7.1, 1.9, 1H, ArH), 3.78 (m, 2H, H5), 2.64 (s, 3H, H3), 2.48-2.38 (m, br, 1H, H9a), 2.38-2.32 (m, br, 1H, H9b), 2.26-2.12 (m, br, 1H, H6a), 1.90-1.85 (m, br, 1H, H6b), 1.82 (s, 3H, H1), 1.71-1.59 (m, br, 3H, H7a/8), 1.52-1.42 (m, br, 1H, H7b). **^{13}C NMR** (101 MHz, $CDCl_3$): δ_C 169.7 (C16),

157.0 (C4), 143.3 (Ar), 134.8 (Ar), 133.2 (ArH), 129.3 (ArH), 128.1 (ArH), 126.2 (ArH), 65.5 (C2), 39.8 (C5), 31.2 (C9), 28.6 (C8), 25.5 (C7), 25.2 (C3), 24.0 (C1), 23.5 (C6). **HRMS m/z** (ESI$^+$): calcd. For $C_{16}H_{22}N_3O$ [M+H] Calculated. 272.1685. Found. 272.1754. **IR ν_{max}** (ATR)/cm^{-1}: 3281, 2925, 2852, 1727, 1650.

10-Chloro-12-imino-1,2-dimethyl-1,5,6,7-tetrahydro-1,4-methanobenzo[*e*][1,3]diazonin-3(2*H*)-one (2b):

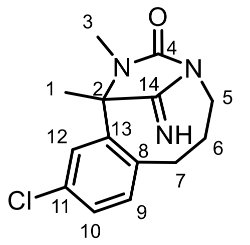

According to GP3, 7-chloro-*N*-(1-cyanoethyl)-*N*-methyl-3,4-dihydroquinoline-1(2*H*)-carboxamide (62 mg, 0.224 mmol) and KHMDS (1M in THF, 0.45 mL, 0.448 mmol) in anhydrous THF stirred at 0 °C for 1 h. Purification via flash column chromatography eluting with EtOAc:PE (1:9 – 1:0) afforded the title compound (43 mg, 0.0155 mmol, 69%) as a colourless solid. **R*f*** = 0.24 (7:3 EtOAc:PE). **^{1}H NMR** (400 MHz, $CDCl_3$): δ_H 7.36 (d, J = 2.3, 1H, ArH), 7.20 (dd, J = 8.2, 2.3, 1H, ArH), 7.05 (d, J = 8.2, 1H, ArH), 3.88 (ddd, J = 13.9, 12.0, 5.8, 1H, H5a), 3.63 (dd, J = 13.9, 6.3, 1H, H5b), 2.83-2.72 (m, br, 1H, H7a), 2.68 (s, 3H, H3), 2.49 (dd, J = 16.3, 9.1, 1H, H7b), 2.26-2.10 (m, br, 1H, H6a), 1.96 (dddt, J = 14.9, 10.1, 5.7, 1.0, 1H, H6b), 1.88 (s, 3H, H1). **^{13}C NMR** (101 MHz, $CDCl_3$): δ_C 171.4 (C4), 161.3 (C14), 140.6 (Ar), 138.8 (Ar), 136.9 (ArH), 132.7 (Ar), 129.0 (ArH), 127.1 (ArH), 65.7 (C2), 42.2 (C5), 31.2 (C7), 25. (C3), 24.8 (C6), 20.5 (C1). **HRMS m/z** (ESI$^+$): calcd. For $C_{14}H_{17}ClN_3$ [M+H] Calculated. 278.1055. Found. 278.1060. **IR ν_{max}** (ATR)/cm^{-1}: 3263, 2957, 2850, 1732, 1662. **m.p.** 155-157 °C.

10-Bromo-12-imino-1,2-dimethyl-1,5,6,7-tetrahydro-1,4-methanobenzo[*e*][1,3]diazonin-3(2*H*)-one (2c):

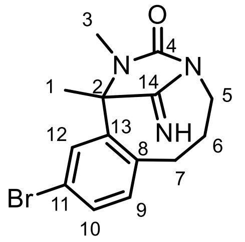

According to GP3, 7-bromo-*N*-(1-cyanoethyl)-*N*-methyl-3,4-dihydroquinoline-1(2*H*)-carboxamide (25 mg, 0.0779 mmol) and KHMDS (1M in THF, 0.19 mL, 0.187 mmol) in anhydrous THF stirred at 0 °C for 1 h. Purification via flash column chromatography eluting with EtOAc:PE (1:9 – 1:0) afforded the title compound (12 mg, 0.0380 mmol, 49%) as a white solid. **R*f*** = 0.45 (1:0 EtOAc:PE). **^{1}H NMR** (400 MHz, $CDCl_3$): δ_H 7.56 (s, br, 1H, NH), 7.50 (s, br, 1H, ArH), 7.35 (dd, J = 8.2, 2.2, 1H, ArH), 6.99 (d, J = 8.2, 1H, ArH), 3.93-3.85 (m, 1H, H5a), 3.75-3.49 (m, br, 1H, H5b), 2.84-2.70 (m, br, 1H, H7a), 2.69 (s, 3H, H3), 2.49 (dd, J = 16.3, 9.1, 1H, H7b), 2.26-2.11 (m, br, 1H, H6a), 1.96 (ddd, J = 15.3, 10.1, 5.8, 1H, H6b), 1.88 (s, br, 3H, H1). **^{13}C NMR** (101 MHz, $CDCl_3$): δ_C 171.9 (C4), 161.3 (C14), 140.9 (Ar), 139.3 (Ar), 137.0 (ArH), 132.0 (ArH), 129.8 (ArH), 120.8 (Ar), 65.5 (C2), 42.1 (C5), 31.1 (C7), 25.6 (C3), 24.6 (C6), 20.5 (C1). **HRMS m/z** (ESI^+): calcd. For $C_{14}H_{17}BrN_3O$ [M+H] Calculated. 322.0550. Found. 322.0560. **IR ν_{max}** (ATR)/cm^{-1}: 3265, 2919, 2858, 1723, 1659. **m.p.** 175-178 °C.

12-Imino-1,2,9-trimethyl-1,5,6,7-tetrahydro-1,4-methanobenzo[*e*][1,3]diazonin-3(2*H*)-one (2d):

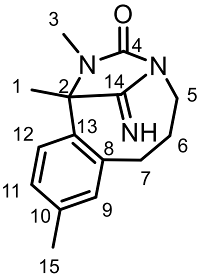

According to GP3, *N*-(1-cyanoethyl)-*N*,6-dimethyl-3,4-dihydroquinoline-1(2*H*)-carboxamide (100 mg, 0.389 mmol) and KHMDS (1M in THF, 0.78 mL, 0.778 mmol) in anhydrous THF stirred

at 0 °C for 2 h. Purification via flash column chromatography eluting with MeOH:DCM (0:1 – 1:9) afforded the title compound (35 mg, 0.136 mmol, 35%) as a yellow gum. **R*f*** = 0.41 (5:95 MeOH:DCM). **^{1}H NMR** (400 MHz, $CDCl_3$): δ_H 7.51 (s, br, 1H, NH), 7.27 (d, J = 7.8, 1H, ArH), 7.04 (dd, J = 7.8, 1.5, 1H, ArH), 6.93 (d, J = 1.5, 1H, ArH), 3.89 (ddd, J = 13.7, 12.0, 5.7, 1H, H5a), 3.67-3.60 (m, 1H, H5b), 2.80-2.69 (m, 1H, H7a), 2.67 (s, 3H, H3), 2.50 (dd, J = 16.2, 9.1, 1H, H7b), 2.28 (s, 3H, H15), 2.23-2.19 (m, 1H, H6a), 1.99-1.91 (m, 1H, H6b), 1.86 (s, 3H, H1). **^{13}C NMR** (101 MHz, $CDCl_3$): δ_C 173.0 (C4), 161.6 (C14), 140.3 (Ar), 139.4 (Ar), 136.9 (ArH), 135.9 (Ar), 127.6 (ArH), 127.1 (ArH), 66.1 (C2), 42.8 (C5), 31.9 (C7), 25.9 (C3), 25.2 (C6), 21.2 (C15), 20.7 (C1). **HRMS m/z** (ESI$^+$): calcd. For $C_{15}H_{20}N_3O$ [M+H] Calculated. 258.1528. Found. 258.1607. **IR ν_{max}** (ATR)/cm^{-1}: 3233, 2923, 2852, 1726, 1664.

12-Imino-6,7-dimethyl-2,3,6,7-tetrahydro-5*H*-4,7-methanobenzo[*h*][1,4,6]thiadiazonin-5-one (2e):

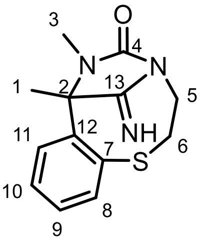

According to GP3, *N*-(1-cyanoethyl)-*N*-methyl-2,3-dihydro-4*H*-benzo[*b*][1,4]thiazine-4-carboxamide (50 mg, 0.192 mmol) and KHMDS (1M in THF, 0.38 mL, 0.384 mmol) in anhydrous THF stirred at 0 °C for 1 h. Purification via flash column chromatography eluting with MeOH:DCM (0:1 – 1:9) afforded the title compound (27 mg, 0.103 mmol, 54%) as a yellow oil. **R*f*** = 0.68 (1:9 MeOH:DCM). **^{1}H NMR** (400 MHz, $CDCl_3$): δ_H 7.70-7.68 (m, 1H, ArH), 7.50 (d, br, J = 7.7, 1H, ArH), 7.40 (td, J = 8.9, 7.7, 1H, ArH), 7.28-7.25 (m, 1H, ArH), 4.00 (td, J = 13.1, 5.0, 1H, CH_2), 3.87-3.83 (m, br, 1H, CH_2), 3.56-3.42 (m, br, 1H, CH_2), 3.03 (dd, J = 14.8, 5.0, 1H, CH_2), 2.64 (s, 3H, H3), 1.91 (s, 3H, H1). **^{13}C NMR** (125 MHz, $CDCl_3$): δ_C 172.8 (C13), 160.9 (C4), 143.1 (Ar), 141.4 (ArH), 133.1 (Ar), 129.6 (ArH), 129.4 (ArH), 127.6 (ArH), 65.7 (C2),

42.9 (CH_2), 34.4 (CH_2), 26.3 (C3), 21.4 (C1). **HRMS m/z** (ESI^+): calcd. For $C_{13}H_{16}N_3OS$ [M+H] Calculated. 262.0936. Found. 262.1000. **IR ν_{max}** (ATR)/cm^{-1}: 3256, 3059, 2934, 2857, 1733, 1660.

12-Imino-6-dimethyl-2,3,6,7-tetrahydro-5*H*-4,7-methanobenzo[*h*][1,4,6]oxadiazonin-5-one (2f):

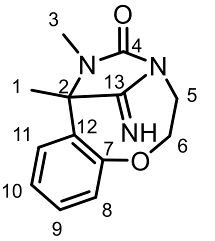

According to GP3, *N*-(1-cyanoethyl)-*N*-methyl-2,3-dihydro-4*H*-benzo[*b*][1,4]oxazine-4-carboxamide (55 mg, 0.224 mmol) and KHMDS (1M in THF, 0.45 mL, 0.448 mmol) in anhydrous THF stirred at 0 °C for 1 h. Purification via flash column chromatography eluting with EtOAc:PE (1:3 – 1:0) afforded the title compound (16 mg, 0.0652 mmol, 29%) as a yellow oil. R_f = 0.15 (3:1 EtOAc:PE). **^{1}H NMR** (400 MHz, $CDCl_3$): δ_H 7.78 (s, br, 1H, NH), 7.35 (d, J = 7.7, 1H, ArH), 7.31 (td, J = 7.8, 1.7, 1H, ArH), 7.16 (td, J = 7.7, 1.4, 1H, ArH), 7.06 (dd, J = 7.8, 1.4, 1H, ArH), 4.40 (dd, J = 11.1, 5.9, 1H, H6a), 4.25 (ddd, J = 13.7, 11.9, 5.9, 1H, H5a), 4.00-3.87 (m, br, 1H, H6b), 3.55-2.75 (m, br, 1H, H5b), 2.62 (s, br, 3H, H3), 1.86 (s, br, 3H, H1). **^{13}C NMR** (101 MHz, $CDCl_3$): δ_C 173.9 (C13), 162.6 (C4), 159.0 (C7), 133.4 (C12), 130.7 (ArH), 126.9 (ArH), 125.7 (ArH), 124.8 (ArH), 69.2 (C6), 65.2 (C2), 44.4 (C5), 25.8 (C3), 19.1 (C1). **HRMS m/z** (ESI^+): calcd. For $C_{13}H_{16}N_3O_2$ [M+H] Calculated. 246.1164. Found. 246.1258. **IR ν_{max}** (ATR)/cm^{-1}: 3555, 3349, 3260, 3204, 2966, 2926, 1740, 1667.

12-Imino-1,2,5-trimethyl-1,5,6,7-tetrahydro-1,4-methanobenzo[*e*][1,3]diazonin-3(2H)-one (2g & 2g'):

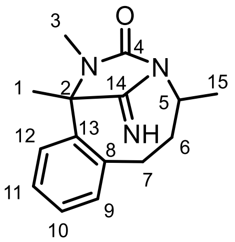

According to GP3, *N*-(1-cyanoethyl)-*N*,2-dimethyl-3,4-dihydroquinoline-1(2*H*)-carboxamide (53 mg, 0.206 mmol) and KHMDS (1M in THF, 0.41 mL, 0.412 mmol) in anhydrous THF stirred at 0 °C for 2 h. Purification via column chromatography eluting with EtOAc:PE (1:4 – 1:0) afforded the title compound (35 mg, 0.136 mmol, 66%) as a mixture of 2 isolated diastereoisomers. **dr** = 2.1:1.0.

Major diastereoisomer (2g): (24 mg, 0.0933 mmol, 45%) as a white solid. **R*f*** = 0.33 (3:1 EtOAc:PE). **^{1}H NMR** (400 MHz, $CDCl_3$): δ_H 7.90 (s, br, 1H, NH), 7.34 (d, br, J = 7.5, 1H, ArH), 7.25 (td, J = 7.5, 1.7, 1H, ArH), 7.20 (td, J = 7.5, 1.5, 1H, ArH), 7.12 (dd, J = 7.5, 1.5, 1H, ArH), 4.62-4.31 (m, 1H, H5), 3.39-2.96 (m, br, 1H, H7a), 2.78 (dd, J = 15.8, 9.6, 1H, H7b), 2.58 (s, 3H, H3), 2.27-2.17 (m, br, 1H, H6a), 2.03-1.93 (m, br, 1H, H6b), 1.86 (s, 3H, H1), 1.70 (d, J = 7.3, 3H, H15). **^{13}C NMR** (125 MHz, $CDCl_3$): δ_C 173.4 (C14), 162.0 (C4), 140.7 (Ar), 138.6 (Ar), 135.4 (ArH), 129.2 (ArH), 126.8 (ArH), 126.7 (ArH), 65.8 (C2), 53.6 (C5), 32.7 (C6), 32.5 (C7), 25.8 (C3), 21.1 (C1), 20.5 (C15). **HRMS m/z** (ESI^+): calcd. For $C_{15}H_{20}N_3O$ [M+H] Calculated. 258.1601. Found. 258.1603. **IR ν_{max}** (ATR)/cm^{-1}: 3241, 2923, 2856, 1720, 1658.

Minor diastereoisomer (2g'): (11 mg, 0.0427 mmol, 21%) as a white solid. **R*f*** = 0.43 (3:1 EtOAc:PE). **^{1}H NMR** (400 MHz, $CDCl_3$): δ_H 7.40-7.38 (m, 1H, ArH), 7.25-7.22 (m, 2H, ArH), 7.12-7.10 (m, 1H, ArH), 4.33-4.23 (m, 1H, H5), 2.84-2.77 (m, 1H, H7a), 2.66 (s, 3H, H3), 2.51 (dd, J = 16.2, 9.2, 1H, H7b), 2.19-2.11 (m, br, 1H, H6a), 2.04 (ddd, J = 14.7, 9.9, 4.6, 1H, H6b), 1.85 (s, 3H, H1), 1.57 (d, J = 7.0, 3H, H15). **^{13}C NMR** (101 MHz, $CDCl_3$): δ_C 173.1 (C14), 162.0 (C4),

140.7 (Ar), 138.6 (Ar), 135.4 (ArH), 129.2 (ArH), 126.8 (ArH), 126.7 (ArH), 65.8 (C2), 53.6 (C5), 32.7 (C6), 32.5 (C7), 25.8 (C3), 21.1 (C1), 20.5 (C15). **HRMS m/z** (ESI$^+$): calcd. For $C_{15}H_{20}N_3O$ [M+H] Calculated. 258.1601. Found. 258.1600. **IR ν$_{max}$** (ATR)/cm^{-1}: 3244, 2926, 2853, 1732, 1653.

15-Imino-2,3,7,8,9,10-hexahydro-1*H*,5*H*-6,14b-methanobenzo[*e*]pyrrolo[1,2-*c*]diazecine-5-one (11a):

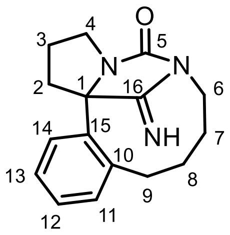

According to GP3, 1-(2,3,4,5-tetrahydro-1*H*-benzo[*b*]azepine-1-carbonyl)pyrrolidine-2-carbonitrile (68 mg, 0.253 mmol) and KHMDS (1M in THF, 0.51 mL, 0.506 mmol) in anhydrous THF stirred at 0 °C for 1 h. Purification via flash column chromatography eluting with MeOH:DCM (0:1 – 1:9) affording the title compound (39 mg, 0.145 mmol, 57%) as a white solid. **R$_f$** = 0.33 (25:75 MeOH:DCM). **^{1}H NMR** (400 MHz, $CDCl_3$): δ_H 7.41-7.35 (m, 1H, ArH), 7.26-7.18 (m, 2H, ArH), 7.15-7.09 (m, 1H, ArH), 3.93 (ddd, J = 13.9, 11.6, 5.4, 1H, CH_2), 3.79 (ddd, J = 13.9, 5.9, 1.9, 1H, CH_2), 3.70 (dt, J = 11.1, 7.1, 1H, CH_2), 3.19 (ddd, J = 11.1, 6.9, 5.7, 1H, CH_2), 3.04 (ddd, J = 15.3, 6.4, 2.7, 1H, CH_2), 2.94-2.76 (m, 2H, 2xCH_2), 2.15-1.83 (m, 5H, 4xCH_2), 1.82-1.72 (m, 1H, CH_2), 1.51 (ddddd, J = 15.1, 10.2, 3.7, 2.7, 1.2, 1H, CH_2). **^{13}C NMR** (101 MHz, $CDCl_3$): δ_C 172.7 (C5), 162.0 (C16), 143.4 (Ar), 137.0 (Ar), 134.2 (ArH), 129.0 (ArH), 126.4 (ArH), 125.6 (ArH), 74.5 (C1), 45.1 (CH_2), 40.7 (CH_2), 33.8 (CH_2), 32.2 (CH_2), 28.8 (CH_2), 26.8 (CH_2), 24.5 (CH_2). **HRMS m/z** (ESI$^+$): calcd. For $C_{16}H_{20}N_3O$ [M+H] Calculated. 270.1601. Found. 270.1609. **IR ν$_{max}$** (ATR)/cm^{-1}: 3270, 2953, 2869, 1723, 1645. **m.p.** 173-175 °C.

16-Imino-2,3,8,9,10,11-hexahydro-1*H*,5*H*,7*H*-6,15b-methanobenzo[*e*]pyrrolo[1,2-*c*]diazacycloundecin-5-one (11b):

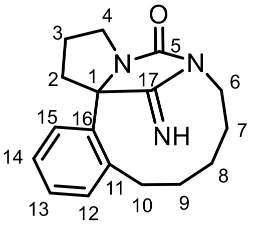

According to GP3, 1-(1,2,3,4,5,6-hexahydrobenzo[*b*]azocine-1-carbonyl)pyrrolidine-2-carbonitrile (45 mg, 0.159 mmol) and KHMDS (0.32 mL, 0.318 mmol) in anhydrous THF stirred at 0 °C for 1 h. Purification via flash column chromatography eluting with MeOH:DCM (0:1 – 1:9) afforded the title compound (33 mg, 0.117 mmol, 73%) as a yellow gum. $\mathbf{R_f}$ = 0.44 (25:75 MeOH:DCM). **^{1}H NMR** (400 MHz, $CDCl_3$): δ_H 7.39 (dd, J = 7.8, 1.5, 1H,ArH), 7.29-7.24 (m, 1H, ArH), 7.22 (dd, J = 7.5, 1.9, 1H, ArH), 7.20-7.15 (m, 1H, ArH), 3.84-8.62 (m, 3H, $2xCH_2$), 3.16 (ddd, J = 11.1, 8.6, 3.5, 1H, CH_2), 3.04-2.86 (m, 2H, $2xCH_2$), 2.46-2.32 (m, 1H, CH_2), 2.25-2.05 (m, 3H, $2xCH_2$), 1.88-1.77 (m, 2H, $2xCH_2$), 1.73-1.55 (m, 3H, $2xCH_2$), 1.50 (ddt, J = 11.5, 9.7, 5.0, 1H, CH_2). **^{13}C NMR** (101 MHz, $CDCl_3$): δ_C 170.0 (C5), 160.9 (C17), 144.6 (Ar), 134.9 (Ar), 133.5 (ArH), 129.0 (ArH), 126.3 (ArH), 126.0 (ArH), 73.4 (C1), 44.3 (CH_2), 39.8 (CH_2), 33.1 (CH_2), 30.7 (CH_2), 28.3 (CH_2), 26.5 (CH_2), 25.5 (CH_2), 23.5 (CH_2). **HRMS m/z** (ESI^+): calcd. For $C_{17}H_{22}N_3O$ Calculated. 284.1757. Found. 284.1764. **IR ν_{max}** (ATR)/cm^{-1}: 3247, 2923, 1722, 1654.

15-Imino-1,2,3,4,9,10-hexanhydro-6*H*,8*H*-7,14b-methanobenzo[*e*]pyrido[1,2-*c*]diazonin-6-one (11c):

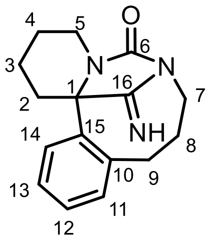

According to GP3, 1-(1,2,3,4-tetrahydroquinoline-1-carbonyl)piperidine-2-carbonitrile (50 mg, 0.186 mol) and KHMDS (1M in THF, 0.37 mL, 0.372 mmol) in anhydrous THF stirred at 0 °C for 1 h. Purification via flash column chromatography eluting with EtOAc:PE (3:7 – 1:0)

afforded the title compound (34 mg, 0.126 mmol, 68%) as a yellow oil. **R*f*** = 0.23 (1:0 EtOAc:PE). **^{1}H NMR** (400 MHz, $CDCl_3$): δ_H 7.46 (d, J = 7.6, 1H, ArH), 7.28-7.20 (m, 2H, ArH), 7.12 (d, J = 7.4, 1H, ArH), 4.04 (dd, J = 13.5, 4.7, 1H, H2a), 3.85 (td, J = 13.2, 5.9, 1H, H5a), 3.62-3.53 (m, br, 1H, H5b), 2.93 (d, br, J = 13.4, 1H, CH_2), 2.79-2.64 (m, 2H, CH_2), 2.55 (td, J = 13.2, 3.4, 1H, H2b), 2.22-2.10 (m, br, 1H, CH_2), 2.04-1.95 (m, 2H, CH_2), 1.91-1.76 (m, 2H, CH_2), 1.72 (d, br, J = 13.2, 1H, CH_2), 1.56-1.44 (m, 1H, CH_2). **^{13}C NMR** (101 MHz, $CDCl_3$): δ_C 171.2 (C6), 159.6 (C16), 141.5 (Ar), 137.5 (Ar), 135.7 (ArH), 128.9 (ArH), 127.5 (ArH), 126.7 (ArH), 63.7 (C1), 41.9 (C5), 37.5 (C2), 31.4 (CH_2), 30.6 (CH_2), 25.7 (CH_2), 21.2 (CH_2). **HRMS m/z** (ESI^+): calcd. For $C_{16}H_{20}N_3O$ [M+H] Calculated. 270.3480. Found. 270.1601. **IR ν_{max}** (ATR)/cm^{-1}: 3244, 2950, 2851, 1728, 1666.

***tert*-Butyl 15-imino-6-oxo-1,2,3,4,8,9-hexahydro-6*H*,10*H*-7,14b-methanobenzo[*g*]pyrido[2,1-*i*]triazonine-10-carboxylate (11d):**

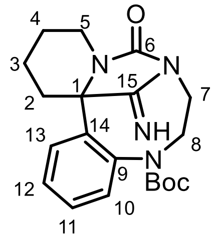

According to GP3, *tert*-butyl 4-(2-cyanopiperidine-1-carbonyl)-3,4-dihydroquinoxaline-1(2*H*)-carboxylate (50 mg, 0.135 mmol) and KHMDS (1M in THF, 0.27 mL, 0.270 mmol) in anhydrous THF stirred at 0 °C for 1.5 h. Purification via flash column chromatography with MeOH:DCM (1:99 – 1:9) afforded the title compound (14 mg, 0.0378 mmol, 28%) as a brown oil. **R*f*** = 0.28 (5:95 MeOH:DCM). **^{1}H NMR** (400 MHz, $CDCl_3$) (mixture of rotamers A:B in a 0.67:0.33 ratio): δ_H 7.48 (dt, J = 7.1, 1.7, 1H, ArH, rot. A+B), 7.38-7.30 (m, 2H, ArH, rot. A+B), 7.16-7.12 (m, 0.33H, ArH, rot. B), 7.05-7.01 (m, 0.67H, ArH, rot. A), 4.31 (ddd, J = 13.7, 11.9, 5.2, 0.67H, CH_2, rot. A), 4.26-4.23 (m, 0.33H, CH_2, rot. B), 4.11 (dd, J = 14.0, 5.2, 067H, CH_2, rot. A), 3.98 (ddt, J

= 13.3, 5.0, 1.6, 1H, CH_2, rot. A+B), 3.94-3.90 (m, 0.66H, 2xCH_2, rot. B), 3.59-3.53 (m, 1H, CH_2, rot. A+B), 3.27 (ddd, J = 14.4, 12.0, 5.4, 0.33H, CH_2, rot. B), 3.17 (ddd, J = 14.0, 11.9, 5.4, 0.67H, CH_2, rot. A), 2.95-2.89 (m, 1H, CH_2, rot. A+B), 2.62 (td, J = 13.4, 3.3, 0.33H, CH_2, rot. B), 2.52 (td, J = 13.3, 3.3, 0.67H, CH_2, rot. A), 2.04-1.95 (m, 1H, CH_2, rot. A+B), 1.90-1.78 (m, 2, CH_2, rot. A+B), 1.67-1.64 (m, 1H, CH_2, rot. A+B), 1.50-1.44 (m, 1H, CH_2, rot. A+B), 1.53 (s, 3H, Boc-CH_3, rot. B), 1.33 (s, 6H, Boc-CH_3, ro.t A). **^{13}C NMR** (101 MHz, $CDCl_3$): δ_C 173.1 (C6, rot. B), 172.6 (C6, rot. A), 158.1 (C15, rot. A), 158.0 (C15, rot. B), 154.8 (Boc-CO, rot. B), 154.5 (Boc-CO, rot. A), 143.3 (Ar, rot. B), 143.1 (Ar, rot. A), 135.5 (Ar, rot. B), 134.7 (Ar, rot. A), 133.5 (ArH, rot. B), 132.9 (ArH, rot. A), 130.1 (ArH, rot. B), 129.6 (ArH, rot. A), 127.9 (ArH, rot. A+B), 127.6 (ArH, rot. A+B), 81.6 (Boc-C(Me)$_3$, rot. A), 80.7 (Boc-C(Me)$_3$, rot. B), 63.1 (C1, rot. B), 62.9 (C1, rot. A), 47.3 (CH_2, rot. B), 46.6 (CH_2, rot. A), 43.6 (CH_2, rot. B), 42.7 (CH_2, rot. A), 37.6 (CH_2, rot. A), 37.6 (CH_2, rot. A), 37.4 (CH_2, rot. B), 29.6 (CH_2, rot. A+B), 28.4 (Boc-CH_3, rot. A), 28.1 (Boc-CH_3, rot. B), 25.0 (CH_2, rot. A+B), 24.8 (CH_2, rot. A+B), 21.0 (CH_2, rot. A+B). **HRMS m/z** (ESI$^+$): calcd. For $C_{20}H_{27}N_4O_3$ [M+H] Calculated. 371.2078. Found. 271.2074. **IR ν_{max}** (ATR)/cm^{-1}: 3265, 2979, 2947, 2870, 1737, 1697, 1663.

15-Imino-1,2,3,4,8,9-hexahydro-6*H*,7,14b-methanobenzo[*h*]pyrido[1,2-*f*][1,4,6]thiadiazonin-6-one (11e):

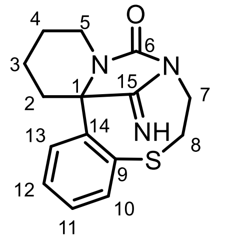

According to GP3, 1-(3,4-dihydro-2*H*-benzo[*b*][1,4]thiazine-4-carboyl)piperidine-2-carbonitrile (100 mg, 0.348 mmol) and KHMDS (1M in THF, 0.70 mL, 0.696 mmol) in anhydrous THF stirred at 0 °C for 1.5 h. Purification via flash column chromatography eluting with MeOH:DCM (0:1 – 1:9) afforded the title compound (36 mg, 0.125 mmol, 36%) as an orange

oil. **R$_f$** = 0.57 (5:95 MeOH:DCM). **^{1}H NMR** (500 MHz, $CDCl_3$): δ_H 7.69 (dd, J = 7.7, 1.6, 1H, ArH), 7.54 (dd, J = 7.9, 1.5, 1H, ArH), 7.40 (td, J = 7.9, 1.6, 1H, ArH), 7.24 (td, J = 7.7, 1.5, 1H, ArH), 4.04-4.00 (m, 1H, CH_2), 3.92 (td, J = 13.5, 5.0, 1H, CH_2), 3.77 (dd, J = 13.5, 5.5, 1H, CH_2), 3.35 (ddd, J = 14.9, 12.5, 5.5, 1H, CH_2), 3.05 (dd, J = 14.9, 5.0, 1H, CH_2), 2.90 (dd, br, J = 11.2, 3.9, 1H, CH_2), 2.46 (td, J = 1.5, 3.4, 1H, CH_2), 2.05-1.95 (m, br, 1H, CH_2), 1.87-1.77 (m, 2H, CH_2), 1.69 (d, br, J = 13.3, 1H, CH_2), 1.56-1.47 (m, 1H, CH_2). **^{13}C NMR** (125 MHz, $CDCl_3$): δ_C 172.9 (C6), 158.8 (C15), 142.3 (ArH), 141.5 (Ar), 133.9 (Ar), 129.5 (ArH), 129.1 (ArH), 128.4 (ArH), 63.7 (C1), 42.3 (CH_2), 37.6 (CH_2), 35.4 (CH_2), 30.9 (CH_2), 25.2 (CH_2), 21.1 (CH_2). **HRMS m/z** (ESI$^+$): calcd. For $C_{15}H_{18}N_3OS$ [M+H] Calculated. 288.1171. Found. 288.1181. **IR ν$_{max}$** (ATR)/cm^{-1}: 3239, 2923, 2845, 1732, 1660.

NB: Oxidation of starting urea **10e** during the reaction resulted in isolation of 1-(3,4-dihydro-2*H*-benzo[*b*][1,4]thiazine-4-carbonyl)piperidine-2-carbonitrile (26%).

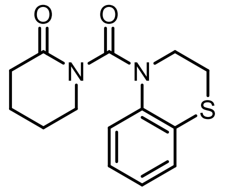

16-Imino-1,2,3,4,8,9,10,11-octahydro-6*H*-7,15b-methanobenzo[*e*]pyrido[1,2-*c*][1,3]diazecine-6-one (11f):

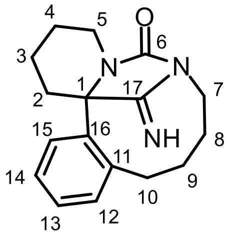

According to GP3, 1-(2,3,4,5,-tetrahydro-1*H*-benzo[*b*]azepine-1-carbonyl)piperidine-2-carbonitrile (70 mg, 0.247 mmol) and KHMDS (1M in THF, 0.49 mL, 0.494 mmol) in anhydrous THF stirred at 0 °c for 1.5 h. Purification via flash column chromatography eluting with MeOH:DCM (1:99 – 1:9) afforded the title compound (43 mg, 0.152 mmol, 61%) as a yellow foam. **R$_f$** = 0.59 (5:95 MeOH:DCM). **^{1}H NMR** (400 MHz, $CDCl_3$): δ_H 7.7-7.43 (m, 1H, ArH), 7.27-7.20 (m, 2H, ArH), 7.12-7.08 (m, 1H, ArH), 6.84 (s, br, 1H, NH), 4.10 (ddt, J = 13.6, 5.1, 1.6, 1H,

CH_2), 4.05-3.93 (m, 1H, CH_2), 3.80 (ddd, J = 1.8, 6.0, 1.4, 1H, CH_2), 2.89 (d, br, J = 14.1, 1H, CH_2), 2.77 (ddd, J = 15.4, 12.4, 6.5, 1H, CH_2), 2.70-2.57 (m, 2H, CH_2), 2.06-1.91 (m, 2H, CH_2), 1.89-1.73 (m, 3H, CH_2), 1.70-1.62 (m, 2H, CH_2), 1.57-1.43 (m, 2H, CH_2). **^{13}C NMR** (101 MHz, $CDCl_3$): δ_C 172.2 (C6), 156.9 (C17), 143.5 (Ar), 134.9 (Ar), 134.4 l(ArH), 129.0 (ArH), 128.2 (ArH), 126.4 (ArH), 64.8 (C1), 40.2 (CH_2), 37.5 (CH_2), 33.7 (CH_2), 32.5 (CH_2), 29.3 (CH_2), 25.8 (CH_2), 24.4 (CH_2), 21.1 (CH_2). **HRMS m/z** (ESI^+): calcd. For $C_{17}H_{22}N_3O$ [M+H] Calculated. 284.1685. Found. 284.1756. **IR ν_{max}** (ATR)/cm^{-1}: 3272, 2937, 2891, 2856, 1722, 1735, 1651.

17-Imino-1,2,3,4,9,10,11,12-octahydro-6*H*,8*H*-7,16b-methanobenzo[*e*]pyrido[1,2-*c*][1,3]diazacycloundecin-6-one (11g):

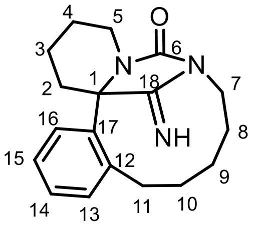

According to GP3, 1-(1,2,3,4,5,6-hexahydrobenzo[*b*]azocine-1-carbonyl)piperidine-2-carbonitrile (185 mg, 0.622 mmol) and KHMDS (1M in THF, 1.24 mL, 1.24 mmol) in anhydrous THF stirred at 0 °C for 1.5 h. The title compound (163 mg, 0.548 mmol, 88%) was obtained without any further purification as a yellow oil. **R_f** = 0.24 (1:0 EtOAc:PE). **^{1}H NMR** (400 MHz, $CDCl_3$): δ_H 7.51-7.46 (m, 1H, ArH), 7.28-7.22 (m, 2H, ArH), 7.18-7.13 (m, 1H, ArH), 6.74 (s, br, 1H, NH), 4.07 (ddt, br, J = 13.5, 4.9, 1.6, 1H, H2a), 3.81-3.71 (m, br, 2H, H7), 2.87 (d, br, J = 14.1, 1H, CH_2), 2.65-2.58 (m, 1H, CH_2), 2.53 (m, 1H, H2b), 2.36 (dt, J = 14.1, 9.6, 1H, CH_2), 2.23-2.12 (m, 1H, CH_2), 1.94-1.78 (m, 3H, 2 x CH_2), 1.67-1.58 (m, 5H, 3 x CH_2), 1.53-1.40 (m, 2H, CH_2). **^{13}C NMR** (101 MHz, $CDCl_3$): δ_C 170.7 (C6), 156.2 (C18), 144.8 (Ar), 133.9 (ArH), 133.8 (Ar), 129.3 (ArH), 129.2 (ArH), 126.3 (ArH), 64.3 (C1), 40.0 (C7), 37.9 (C2), 34.2 (CH_2), 31.2 (CH_2), 29.4 (CH_2), 26.1 (CH_2), 25.8 (CH_2), 24.1 (CH_2), 21.5 (CH_2). **HRMS m/z** (ESI^+): calcd. For $C_{18}H_{24}N_3O$

[M+H] Calculated. 298.1841. Found. 298.1913. **IR ν_{max}** (ATR)/cm^{-1}: 3551, 3493, 3283, 3252, 3824, 2835, 1717, 1648.

3^1-Amino-1,10b-dimethyl-1,3^1,4,5,5a,10b-hexahydrobenzo[*f*]imidazo[4,5,1-*hi*]indole-2,6-dione (13):

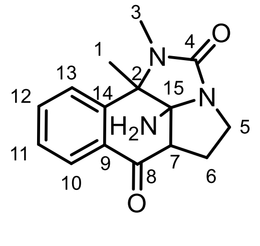

Accoridng to GP3, *N*-(1-cyanoethyl)-*N*-methyl-5-oxo-2,3,4,5-tetrahydro-1*H*-benzo[*b*]azepine-1-carboxamide (29 mg, 0.107 mmol) and KHMDS (1M in THF, 0.22 mL, 0.222 mmol) in anhydrous THF stirred at 0 °C for 1.5 h. Purification via flash column chromatography eluting with MeOH:DCM (2:98 – 1:9) afforded the title compound (16 mg, 0.0590 mmol, 55%) as a colourless solid. **R_f** = 0.54 (1:9 MeOH:DCM). **^{1}H NMR** (400 MHz, $CDCl_3$): δ_H 7.73 (ddd, J = 7.5, 1.5, 0.6, 1H, ArH), 7.63-7.54 (m, 2H, ArH), 7.49 (td, J = 7.3, 1.4, 1H, ArH), 4.07 (dddd, J = 12.2, 9.2, 2.2, 0.7, 1H, H5a), 3.21 (ddd, J = 12.2, 9.9, 8.0, 1H, H5b), 2.73-2.68 (m, 1H, H7), 2.57-2.47 (m, 1H, H6a), 2.25 (s, 3H, H3), 2.13 (dddd, J = 13.6, 9.9, 8.0, 2.2, 1H, H6b), 1.79 (s, 3H, H1). **^{13}C NMR** (101 MHz, $CDCl_3$): δ_C 197.6 (C8), 160.4 (C4), 139.7 (Ar), 134.2 (Ar), 132.6 (ArH), 129.2 (ArH), 127.1 (ArH), 126.4 (ArH), 83.7 (C15), 61.4 (C2), 56.0 (C7), 44.7 (C5), 25.1 (C3), 23.8 (C6), 15.0 (C1). **HRMS m/z** (ESI$^+$): calcd. For $C_{15}H_{17}N_3O_2Na$ [M+Na] Calculated. 294.1213. Found. 294.1204. **IR ν_{max}** (ATR)/cm^{-1}: 3391, 3316, 2973, 2923, 2850, 1682. **m.p.** 157-160 °C.

tert-Butyl 12-imino-6,7-dimethyl-5-oxo-2,3,6,7-tetrahydro-4,7-methanobenzo[_gi_][1,3,6]triazonine-1(5_H_)-carboxylate (15):

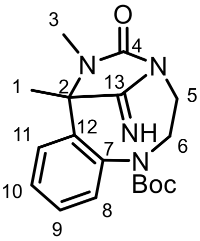

According to GP3, _tert_-butyl 4-((1-cyanoethyl)(methyl)carbamoyl)-3,4-dihydroquinoxaline-1(2_H_)-carboxylate (49 mg, 0.145 mmol) and KHMDS (1M in THF, 0.29 mL, 0.290 mmol) in anhydrous THF stirred at 0 °C for 1 h. Purification via flash column chromatography eluting with MeOH:DCM (0:1 – 1:9) afforded the title compound (32 mg, 0.0929 mmol, 64%) as an orange oil. **R_f** = 0.65 (1:9 MeOH:DCM). **^{1}H NMR** (400 MHz, $CDCl_3$) (mixture of rotamers A:B in a 0.67:0.33 ratio): δ_H 7.43-7.40 (m, 1H, ArH, rot. A+B), 7.37-7.28 (m, 2H, ArH, rot. A+B), 7.12 (dd, J = 7.4, 1.8, 0.33H, ArH, rot. B), 7.02-6.98 (m, 0.67H, ArH, rot. A), 4.34-4.23 (m, 1H, CH_2, rot A+B), 4.13-4.06 (m, 0.67H, CH_2, rot. A), 3.89 (dd, J = 14.3, 4.9, 0.33H, CH_2, rot. B), 3.60-3.48 (m, 1H, CH_2, rot. A+B), 3.35-3.30 (m, 0.33H, CH_2, rot. B), 3.28-3.17 (m, 0.67H, CH_2, rot. A), 2.64 (s, 2H, H3, rot. A), 2.62 (s, 1H, H3, rot. B), 1.86 (s, 3H, H3, rot. A+B), 1.53 (s, 3H, Boc-CH_3, rot. B), 1.30 (s, 6H, Boc-CH_3, rot. A). **^{13}C NMR** (125 MHz, $CDCl_3$): δ_C 172.2 (C4, rot. B), 171.2 (C4, rot. A), 160.6 (C13, rot. B), 160.4 (C13, rot. A), 155.0 (Boc-CO, rot. B), 154.6 (Boc-CO, rot. A), 142.3 (Ar, rot. B), 142.2 (Ar, rot. A), 136.8 (Ar, rot. B), 136.1 (Ar, rot. A), 133.3 (ArH, rot B), 132.6 (ArH, rot. A), 130.3 (ArH, rot. B), 129.7 (ArH, rot. A), 128.0 (ArH, rot. B), 127.7 (ArH, rot. A), 127.0 (ArH, rot. A+B), 81.7 (Boc-C(Me)$_3$, rot. B), 80.9 (Boc-C(Me)$_3$, rot. A), 65.2 (C2, rot. B), 65.1 (C2, rot. A), 46.8 (CH_2, rot. B), 46.1 (CH_2, rot. A), 43.9 (CH_2, rot. B), 43.0 (CH_2, rot. A), 28.3 (Boc-CH_3, rot. A), 28.2 (Boc-CH_3, rot. B), 25.6 (C3, rot. A+B), 20.0 (C1, rot. B), 19.9 (C1, rot. A). **HRMS m/z** (ESI$^+$): calcd. For $C_{18}H_{24}N_4O_3$ [M+H] Calculated. 345.1921. Found. 345.1919. **IR ν_{max}** (ATR)/cm^{-1}: 3191, 2978, 2932, 1741, 1707, 1665.

15,16-Diimino-1,2,9,10-tetramethyl-1,2,5,6,9,10-hexahydro-1,4:7,10-dimethanobenzo[*j*][1,3,6,8]tetraazacyclododecine-3,8-dione (18a & 18b):

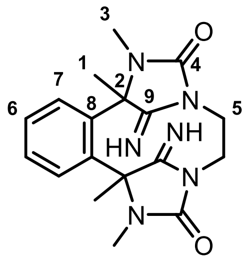

According to a modified GP3, N^1,N^4-bis(1-cyanoethyl)-N^1,N^4-dimethyl2,3-dihydroquinoxaline-1,4-dicarboxamide (306 mg, 0.863 mmol) and KHMDS (1M in THF, 3.5 mL, 3.45 mmol) in anhydrous THF stirred at 0 °C for 5 h. The reaction mixture was quenched with MeOH (2 mL) and the volatiles removed under reduced pressure. Purification via column chromatography eluting with MeOH:DCM (5:95 – 2:8) afforded the title compound as a mixture of diastereoisomers:

***Meso*-(major)-diastereoisomer (18a):**

(125 mg, 0.353 mmol, 41%) as a yellow solid. **R_f** = 0.32 (1:9 MeOH:DCM). **^{1}H NMR** (500 MHz, MeOD-d_4): δ_H 7.93 (dd, J = 6.0. 3.6, 2H, ArH), 7.61 (dd, J = 6.0, 3.5, 2H, ArH), 4.22-4.14 (m, 2H, H5a), 4.10-4.02 (m, 2H, H5b), 2.60 (s, 6H, H3), 1.91 (s, 6H, H1). **^{13}C NMR** (125 MHz, MeOD-d_4): δ_C 167.4 (C4), 156.0 (C9), 135.6 (Ar), 132.5 (ArH), 129.5 (ArH), 65.7 (C2), 35.8 (C5), 27.6 (C1), 24.7 (C3). **HRMS m/z** (ESI^+): calcd. For $C_{18}H_{23}N_6O_2$ [M+H] Calculated. 255.1877. Found. 355.1869. **IR ν_{max}** (ATR)/cm^{-1}: 3262, 2946, 1745, 1668. **m.p.** 288-291 °C.

***C_2 symmetric*-(minor)-diastereoisomer (18b):**

(44 mg, 0.124 mmol, 14%) as a yellow solid. **R_f** = 0.44 (1:9 MeOH:DCM). **^{1}H NMR** (500 MHz, MeOD-d_4): δ_H 7.96-7.93 (m, 2H, ArH), 7.58-7.55 (m, 2H, ArH), 4.18-4.05 (m, br, 2H, H5a), 3.81-3.79 (m, br, 2H, H5b), 2.63 (s, 6H, H3), 1.87 (s, 6H, H1). **^{13}C NMR** (125 MHz, MeOD-d_4): δ_C 176.9 (C4), 157.5 (C9), 137.2 (Ar), 133.8 (ArH), 130.6 (ArH), 66.7 (C2), 38.4 (C5), 29.5 (C1), 25.2 (C3).

HRMS m/z (ESI+): calcd. For $C_{18}H_{23}N_6O_2$ [M+H] Calculated. 355.1877. Found. 355.1892. **IR ν_{max}** (ATR)/cm^{-1}: 3271, 3222, 2976, 2960, 1729, 1659. **m.p.** 234-236 °C.

HPLC: Chiral OJ-H column, MeOH:EtOH = 5:95, flow = 0.3 mL/min, 230 nm.

***Meso*-(major)-diastereoisomer (18a):** t_R = 14.6 min.

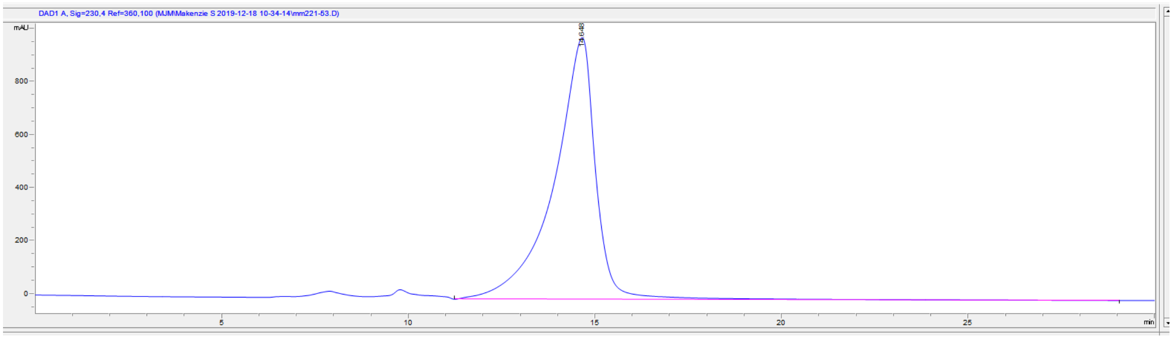

Peak #	Time / min	Area	Height	Width	Area / %	Symmetry
1	14.648	77686.2	990	1.0895	100.000	1.89

***Racemic*-(minor)-diastereoisomer (18b):** t_R = 14.9 min, 17.4 min, *er* = 49:51.

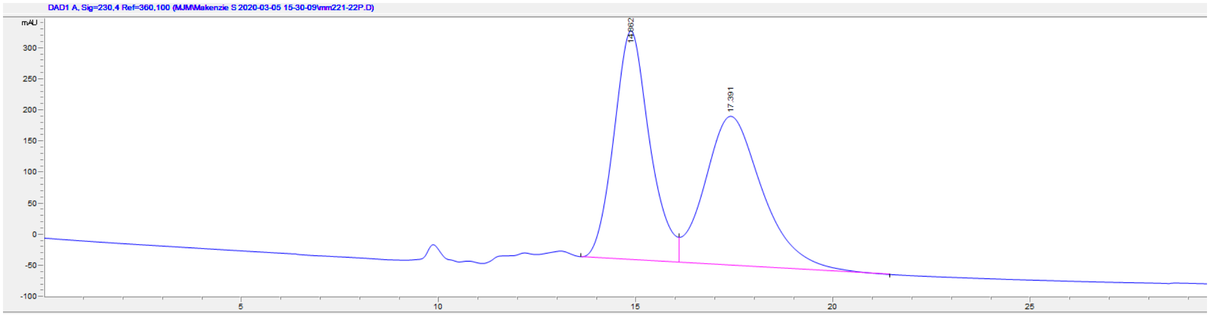

Peak #	Time / min	Area	Height	Width	Area / %	Symmetry
1	14.862	23223.8	367.4	0.9434	48.798	0.85
2	17.391	24367.9	239.6	1.5146	51.202	0.756

2.4. Hydrolysis Products

2a[1]-Hydroxy-1,8b-dimethyl-2a[1],3,4,8b-tetrahydro-1,2a,4a-triazapentaleno[1,6-*b*]inden-2(1*H*)-one (16):

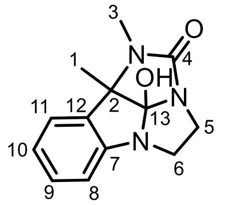

tert-Butyl 6,7-dimethyl-5,12-dioxo-2,3,6,7-tetrahydro-4,7-methanobenzo[*g*][1,3,6]triazonine-1(5*H*)-carboxylate (49 mg, 0.142 mmol) was dissolved in 2M HCl:MeOH (1:1 ratio, 0.05 M). The solution was refluxed for 44 h. The reaction mixture was cooled to room temperature and extracted into DCM (2 x 20 mL). The combined organic extracts were dried over Na_2SO_4, filtered and the solvent removed under reduced pressure. Purification via flash column chromatography eluting with MeOH:DCM (1:99 – 1:9) afforded the title compound (18 mg, 0.0734 mmol, 52%) as a yellow solid. $\mathbf{R_f}$ = 0.17 (1:20 MeOH:DCM). **^{1}H NMR** (400 MHz, $CDCl_3$) (mixture of rotamers A:B in a 0.78:0.22 ratio): δ_H 7.23-7.18 (m, 1H, ArH, rot. A+B), 7.11-7.08 (m, 1H, ArH, rot. A+B), 6.94-6.87 (m, 1H, ArH, rot. A+B), 6.74 (d, J = 7.7, 1H, ArH, rot. A+B), 4.07 (ddd, J = 11.8, 8.0, 3.7, 1H, H5a, rot. A+B), 3.96 (s, br, 1H, OH, rot. A+B), 3.80-3.70 (m, 1H, H6a, rot. A+B), 3.61-3.54 (m, 1H, H5b, rot. A+B), 3.22 (dt, J = 9.7, 8.0, 1H, H6b, rot. A+B), 2.74 (s, 2.34H, H3, rot. A), 2.72 (s, 0.66H, H3, rot. B), 1.67 (s, 2.34H, H1, rot. A), 1.66 (s, 0.66H, H1, rot. B). **^{13}C NMR** (101 MHz, $CDCl_3$): δ_C 161.0 (C4, rot. B), 160.3 (C4, rot. A), 150.8 (Ar, rot. B), 150.4 (Ar, rot. A), 132.7 (Ar, rot. A), 132.5 (Ar, rot. B), 130.2 (ArH, rot. A), 130.2 (ArH, rot. B), 123.5 (ArH, rot. B), 123.4 (ArH, rot. A), 121.9 (ArH, rot. A), 121.5 (ArH, rot. B), 113.9 (ArH, rot. A), 113.9 (ArH, rot. B), 102.7 (C13, rot. B), 102.6 (C13, rot. A), 68.7 (C2, rot. B), 68.3 (C2, rot A), 53.0 (C6, rot. A), 52.6 (C6, rot. B), 47.7 (C5, rot. B) 47.7 (C5, rot. A), 25.5 (C3, rot. B), 25.4 (C3, rot. A), 17.0 (C1, rot. B), 16.4 (C1, rot. A). **HRMS m/z** (ESI^+):

calcd. For $C_{13}H_{16}N_3O_2$ [M+H] Calculated. 246.1164. Found. 246.1257. **IR ν_{max}** (ATR)/cm^{-1}: 3292 (br), 2968, 2932, 2871, 1674, 1603. **m.p.** 186-188 °C.

1,2-Dimethyl-1,5,6,7-tetrahydro-1,4-methanobenzo[*e*][1,3]diazonine-3,12(2*H*)-dione (2a-*h*):

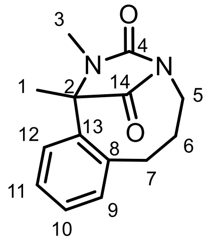

According to GP4, 12-imino-1,2-dimethyl-1,5,6,7-tetrahydro-1,4-methanobenzo[*e*][1,3]diazonin-3(2*H*)-one (37 mg, 0.152 mmol) dissolved in TFA:2M HCl (1:9 ratio, 3.04 mL, 0.05 M) in a microwave vial. The sealed vial was heated in the µW at 120 °C for 2 h. The title compound (28 mg, 0.115 mmol, 75%) was afforded without any further purification as a yellow oil. **R*f*** = 0.71 (1:1 EtOAc:PE). **^{1}H NMR** (400 MHz, $CDCl_3$): δ_H 7.40 (dd, J = 7.3, 1.7, 1H, ArH), 7.26 (quint. d, J = 7.3, 1.8, 2H, ArH), 7.12 (dd, J = 7.3, 1.8, 1H, ArH), 3.81-3.65 (m, br, 2H, H5), 2.96-2.78 (m, br, 1H, H7a), 2.73 (s, 3H, H3), 2.57 (dd, J = 16.1, 9.1, 1H, H7b), 2.20-2.10 (m, 1H, H6a), 2.07-1.99 (m, 1H, H6b), 1.87 (s, 3H, H1). **^{13}C NMR** (101 MHz, $CDCl_3$): δ_C 180.9 (C14), 172.9 (C4), 139.7 (Ar), 137.0 (Ar), 135.9 (ArH), 129.5 (ArH), 127.5 (ArH), 127.1 (ArH), 66.6 (C2), 40.7 (C5), 32.0 (C7), 29.8 (C6), 25.5 (C3), 18.0 (C1). **HRMS m/z** (ESI$^+$): calcd. For $C_{14}H_{16}N_2O_2Na$ [M+Na] Calculated. 267.1212. Found. 267.1103. **IR ν_{max}** (ATR)/cm^{-1}: 2923, 2859, 1771, 1778, 1707.

1,2,5-Trimethyl-1,5,6,7-tetrahydro-1,4-methanobenzo[*e*][1,3]diazonine-3,12(2*H*)-dione (2g-*h* & 2g'-*h*):

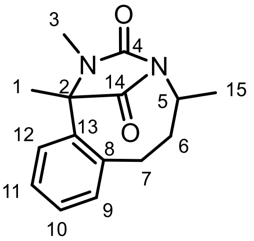

According to GP4, 12-imino-1,2,5-trimethyl-1,5,6,7-tetrahydro-1,4-methanobenzo[*e*][1,3]diazonin-3(2*H*)-one was dissolved in TFA:2M HCl (1:9 ratio, 0.05 M) in a microwave vial. The sealed vial was heated in the µW at 120 °C for 2 h.

Major diastereoisomer (2g-*h*):

12-imino-1,2,5-trimethyl-1,5,6,7-tetrahydro-1,4-methanobenzo[*e*][1,3]diazonin-3(2*H*)-one (15 mg, 0.0583 mmol) afforded the title compound (7 mg, 0.0271 mmol, 47%) as a yellow gum. **R*f*** = 0.80 (5:95 MeOH:DCM). **^{1}H NMR** (500 MHz, $CDCl_3$, 243 K/-30 °C) (mixture of rotamers A:B in a 0.70:0.30 ratio): δ_H 7.40 (d, J = 7.3, 0.3 H, ArH rot. B), 7.34-7.23 (m, 2H, ArH, rot. A+B), 7.23-7.18 (m, 0.7H, ArH, rot. A), 7.13 (d, J = 7.6, 1H, ArH), 4.59 (sextet, J = 7.4, 0.7H, H5, rot. A), 4.44 (quint., J = 7.7, 0.3H, rot. B), 3.26-3.14 (m, 0.7H, H7, rot. A), 2.82-2.73 (m, 1H, H7, rot. A+B), 2.76-2.73 (m, 0.3H, H7, rot. B), 2.72 (s, 1H, H3, rot. B), 2.58 (s, 2H, H3, rot. A), 2.38-2.24 (m, 0.3H, H6a, rot. B), 2.17-2.05 (m, 1.7H, H6, rot. A+B), 1.84 (s, 1H, H1, rot. B), 1.83 (s, 2H, H1, rot. A), 1.68 (d, J = 7.6, 1H, H15, rot. B), 1.62 (d, d = 7.2, 2H, H15, rot. A). **^{13}C NMR** (125 MHz, $CDCl_3$, 243 K/-30 °C): δ_C 182.7 (C14, rot. A), 181.7 (C14, rot. B), 159.3 (C4, rot. B), 158.4 (C4, rot. A), 137.5 (Ar,rot. A+B), 137.4 (Ar, rot. A+B), 136.7 (ArH, rot. A+B), 129.5 (ArH, rot. B), 128.8 (ArH, rot. A), 127.4 (ArH, rot. B), 127.1 (ArH, rot. B), 126.8 (ArH, rot. A), 126.8 (ArH, rot. A), 66.9 (C2, rot. A), 65.1 (C2, rot. B), 59.3 (C5, rot. B), 56.4 (C5, rot. A), 32.4 (C6 or 7, rot. A+B), 31.3 (C6 or 7, rot. A+B), 25.1 (C3, rot. B), 24.4 (C3, rot. A), 18.0 (C15 or 1, rot. B), 17.9 (C15 or 1, rot. B), 17.7 (C15, rot. A), 16.6 (C1, rot. A).

HRMS m/z (ESI$^+$): calcd. For $C_{15}H_{18}N_2NaO_2$ [M+Na] Calculated. 281.1260. Found. 281.1258. **IR ν_{max}** (ATR)/cm^{-1}: 2920, 2851, 1769, 1712.

Minor diastereoisomer (2g'-*h*):

12-imino-1,2,5-trimethyl-1,5,6,7-tetrahydro-1,4-methanobenzo[*e*][1,3]diazonin-3(2*H*)-one (23 mg, 0.0894 mmol) afforded the title compound (7 mg, 0.0271 mmol, 30%) as an orange oil. **R*f*** = 0.84 (5:95 MeOH:DCM). **^{1}H NMR** (400 MHz, $CDCl_3$): δ_H 7.33 (dd, J = 7.2, 2.0, 1H, ArH), 7.22-7.15 (m, 2H, ArH), 7.05 (dd, J = 7.0, 2.1, 1H, ArH), 4.14-4.04 (m, 1H, H5), 2.76 (ddd, J = 16.4, 9.2, 2.2, 1H, H7a), 2.65 (s, 3H, H3), 2.46 (ddd, J = 16.4, 8.0, 1.7, 1H, H7b), 2.08-1.94 (m, 2H, H6), 1.78 (s, 3H, H1), 1.49 (d, J = 6.9, 3H, H15). **^{13}C NMR** (101 MHz, $CDCl_3$): δ_C 181.3 (C14), 160.5 (C4), 140.0 (Ar), 137.0 (Ar), 135.7 (ArH), 129.5 (ArH), 127.6 (ArH), 127.1 (ArH), 66.3 (C2), 52.3 (C5), 33.4 (C6), 32.7 (C7), 25.6 (C3), 19.4 (C15), 18.4 (C1). **HRMS m/z** (ESI$^+$): calcd. For $C_{15}H_{18}N_2O_2Na$ [M+H] Calculated. 259.1441. Found. 259.1436. **IR ν_{max}** (ATR)/cm^{-1}: 2932, 2852, 1771, 1706.

2.5. *In situ* ReactIR Experimental Data

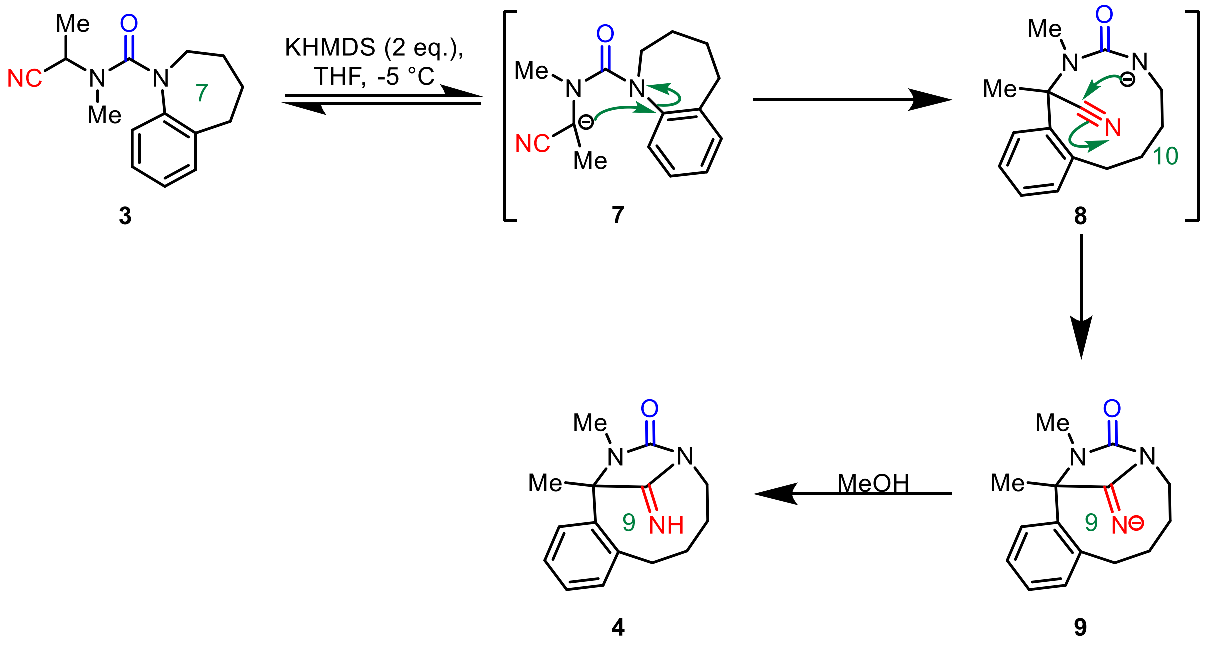

A react IR probe was fitted into a flame-dried three-necked round bottom flask, which was cooled to room temperature under vacuum. Under an atmosphere of N_2 anhydrous THF (2.6 mL) was added, the reaction flask was cooled to -5 °C for 30 min, then an IR solvent background spectrum was taken. The solvent was removed via syringe and the flask placed under vacuum to remove any residual solvent. In a separate flask *N*-(1-cyanoethyl)-*N*-methyl-2,3,4,5-tetrahydro-1*H*-benzo[*b*]azepine-1-carboxamide (100 mg, 0.389 mmol) was dissolved in anhydrous THF (2.6 mL, 0.3 M) and the solution was transferred to the reaction flask via syringe. The reaction flask was cooled to -5 °C for 30 min before the ReactIR was started and spectra were recorded every 30 seconds. After 5 min the scan rate was changed to record a spectra every 15 seconds and KHMDS (1M in THF, 0.79 mL, 0.778 mmol, 1M in THF) was added dropwise over 90 seconds. These is a shift in absorption of the urea carbonyl from 1654 cm^{-1} to 1663 cm^{-1} along with the appearance of an absorption at 1737 cm^{-1}. The absorption at 1663 cm^{-1} immediately (within 15 seconds) shifts to 1660 cm^{-1}. The absorptions at 1660 and 1737 cm^{-1} correspond to anion **9**, assigned based on IR spectrum obtained when **4** was treated with

KHMDS (see Figure S2). The reaction mixture was left to stir for a further 1.5 h (no further changes in the spectrum were recorded) at -5 °C before quenching with MeOH (1 mL). Spectra were recorded for a further 10 min. Upon quenching with MeOH three new absorptions at 1662, 1736 and 1743 cm^{-1} were detected corresponding to product **4**.

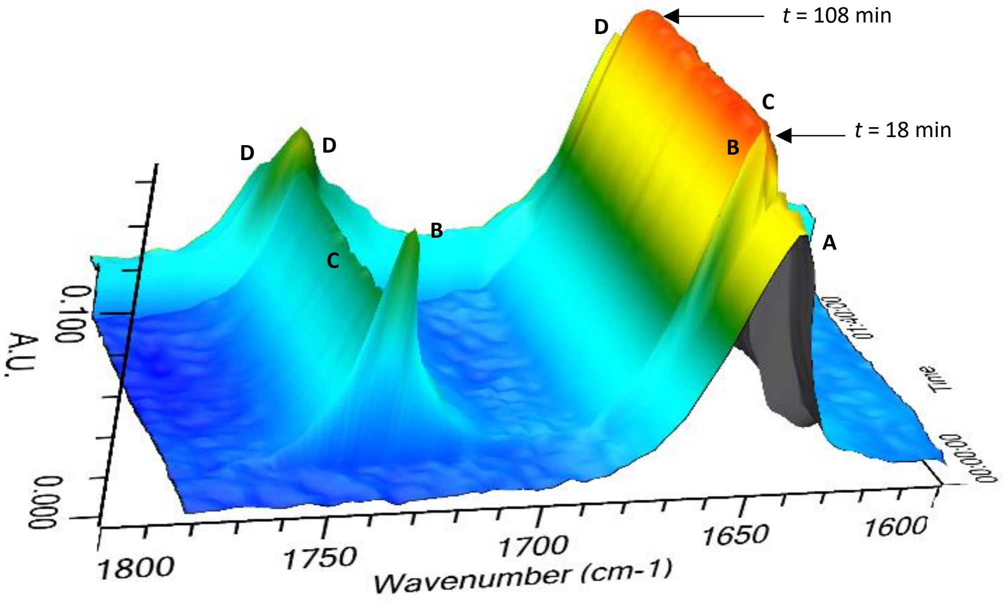

Figure S1: Time course following the reaction of 3 with KHMDS at -5 °C in THF by *in situ* ReactIR. **A: 3** in THF at -5 °C, ν = 1654 cm^{-1}; **B:** (*t* = 18 min) KHMDS (2 equiv.) added, ν = 1663 & 1737 cm^{-1}; **C:** Reaction mixture at -5 °C, ν 1660 & 1737 cm^{-1}; **D:** (*t* = 108 min) Reaction quenched by MeOH addition, ν = 1662, 1736 & 1743 cm^{-1}.

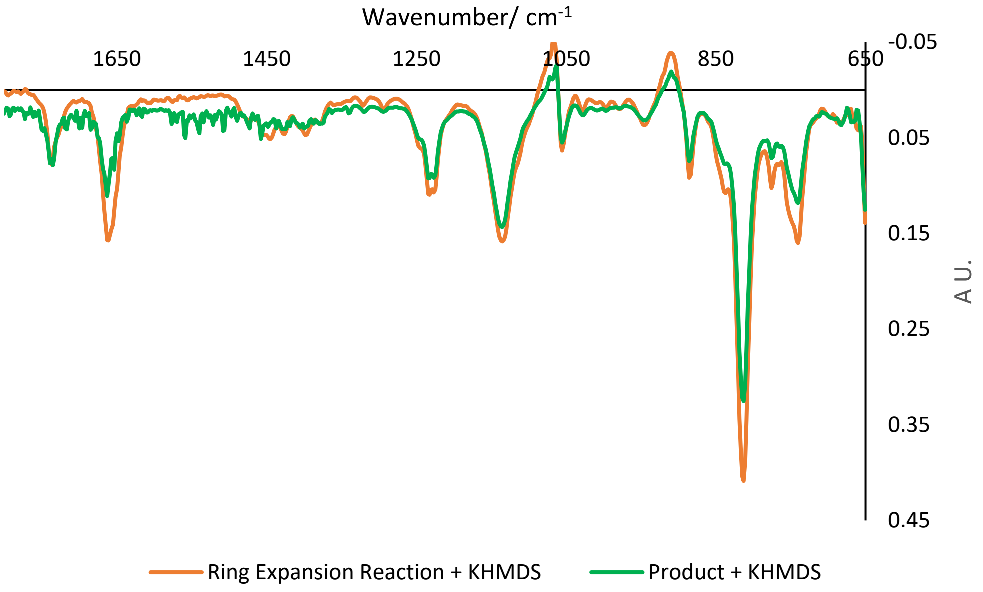

Figure S2: Graph of the ReactIR spectrum of the reaction mixture (at point **C**) of **3** with KHMDS at -5 °C in THF overlaid with the product **4** in THF treated with KHMDS forming anion **9**.

3. Structural Parameters Raw Data

Table S1: Data from X-ray crystallography. Yellow – 8-membered ring. Blue – 9-membered ring. Pink – Data excluded either atom lies in ring junction or carbonyl not imine. Orange – 10-membered ring. Green- Monocyclic hydantoins. [a] – Values for representative monocyclic hydantoins taken from refs [6-9]. [b] – DSg = Deviation from planarity = 360° - sum of bond angles. [c] – Average deviation from planarity for ring size. [d] – Dd = Change in chemical shift from the average value of 2 representative hydantoins.

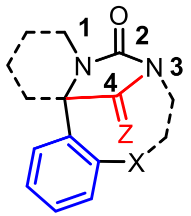

Compound Number	2a	2f	2g	2g-h	4'	4''	4'''	11a	11f	11g	18a'	18a''	19[a]	20[a]	21[a]	22[a]	23[a]
N1/°	112.06<br>123.49<br>123.66	112.06<br>123.29<br>123.29	112.72<br>123.44<br>123.83	110.16<br>119.67<br>124.02	112.60<br>122.90<br>124.30	112.10<br>122.40<br>123.00	112.30<br>121.90<br>122.25	110.83<br>123.26<br>112.53	112.67<br>126.06<br>120.95	111.94<br>123.65<br>120.70	112.54<br>122.84<br>123.09	111.41<br>121.59<br>122.74	122.71<br>122.63<br>114.05	121.48<br>122.53<br>113.91	113.45	114.80<br>120.90<br>123.90	113.60<br>123.10<br>121.60
N1 DSg[b]	0.79	1.36	0.01	6.15	0.20	2.50	3.55	13.38	0.32	3.71	1.53	4.26	0.61	2.08		0.40	1.70
Average DSg[c]	**2.08**				**2.08**						**2.89**		**1.20**				
C2/°	124.62<br>127.38<br>108.00	124.14<br>127.33<br>108.51	126.44<br>125.21<br>108.33	125.55<br>126.68<br>107.73	124.20<br>126.90<br>108.80	124.20<br>126.90<br>108.80	124.30<br>127.30<br>108.40	126.76<br>124.93<br>108.31	127.40<br>124.85<br>107.74	127.90<br>124.19<br>107.90	124.10<br>128.30<br>107.62	124.90<br>127.50<br>107.58	128.60<br>125.60<br>105.80	125.80<br>127.76<br>106.44	124.41<br>128.77<br>106.81	125.30<br>129.30<br>105.40	106.60<br>128.00<br>125.40
C2 DSg[b]	0.00	0.02	0.02	0.04	0.10	0.10	0.00	0.00	0.01	0.01	-0.02	0.02	0.00	0.00	0.01	0.00	0.00
Average DSg[c]	**0.02**				**0.04**					**0.00**			**0.00**				
^{13}C C=O (C2)/ppm	161.20	162.60	162.00	158.80	158.40			162.00	156.90	156.20	156.00		155.90	154.60			

**Average**	161.15				159.10					156.10			155.25				
**Dd[d]**	**-5.90**				**-3.85**					**-0.85**							
**N3/°**	110.19<br>117.73<br>117.92	109.07<br>117.05<br>115.57	109.17<br>115.63<br>122.92	109.55<br>118.69<br>122.95	111.20<br>122.80<br>121.00	110.80<br>120.60<br>122.70	111.00<br>122.40<br>121.60	111.29<br>123.84<br>122.00	111.51<br>123.26<br>121.21	111.06<br>126.98<br>121.21	111.42<br>124.39<br>120.65	111.44<br>123.41<br>122.18	124.85<br>111.80<br>123.04	124.36<br>123.48<br>112.13	125.81<br>122.45<br>111.69	112.60<br>127.10<br>120.10	110.90<br>124.50<br>124.50
**N3 DSg[b]**	14.16	18.31	12.28	8.81	5.00	5.90	5.00	2.87	4.02	0.75	3.54	2.97	0.31	0.03	0.05	0.20	0.10
**Average DSg[c]**	**13.39**				**4.56**					**2.42**			**0.14**				
**C4/°**	127.62<br>125.13<br>107.15	123.78<br>128.32<br>107.72	123.22<br>129.45<br>107.57	126.60<br>126.30<br>107.05	123.10<br>129.80<br>107.00	123.70<br>129.30<br>106.90	130.10<br>123.10<br>106.80	130.31<br>123.52<br>106.15	129.78<br>123.87<br>106.34	128.49<br>124.48<br>107.00	129.28<br>124.30<br>106.20	123.60<br>129.50<br>106.60	125.48<br>126.58<br>107.94	107.52<br>125.75<br>126.73	125.42<br>126.86<br>107.72	124.60<br>127.90<br>107.40	108.10<br>125.30<br>126.60
**C4 DSg[b]**	0.10	0.18	-0.24	0.05	0.10	0.10	0.00	0.02	0.01	0.03	0.22	0.30	0.00	0.00	0.00	0.10	0.00
**Average DSg[c]**	**0.02**				**0.05**					**0.18**			**0.02**				
**^{13}C C=NH (C4)/ppm**	172.00	173.90	173.40	182.30	172.10			172.70	172.20	170.70	167.40		173.90	173.40			
**Average**	173.10				172.33					169.05			173.65				
**Dd[d]**	**0.55**				**1.32**					**4.60**							
**Dihedral Angle 1234/°**	1.79	3.95	5.63	2.99	0.48	0.39	1.50	10.61	3.86	9.82	5.74	5.87	6.14	1.52	0.22	1.67	2.80

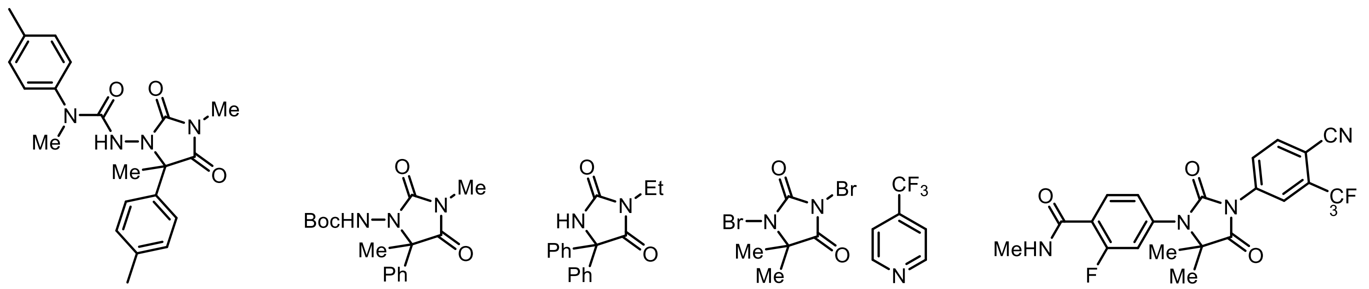

19 **20** **21** **22** **23**

Figure S3: Literature monocyclic hydantoins used in Table 1.[6–9]

4. ^{1}H and ^{13}C NMR Spectra Products

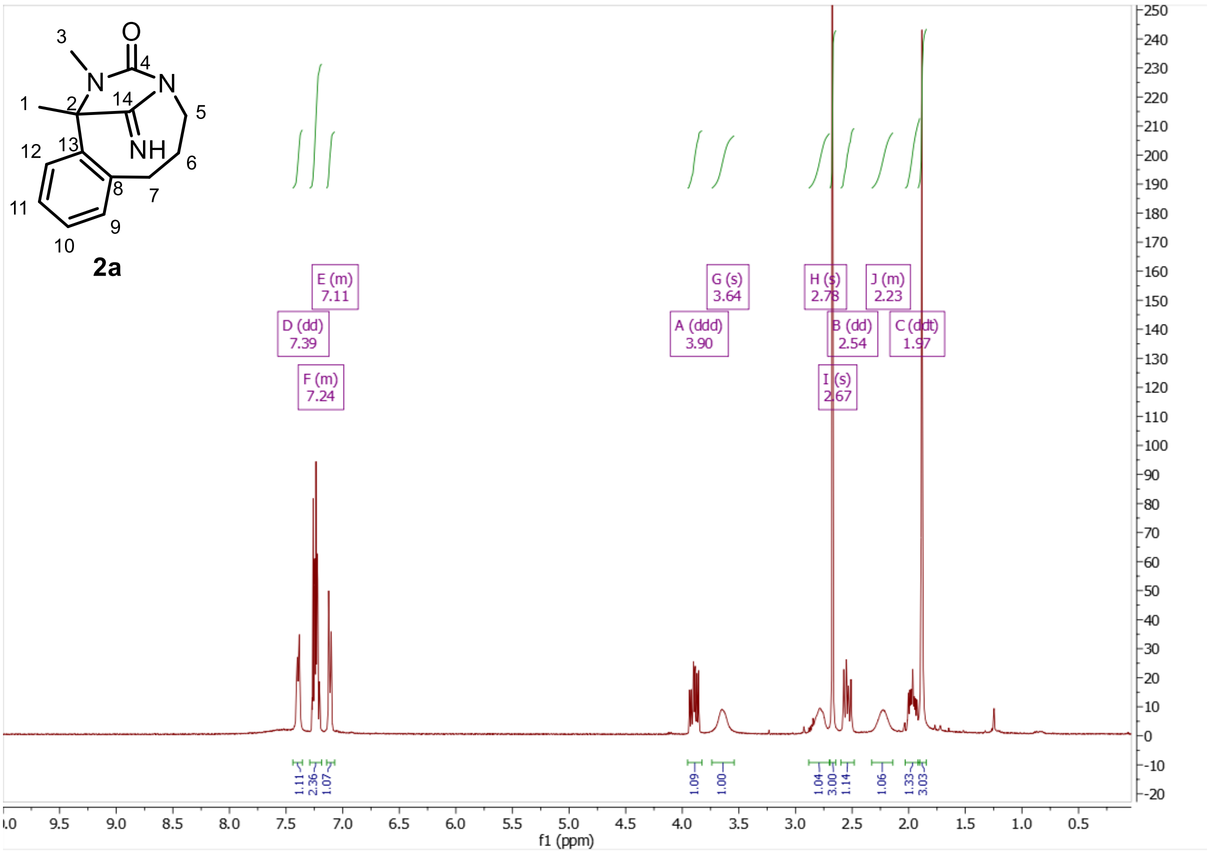

2a

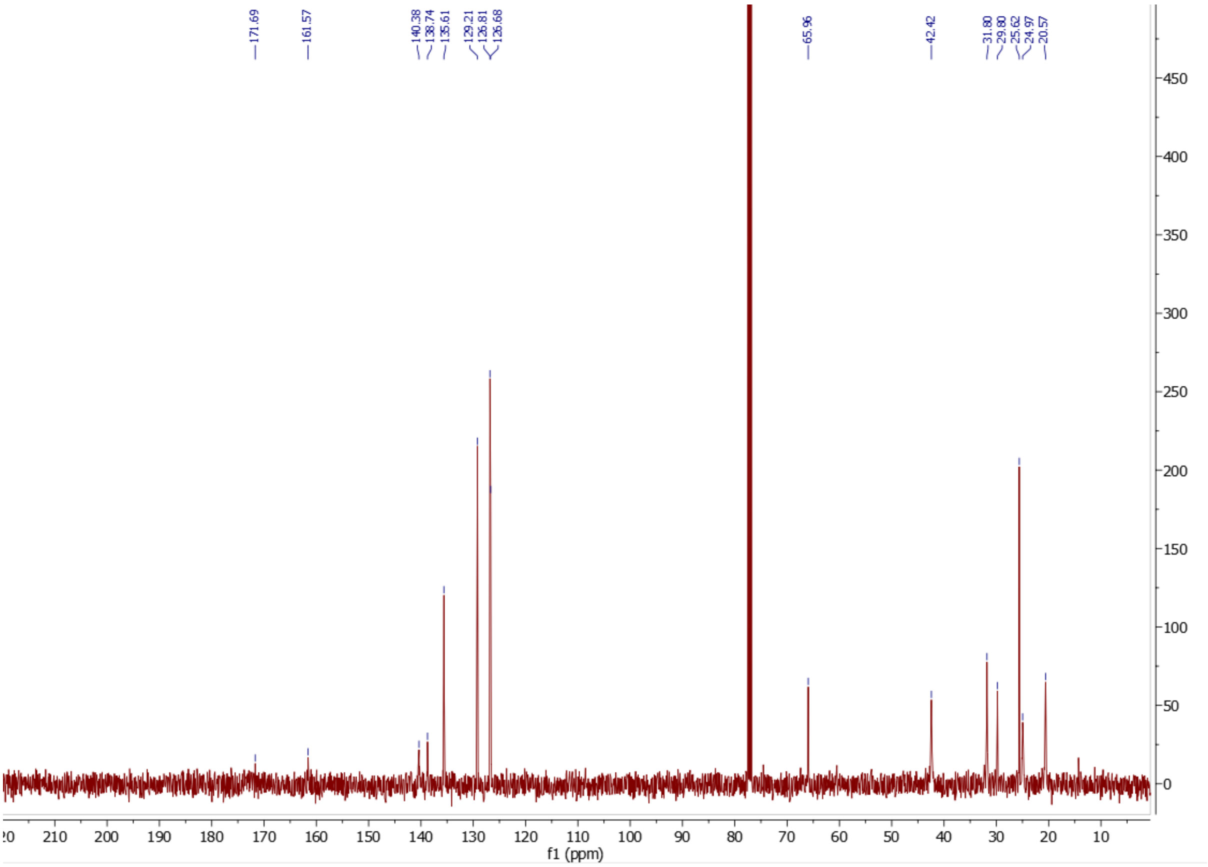

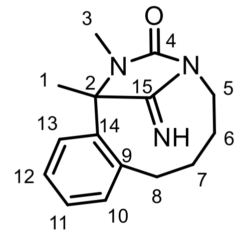

3
O
4
N
N
1
2
15
5
13
14
NH
6
9
12
7
8
11
10

4

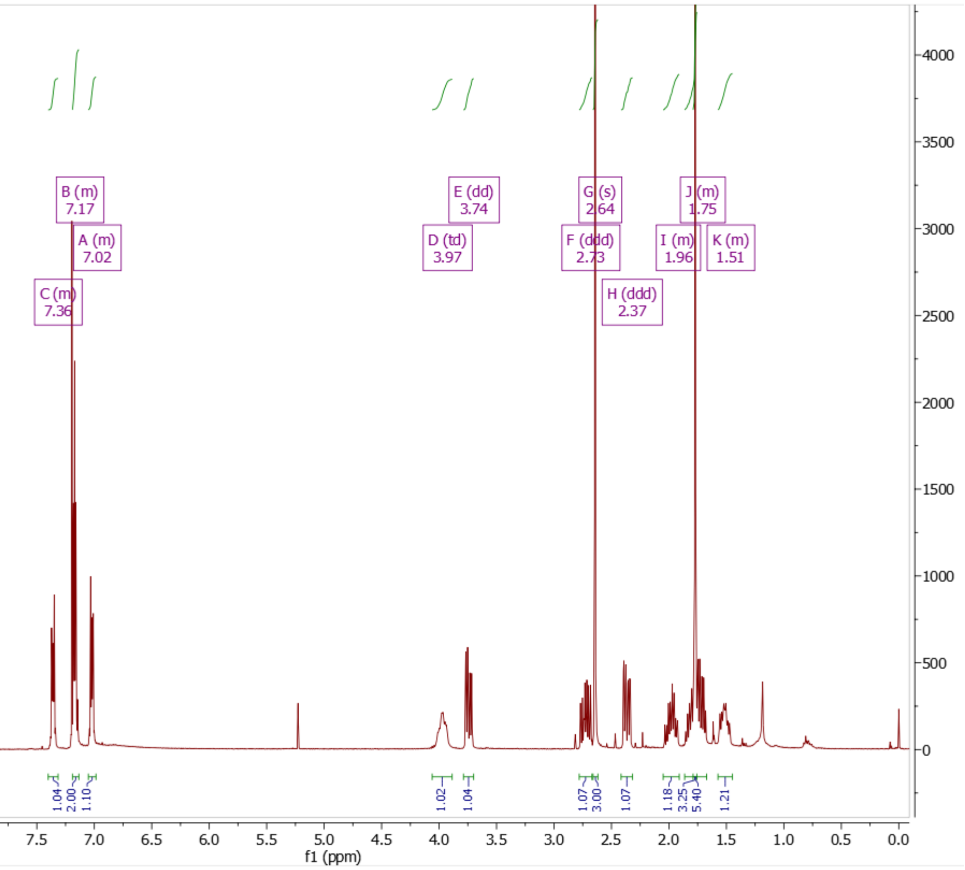

B (m) 7.17
A (m) 7.02
C (m) 7.36
E (dd) 3.74
D (td) 3.97
G (s) 2.64
F (ddd) 2.73
H (ddd) 2.37
J (m) 1.75
I (m) 1.96
K (m) 1.51
1.04
2.00
1.10
1.02
1.04
1.07
3.00
1.07
1.18
3.25
5.40
1.21
f1 (ppm)

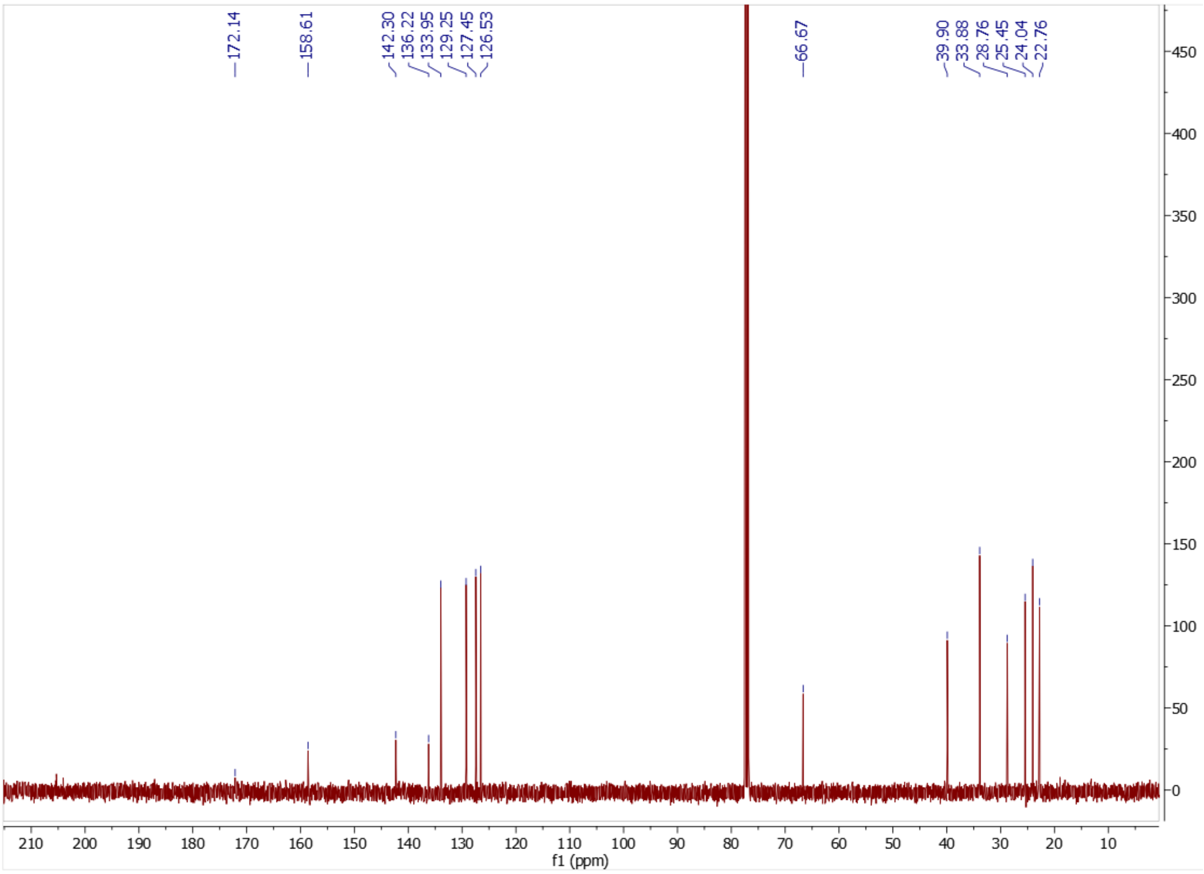

172.14
158.61
142.30
136.22
133.95
129.25
127.45
126.53
66.67
39.90
33.88
28.76
25.45
24.04
22.76
f1 (ppm)

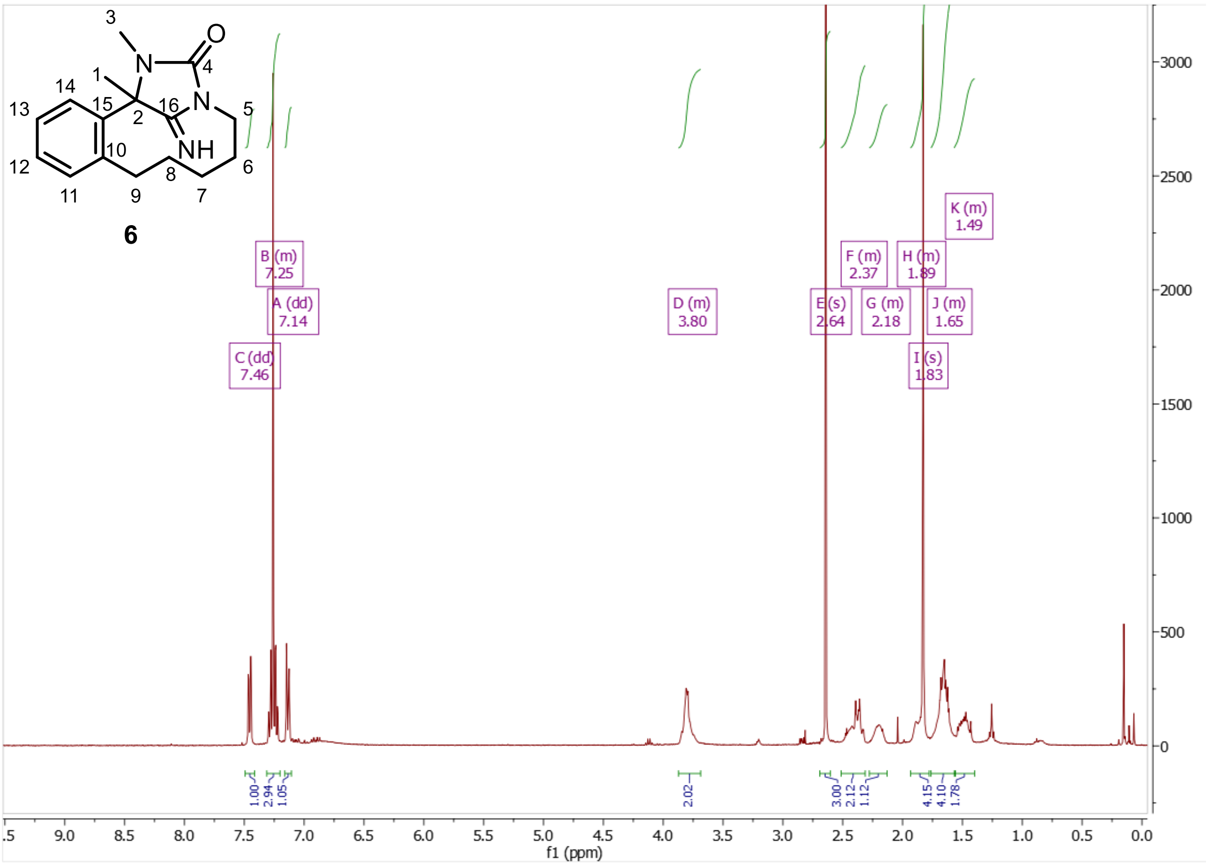

6
C (dd) 7.46
B (m) 7.25
A (dd) 7.14
D (m) 3.80
E (s) 2.64
F (m) 2.37
G (m) 2.18
H (m) 1.89
I (s) 1.83
J (m) 1.65
K (m) 1.49
1.00
2.94
1.05
2.02
3.00
2.12
1.12
4.15
4.10
1.78
f1 (ppm)

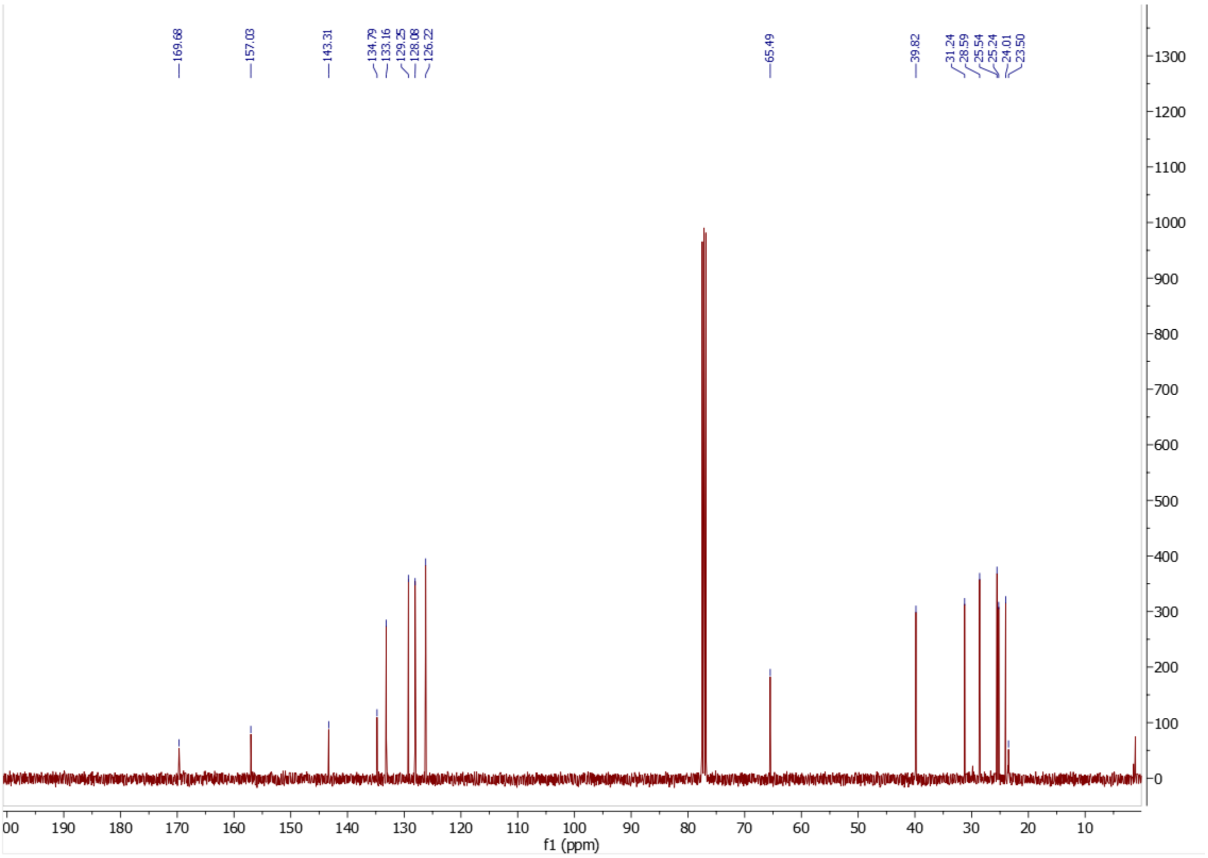

169.68
157.03
143.31
134.79
133.16
129.25
128.08
126.22
65.49
39.82
31.24
28.59
25.54
25.24
24.01
23.50
f1 (ppm)

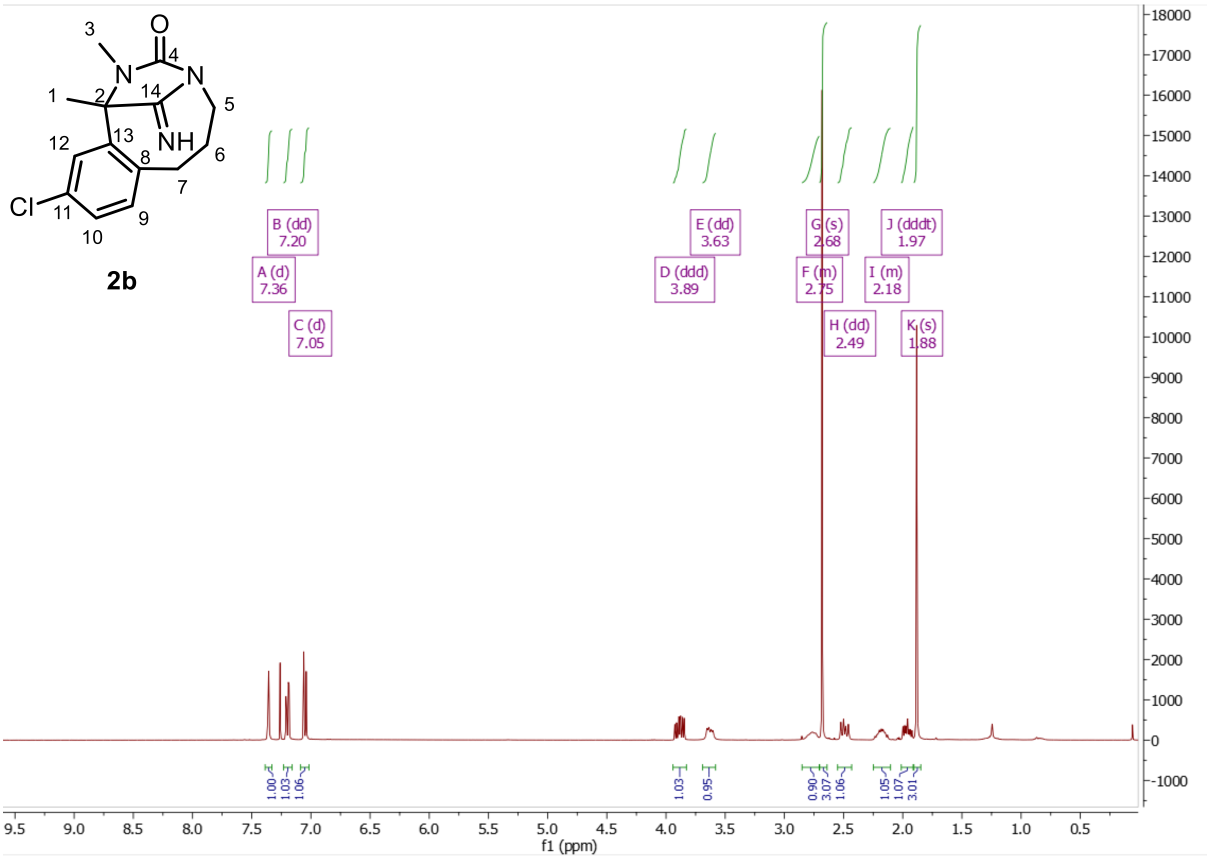
3
O
4
N
N
1
2
14
5
13
NH
12
8
6
7
Cl
11
9
10
2b
A (d)
7.36
B (dd)
7.20
C (d)
7.05
D (ddd)
3.89
E (dd)
3.63
F (m)
2.75
G (s)
2.68
H (dd)
2.49
I (m)
2.18
J (dddt)
1.97
K (s)
1.88
1.00
1.03
1.06
1.03
0.95
0.90
3.07
1.06
1.05
1.07
3.01
f1 (ppm)

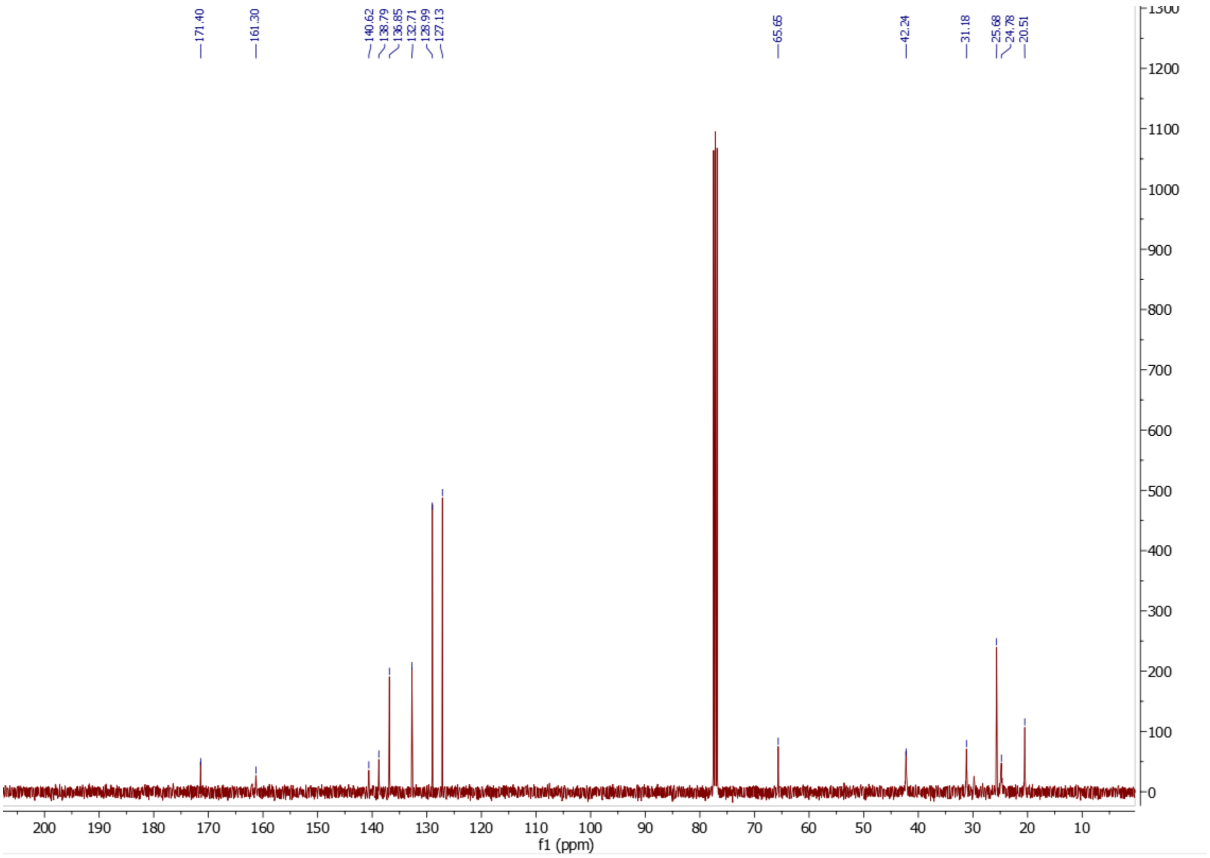
171.40
161.30
140.62
138.79
136.85
132.71
128.99
127.13
65.65
42.24
31.18
25.68
24.78
20.51
f1 (ppm)

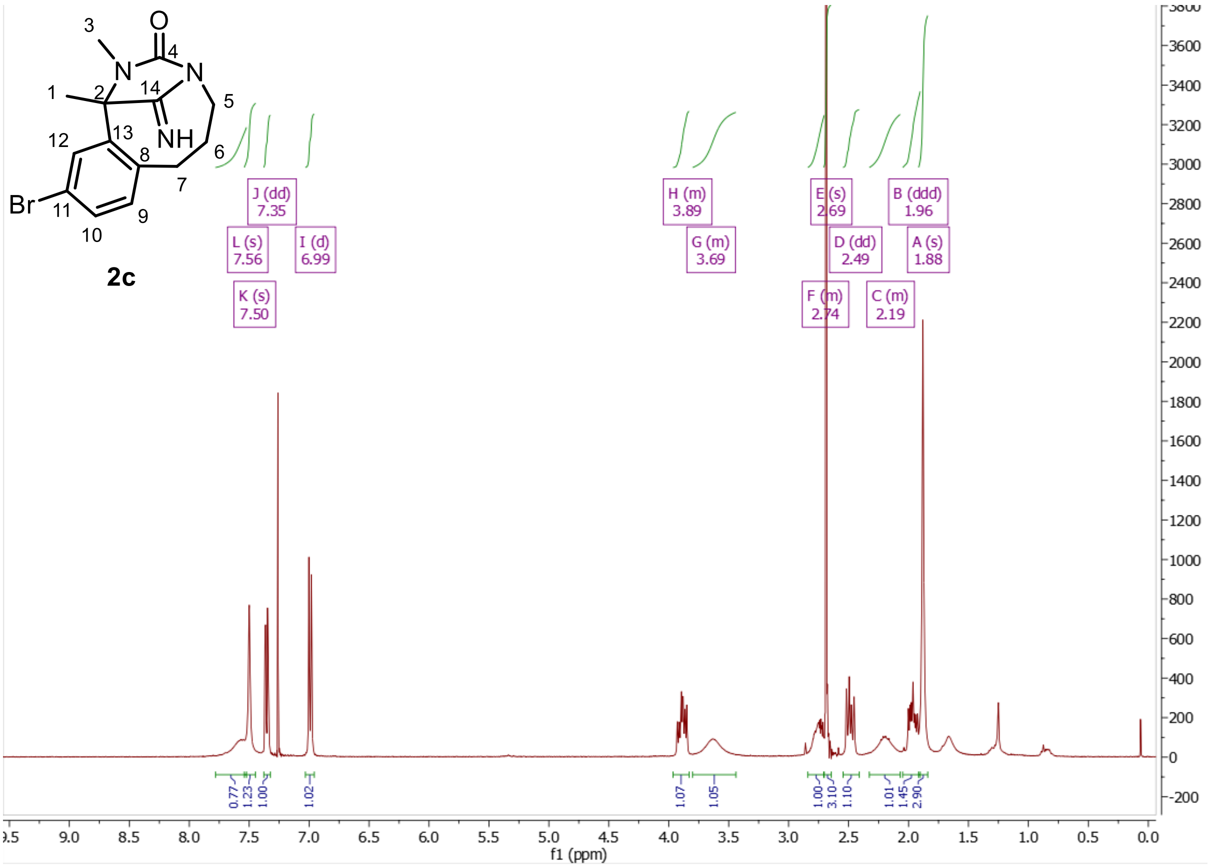
Br
NH
2c
L (s) 7.56
K (s) 7.50
J (dd) 7.35
I (d) 6.99
H (m) 3.89
G (m) 3.69
F (m) 2.74
E (s) 2.69
D (dd) 2.49
C (m) 2.19
B (ddd) 1.96
A (s) 1.88
f1 (ppm)

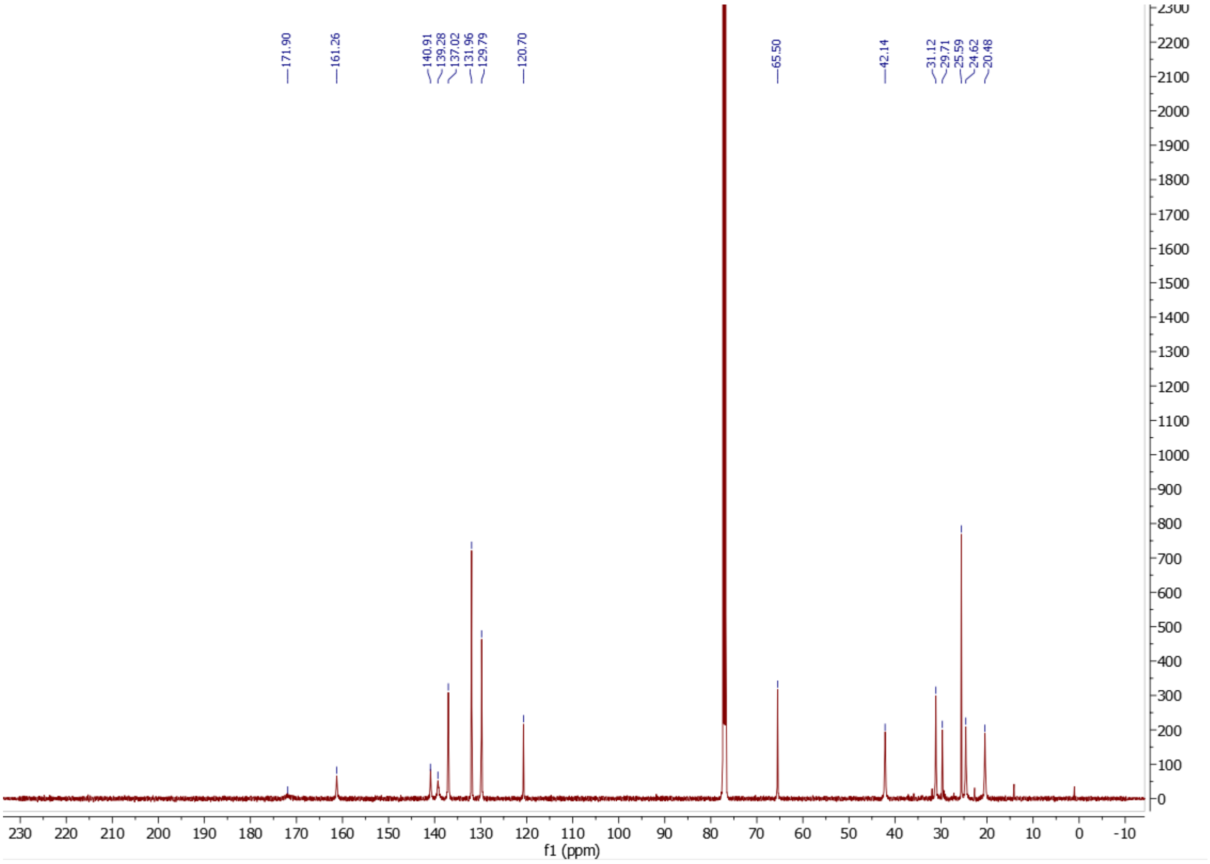
171.90
161.26
140.91
139.28
137.02
131.96
129.79
120.70
65.50
42.14
31.12
29.71
25.59
24.62
20.48
f1 (ppm)

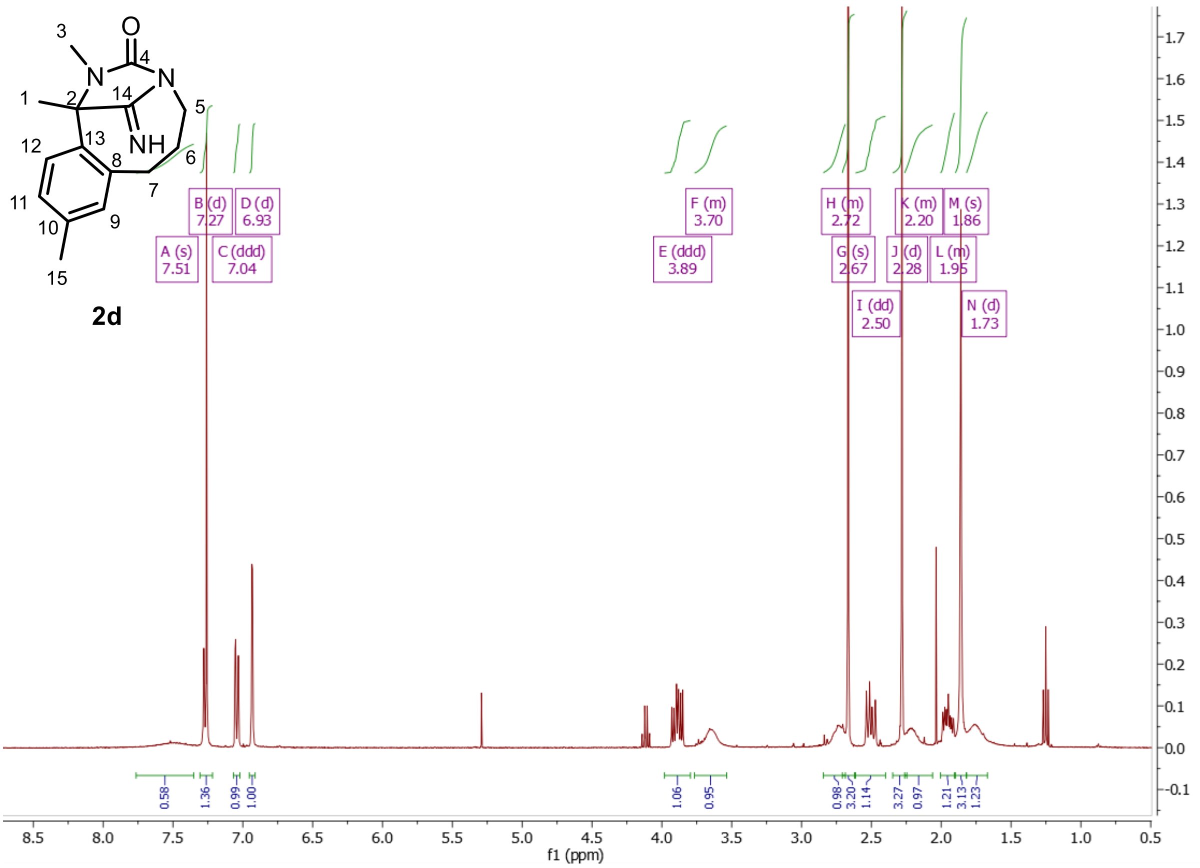

2d

A (s)
7.51
B (d)
7.27
C (ddd)
7.04
D (d)
6.93
E (ddd)
3.89
F (m)
3.70
G (s)
2.67
H (m)
2.72
I (dd)
2.50
J (d)
2.28
K (m)
2.20
L (m)
1.95
M (s)
1.86
N (d)
1.73
0.58
1.36
0.99
1.00
1.06
0.95
0.98
3.20
1.14
3.27
0.97
1.21
3.13
1.23
f1 (ppm)

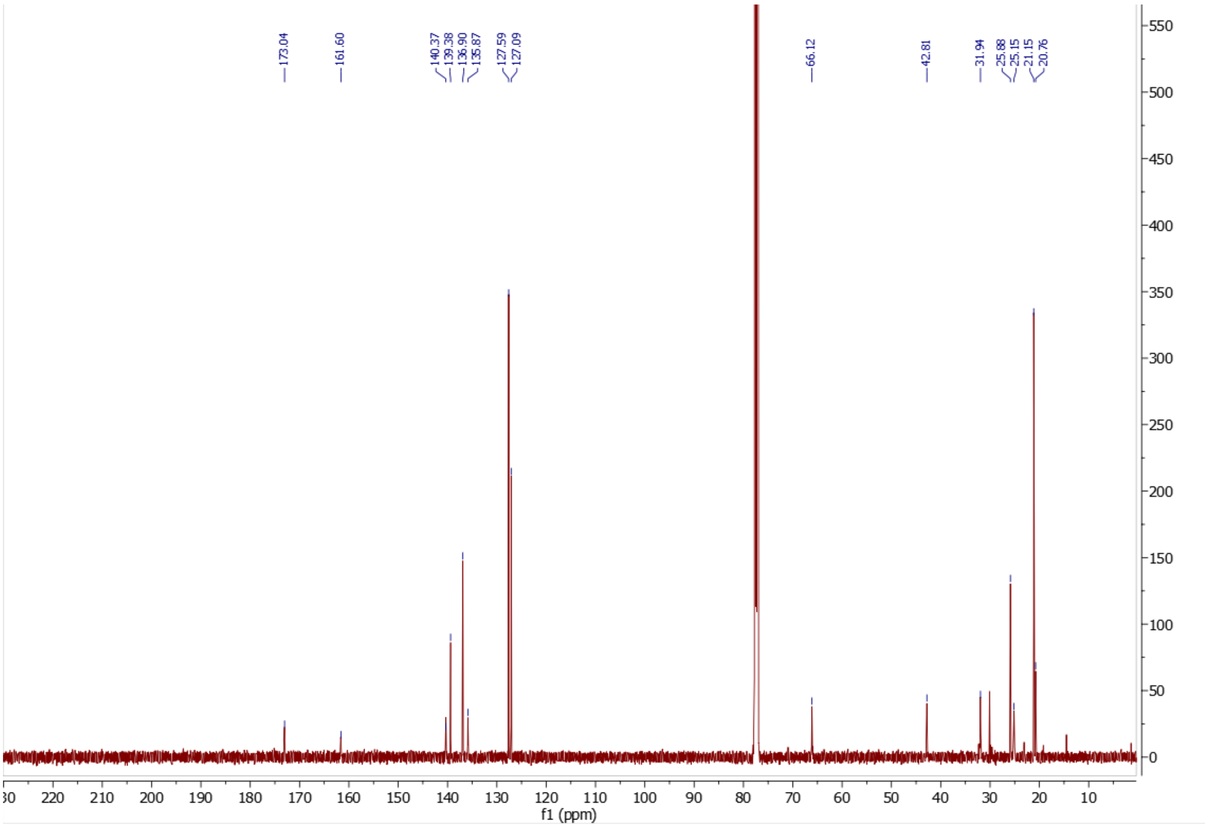
173.04
161.60
140.37
139.38
136.90
135.87
127.59
127.09
66.12
42.81
31.94
25.88
25.15
21.15
20.76
f1 (ppm)

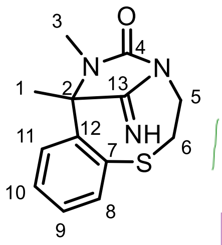
3
O
4
N
N
1
2
13
5
11
12
NH
7
6
S
10
8
9

2e

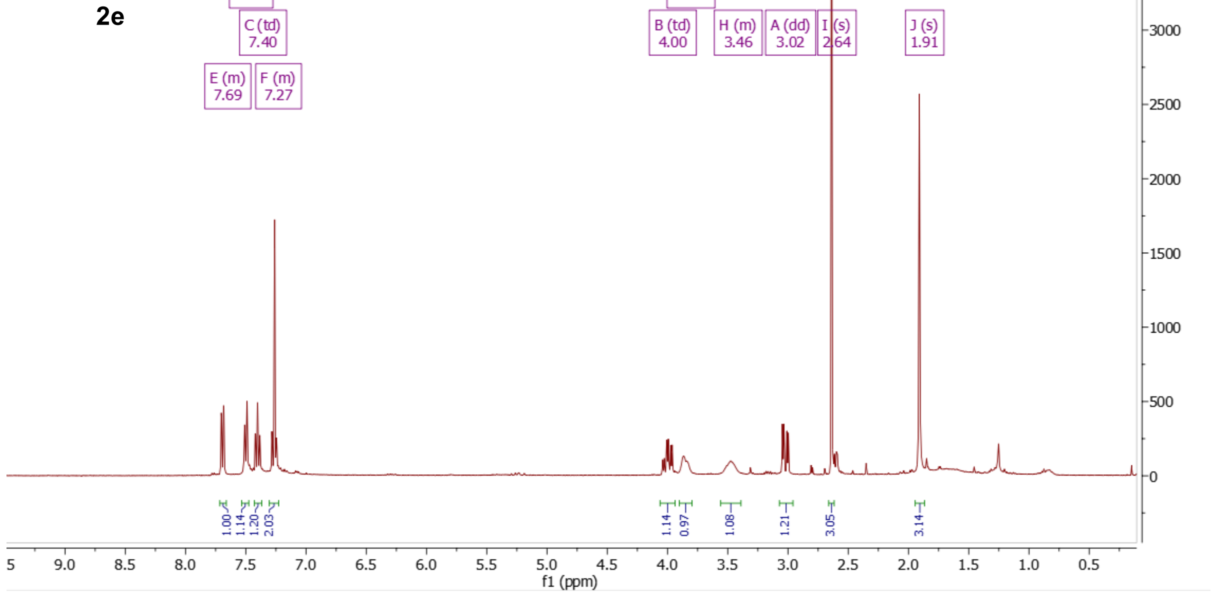
D (d)
7.50
C (td)
7.40
E (m)
7.69
F (m)
7.27
G (m)
3.85
B (td)
4.00
H (m)
3.46
A (dd)
3.02
I (s)
2.64
J (s)
1.91
1.00
1.14
1.20
2.03
1.14
0.97
1.08
1.21
3.05
3.14
f1 (ppm)

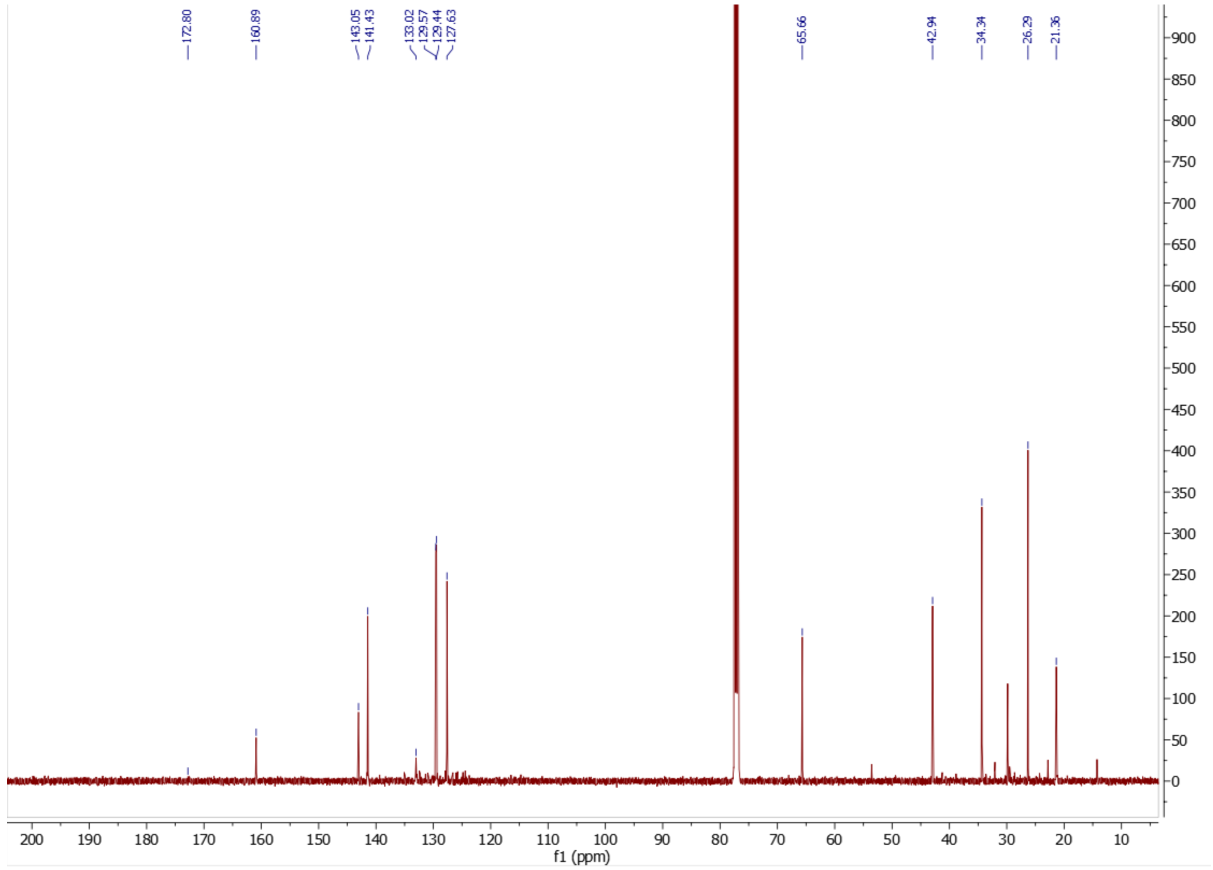
172.80
160.89
143.05
141.43
133.02
129.57
129.44
127.63
65.66
42.94
34.34
26.29
21.36
f1 (ppm)

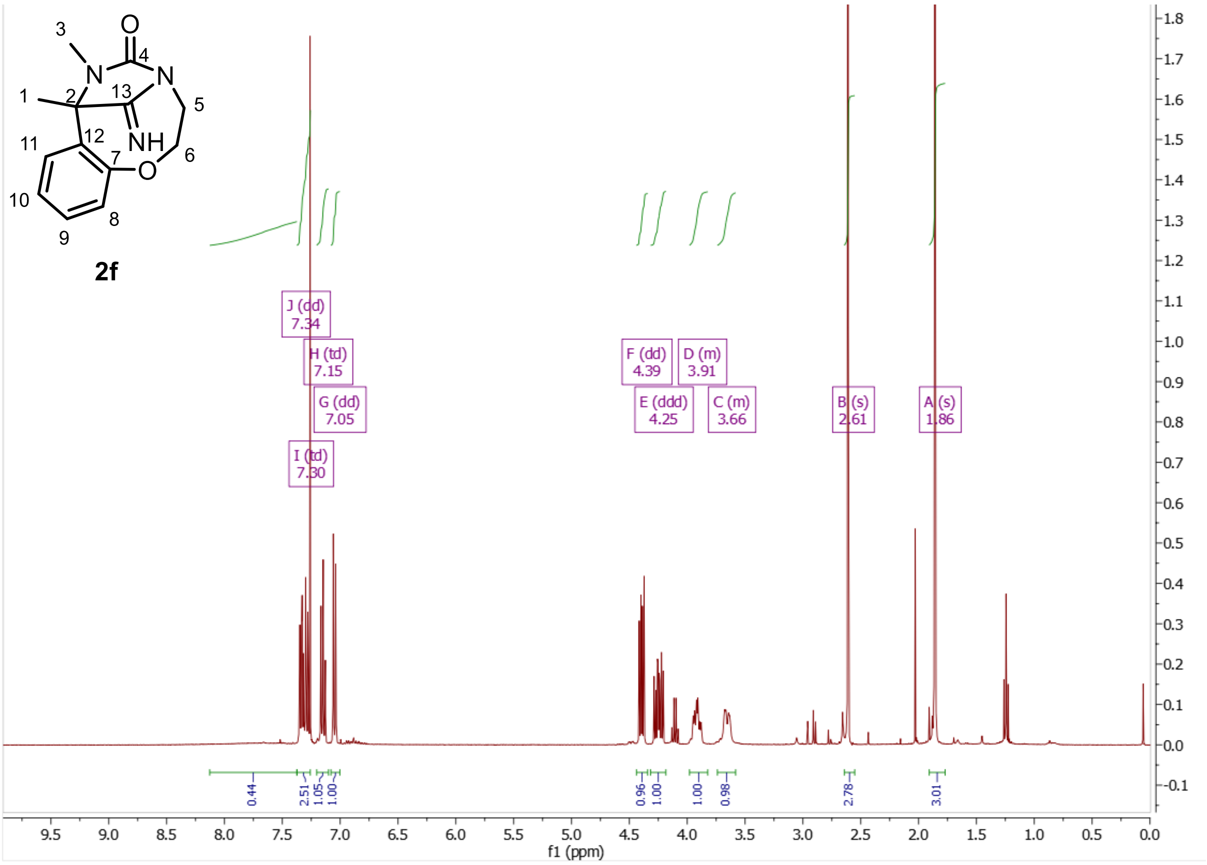
3
4
1
2
13
N
N
O
5
11
12
NH
6
7
O
10
8
9
2f
J (dd)
7.34
H (td)
7.15
G (dd)
7.05
I (td)
7.30
F (dd)
4.39
D (m)
3.91
E (ddd)
4.25
C (m)
3.66
B (s)
2.61
A (s)
1.86
0.44
2.51
1.05
1.00
0.96
1.00
1.00
0.98
2.78
3.01
f1 (ppm)

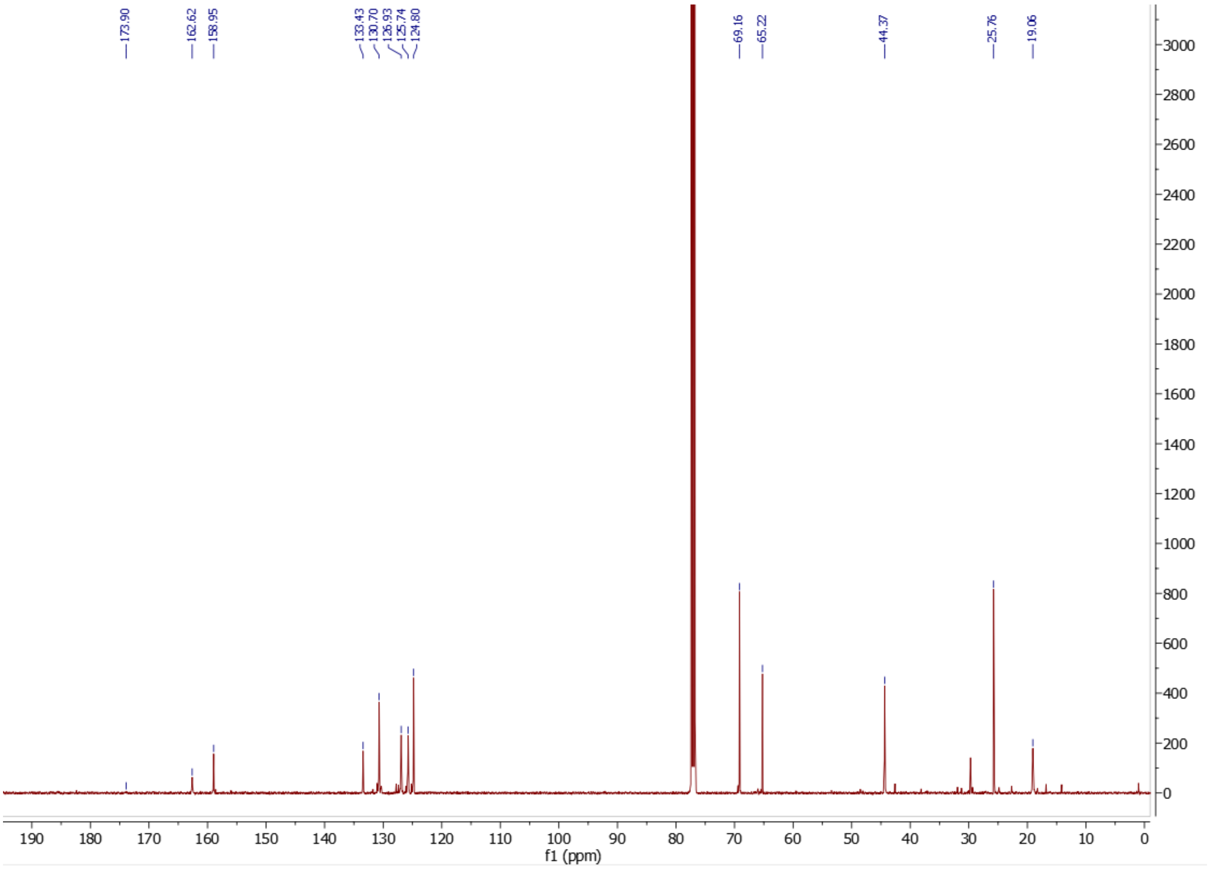
173.90
162.62
158.95
133.43
130.70
126.93
125.74
124.80
69.16
65.22
44.37
25.76
19.06
f1 (ppm)

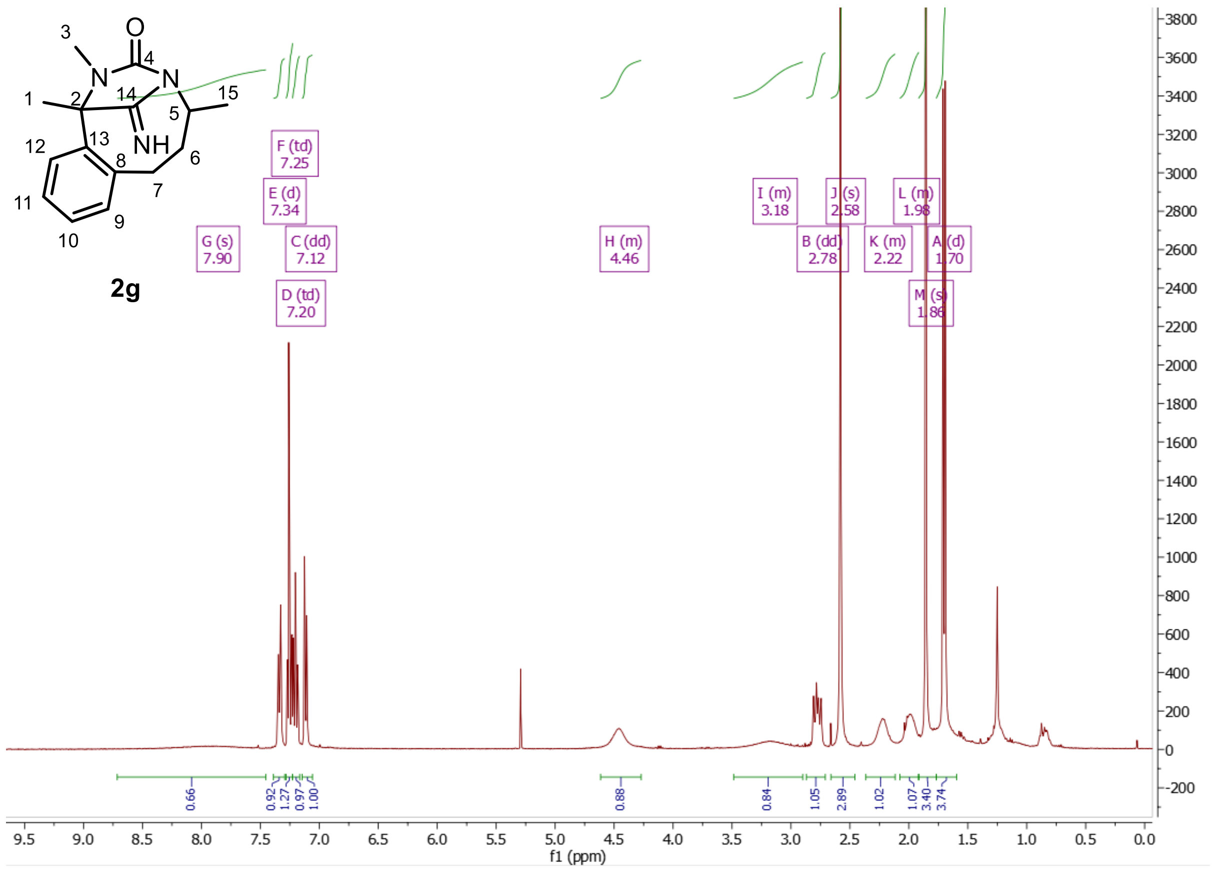

2g

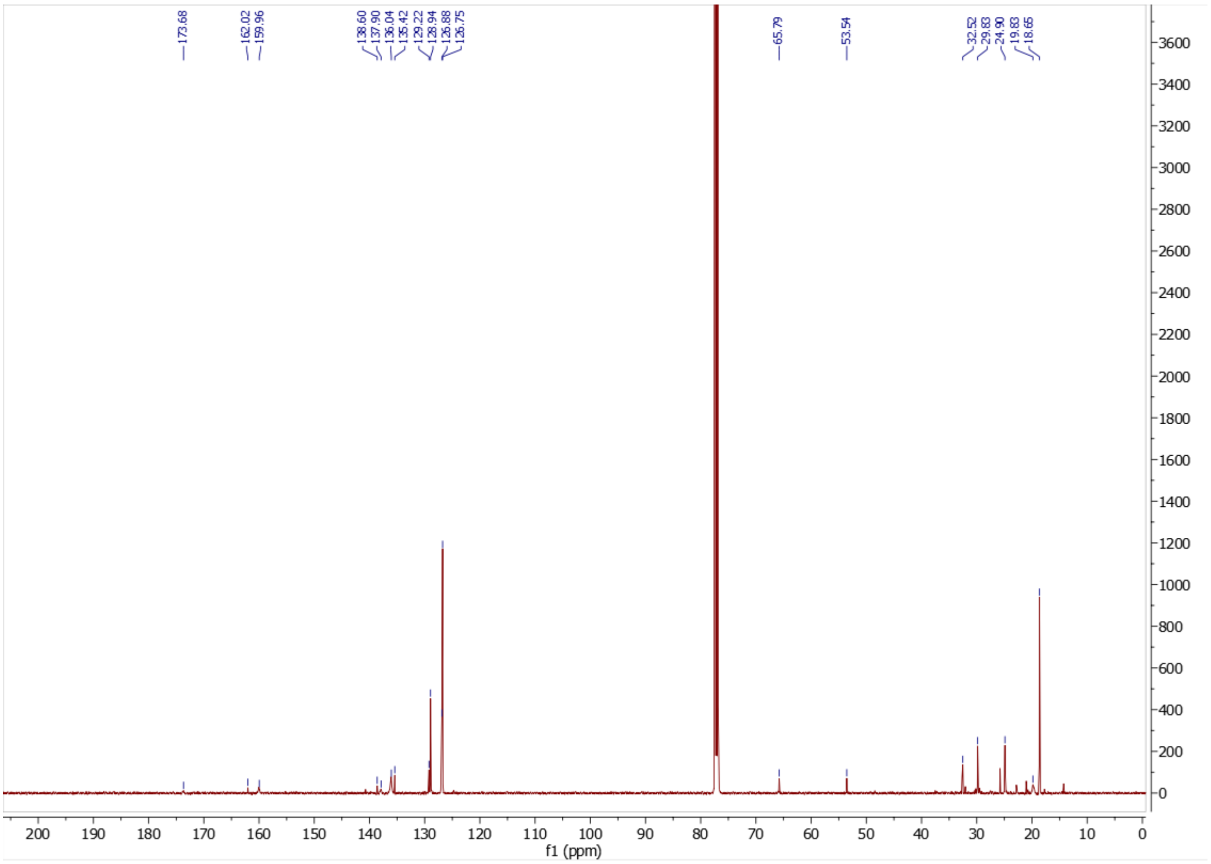

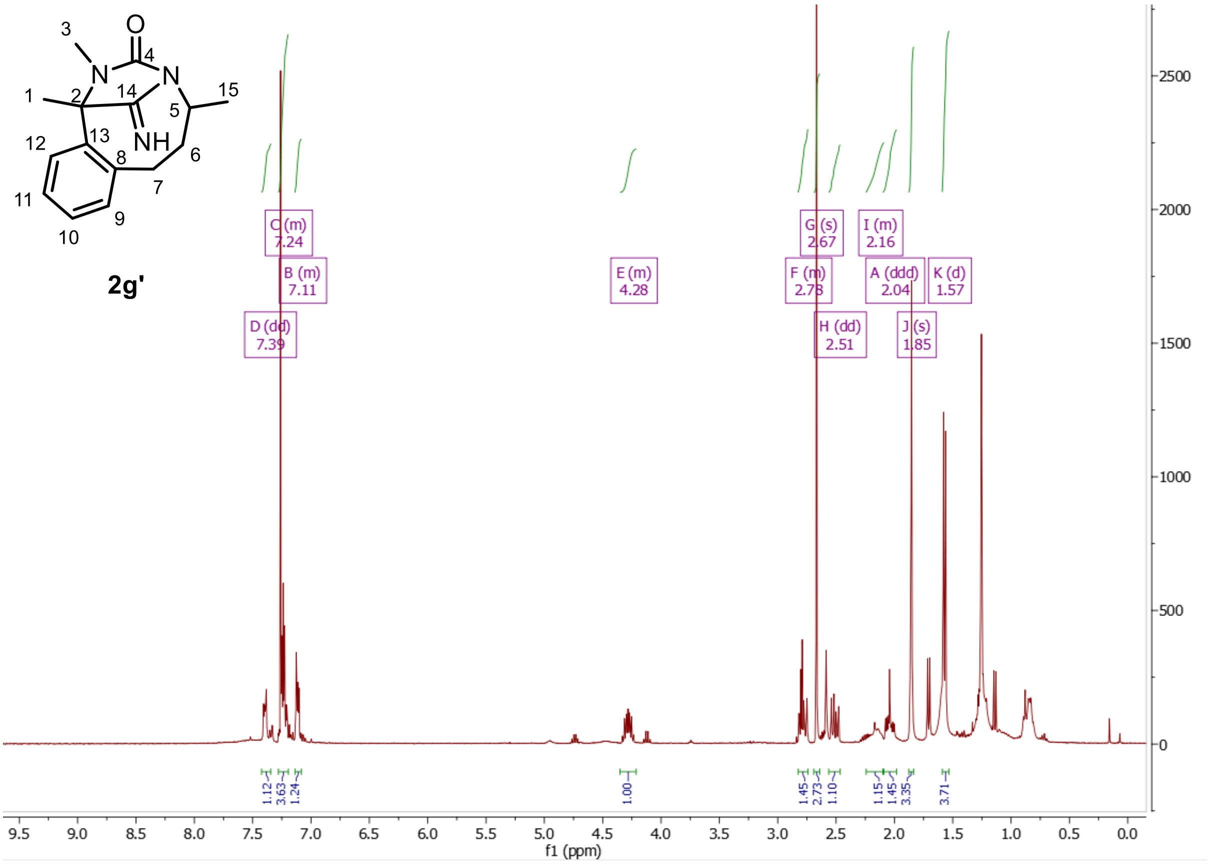

O
N
N
NH
1
2
3
4
5
6
7
8
9
10
11
12
13
14
15
2g'
C (m) 7.24
B (m) 7.11
D (dd) 7.39
E (m) 4.28
G (s) 2.67
F (m) 2.78
H (dd) 2.51
I (m) 2.16
A (ddd) 2.04
J (s) 1.85
K (d) 1.57
1.12
3.63
1.24
1.00
1.45
2.73
1.10
1.15
1.45
3.35
3.71
f1 (ppm)

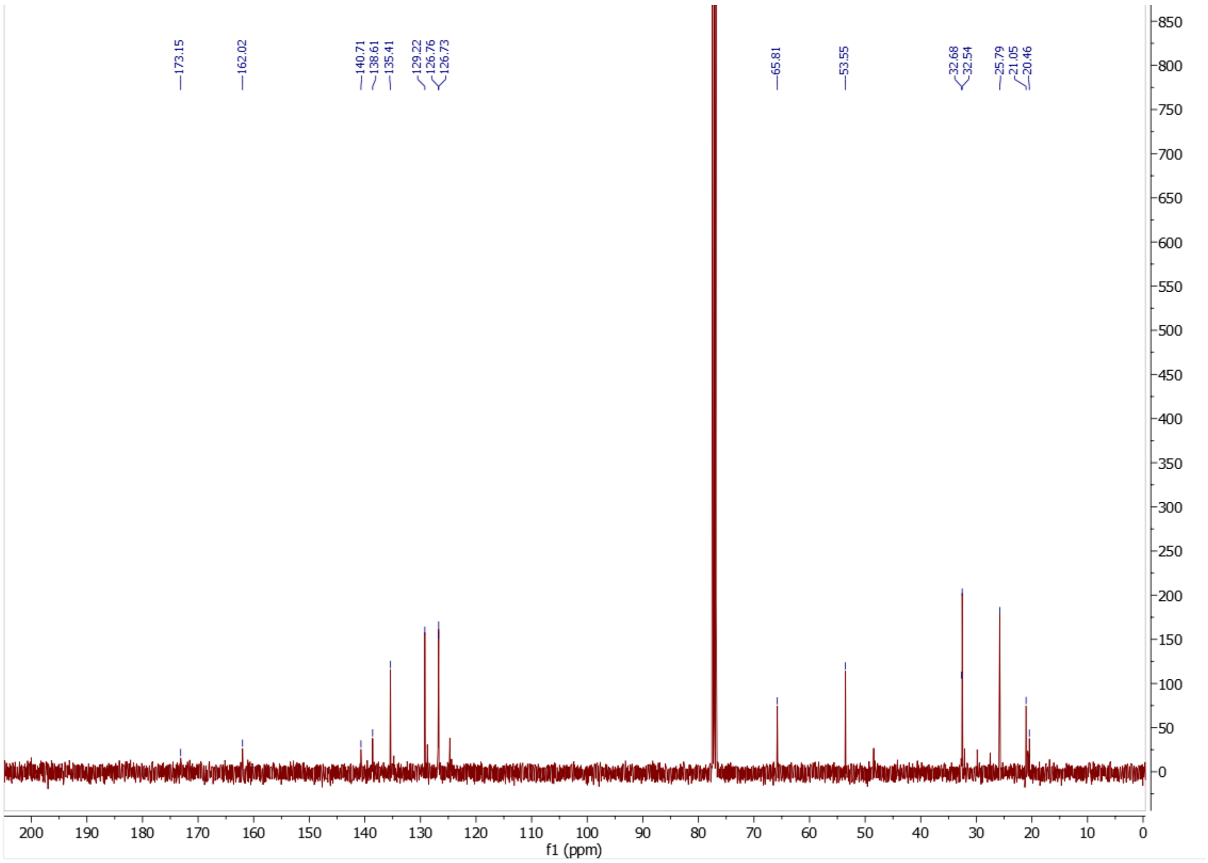

173.15
162.02
140.71
138.61
135.41
129.22
126.76
126.73
65.81
53.55
32.68
32.54
25.79
21.05
20.46
f1 (ppm)

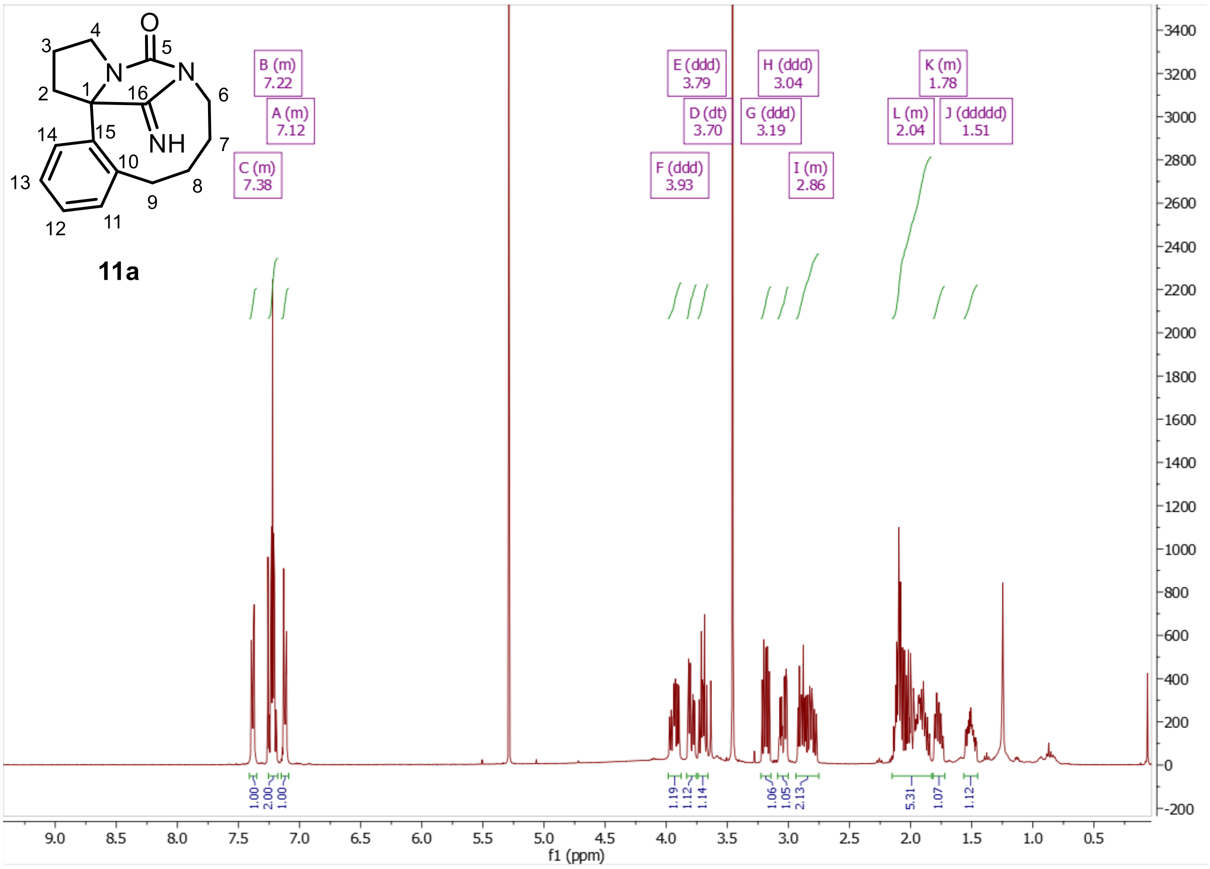
11a
B (m) 7.22
A (m) 7.12
C (m) 7.38
E (ddd) 3.79
D (dt) 3.70
F (ddd) 3.93
H (ddd) 3.04
G (ddd) 3.19
I (m) 2.86
K (m) 1.78
L (m) 2.04
J (ddddd) 1.51
f1 (ppm)

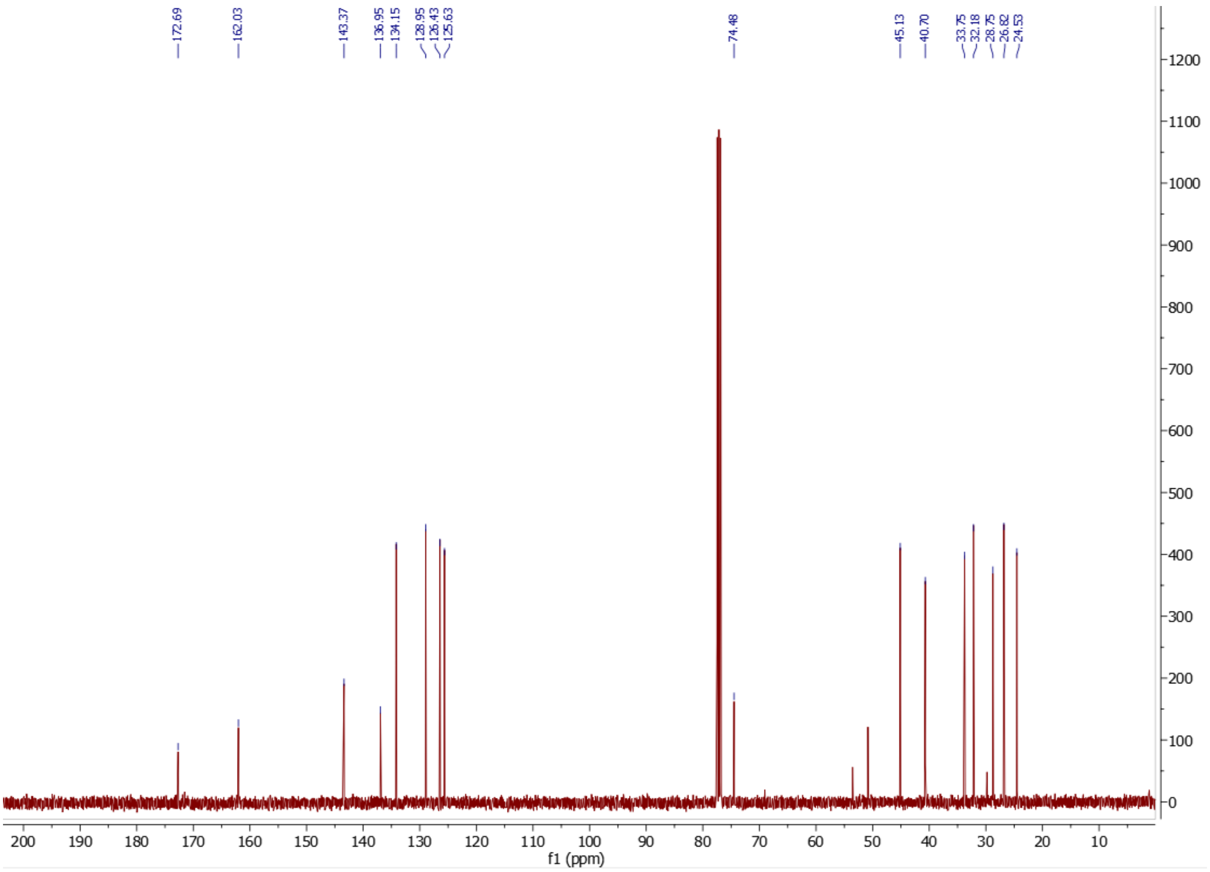
172.69
162.03
143.37
136.95
134.15
128.95
126.43
125.63
74.48
45.13
40.70
33.75
32.18
28.75
26.82
24.53
f1 (ppm)

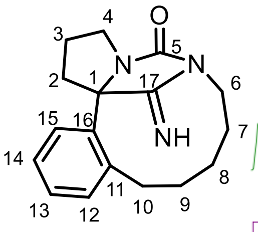

O
N
N
NH
1
2
3
4
5
6
7
8
9
10
11
12
13
14
15
16
17

11b

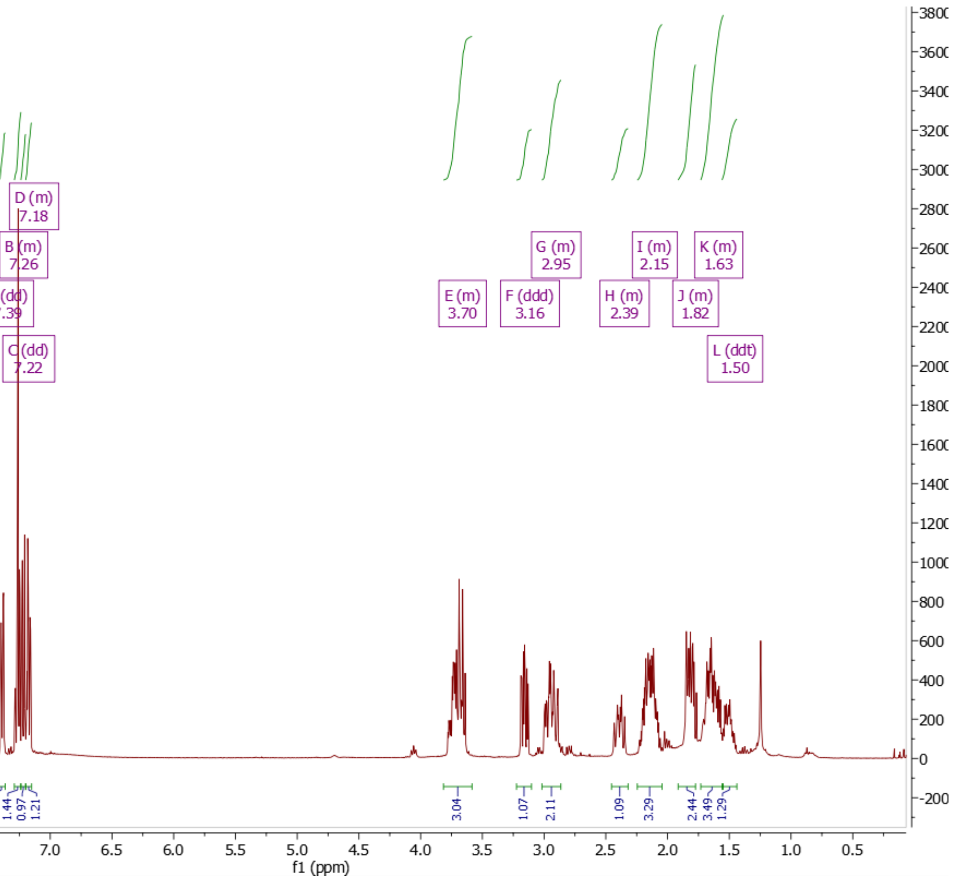

A (dd) 7.39
B (m) 7.26
C (dd) 7.22
D (m) 7.18
E (m) 3.70
F (ddd) 3.16
G (m) 2.95
H (m) 2.39
I (m) 2.15
J (m) 1.82
K (m) 1.63
L (ddt) 1.50
1.00
1.44
0.97
1.21
3.04
1.07
2.11
1.09
3.29
2.44
3.49
1.29
f1 (ppm)

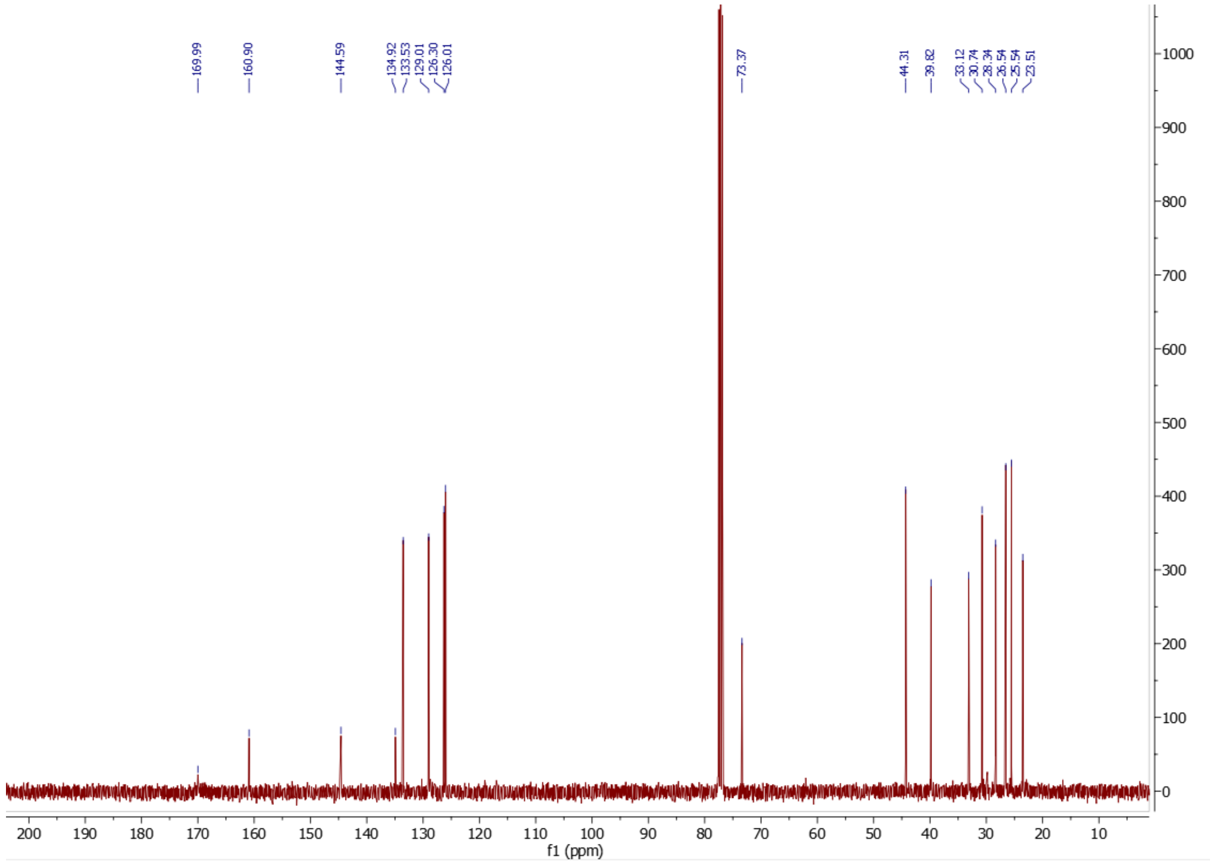

169.99
160.90
144.59
134.92
133.53
129.01
126.30
126.01
73.37
44.31
39.82
33.12
30.74
28.34
26.54
25.54
23.51
f1 (ppm)

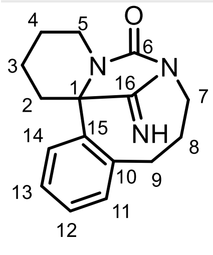
11c

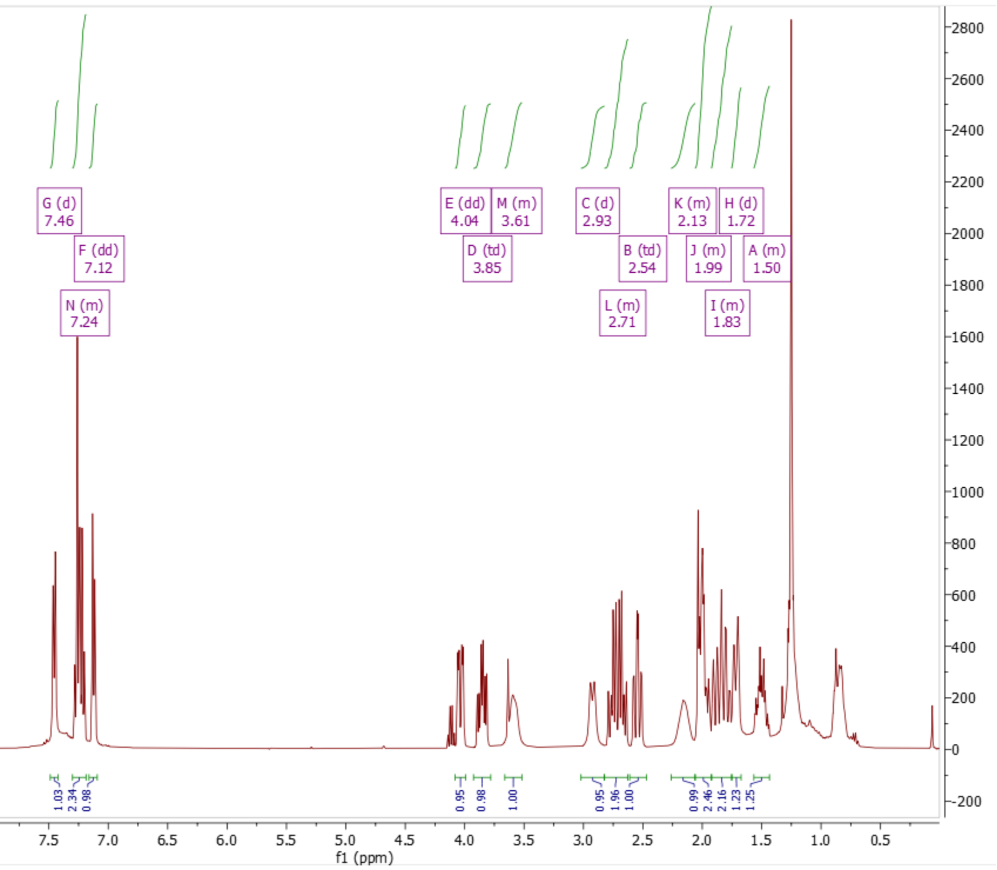
G (d) 7.46
F (dd) 7.12
N (m) 7.24
E (dd) 4.04
M (m) 3.61
D (td) 3.85
C (d) 2.93
B (td) 2.54
L (m) 2.71
K (m) 2.13
H (d) 1.72
J (m) 1.99
A (m) 1.50
I (m) 1.83
f1 (ppm)

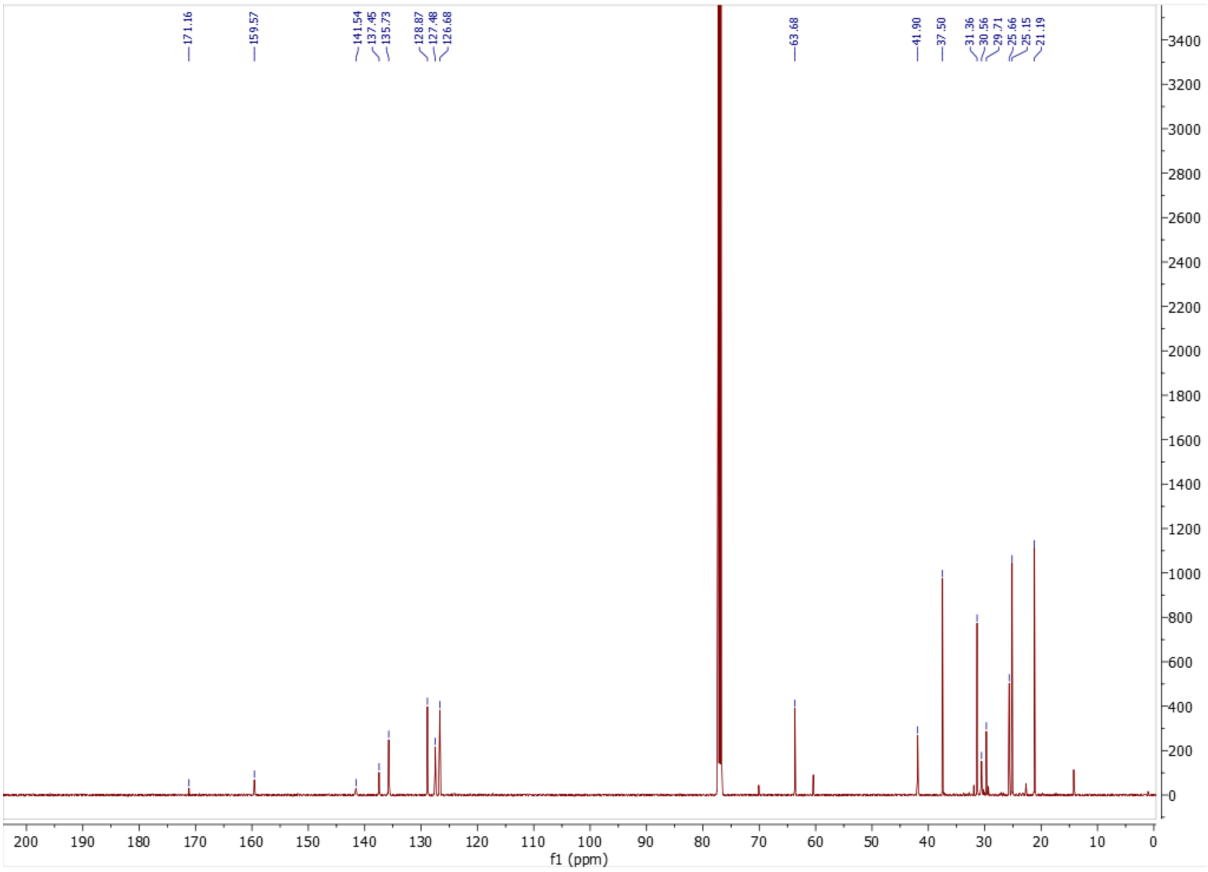
171.16
159.57
141.54
137.45
135.73
128.87
127.48
126.68
63.68
41.90
37.50
31.36
30.56
29.71
25.66
25.15
21.19
f1 (ppm)

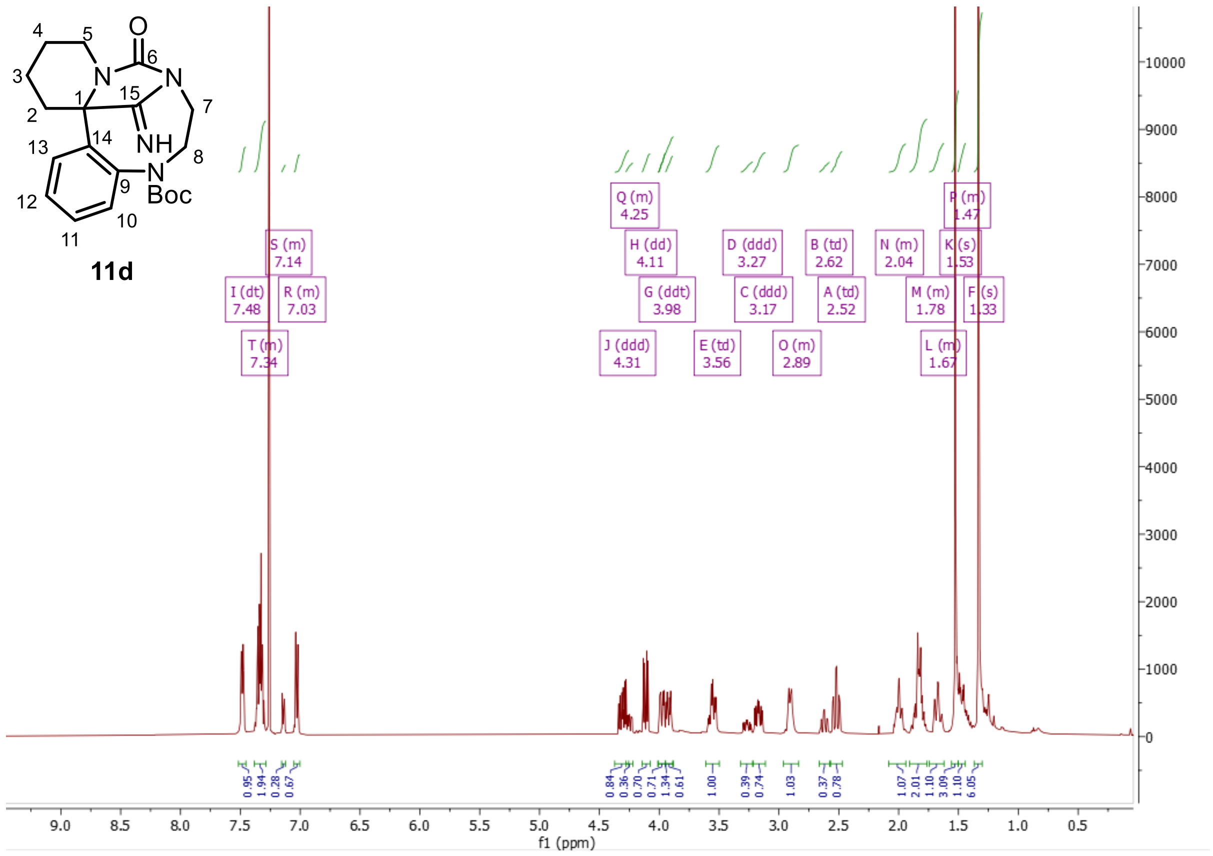
O
N
NH
N
Boc
11d
Q (m) 4.25
S (m) 7.14
H (dd) 4.11
D (ddd) 3.27
B (td) 2.62
N (m) 2.04
K (s) 1.53
P (m) 1.47
I (dt) 7.48
R (m) 7.03
G (ddt) 3.98
C (ddd) 3.17
A (td) 2.52
M (m) 1.78
F (s) 1.33
T (m) 7.34
J (ddd) 4.31
E (td) 3.56
O (m) 2.89
L (m) 1.67
0.95 1.94 0.28 0.67
0.84 0.36 0.70 0.71 1.34 0.61
1.00 0.39 0.74 1.03 0.37 0.78
1.07 2.01 1.10 3.09 1.10 6.05
f1 (ppm)

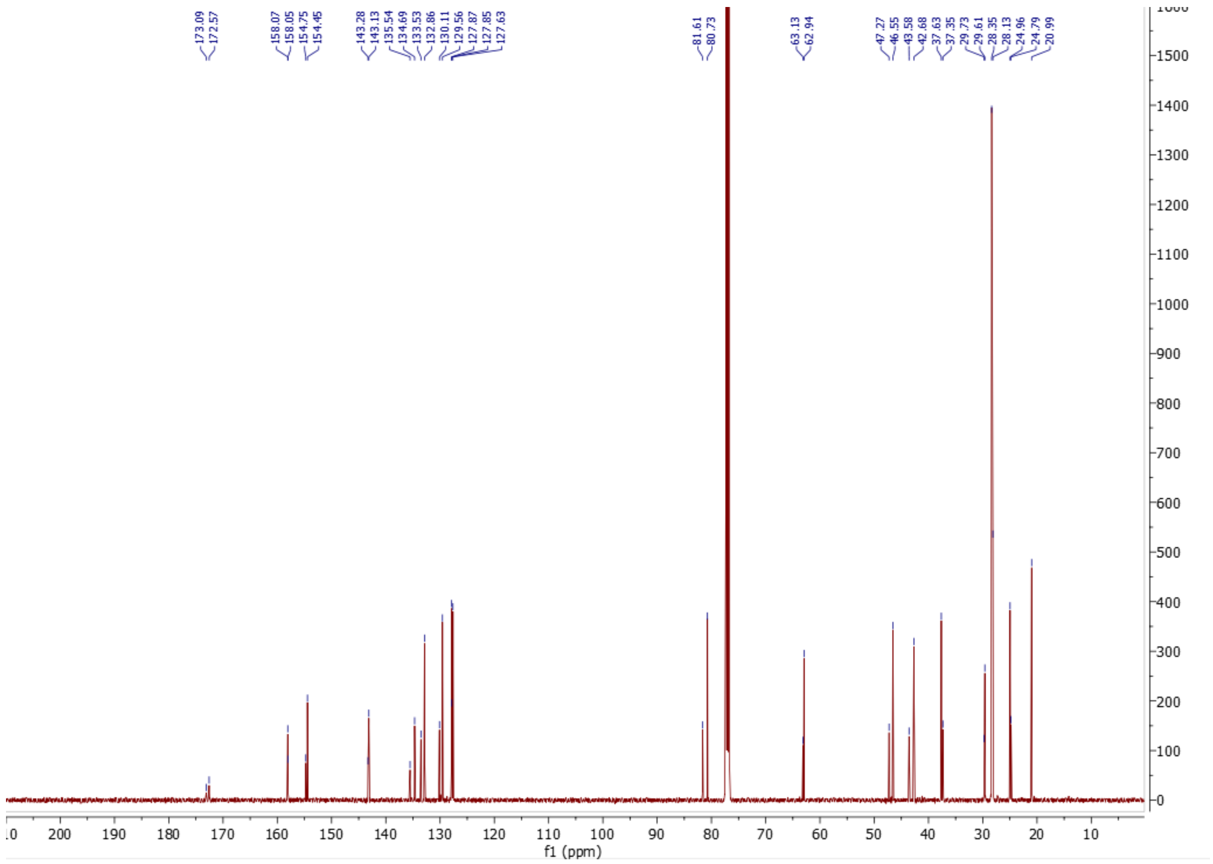
173.09
172.57
158.07
158.05
154.75
154.45
143.28
143.13
135.54
134.69
133.53
132.86
130.11
129.56
127.87
127.85
127.63
81.61
80.73
63.13
62.94
47.27
46.55
43.58
42.68
37.63
37.35
29.73
29.61
28.35
28.13
24.96
24.79
20.99
f1 (ppm)

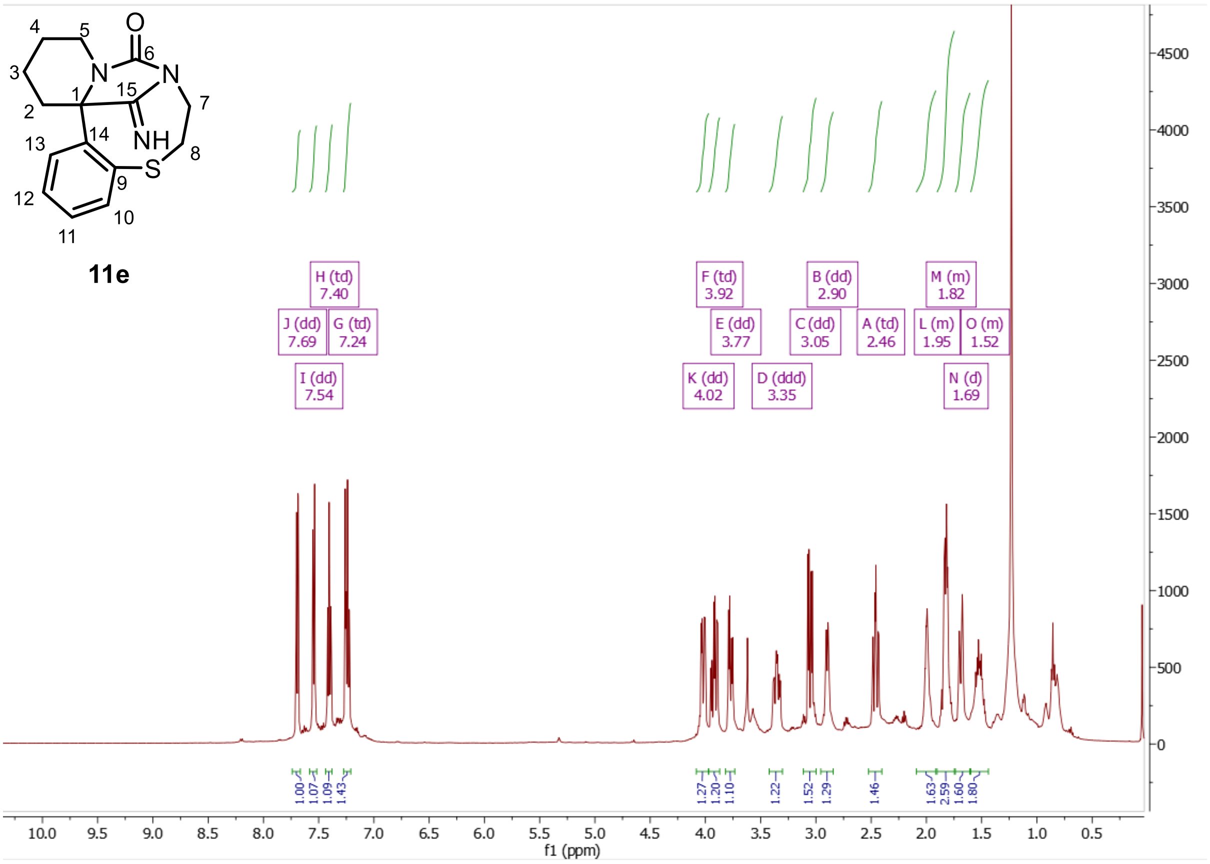
11e
H (td) 7.40
J (dd) 7.69
G (td) 7.24
I (dd) 7.54
F (td) 3.92
E (dd) 3.77
K (dd) 4.02
D (ddd) 3.35
B (dd) 2.90
C (dd) 3.05
A (td) 2.46
M (m) 1.82
L (m) 1.95
O (m) 1.52
N (d) 1.69
1.00 1.07 1.09 1.43 1.27 1.20 1.10 1.22 1.52 1.29 1.46 1.63 2.59 1.60 1.80
f1 (ppm)

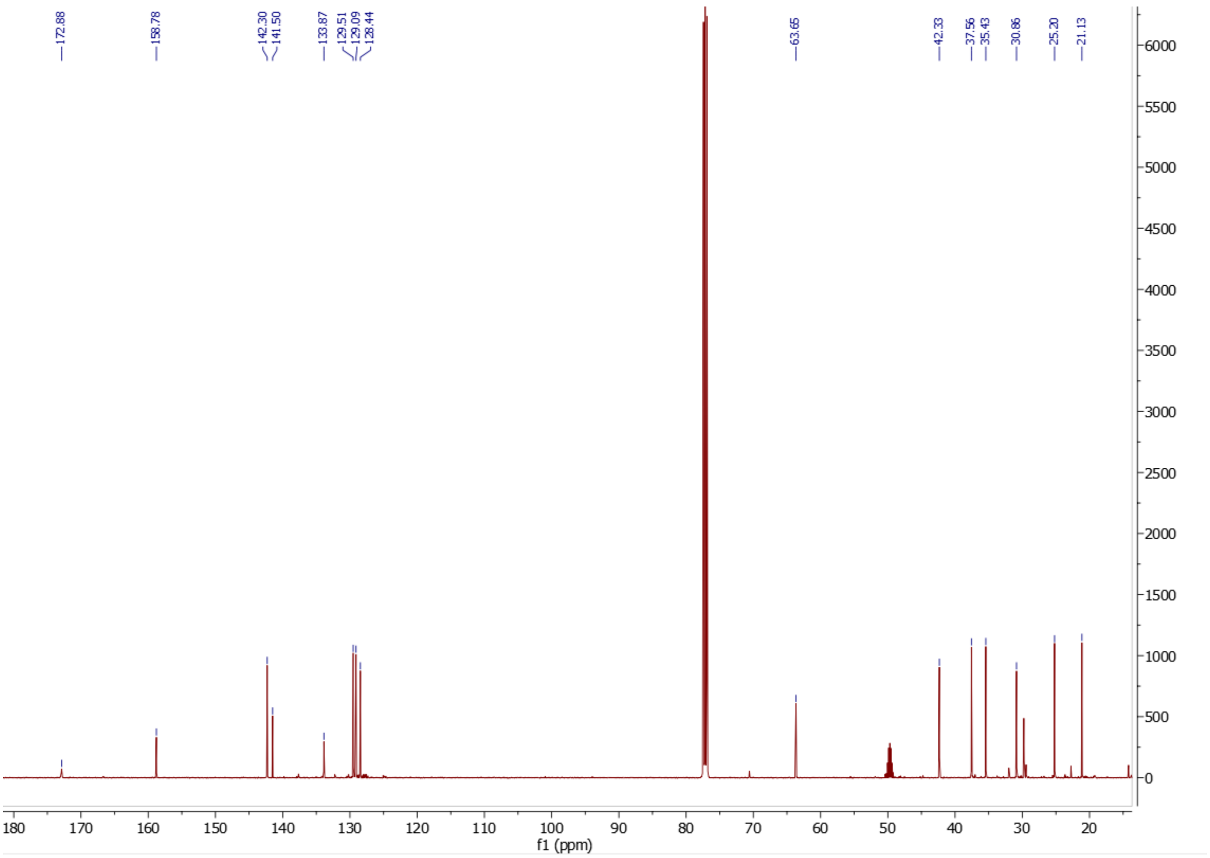
172.88
158.78
142.30
141.50
133.87
129.51
129.09
128.44
63.65
42.33
37.56
35.43
30.86
25.20
21.13
f1 (ppm)

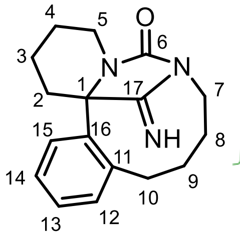
O
NH
N
N
1
2
3
4
5
6
7
8
9
10
11
12
13
14
15
16
17

11f

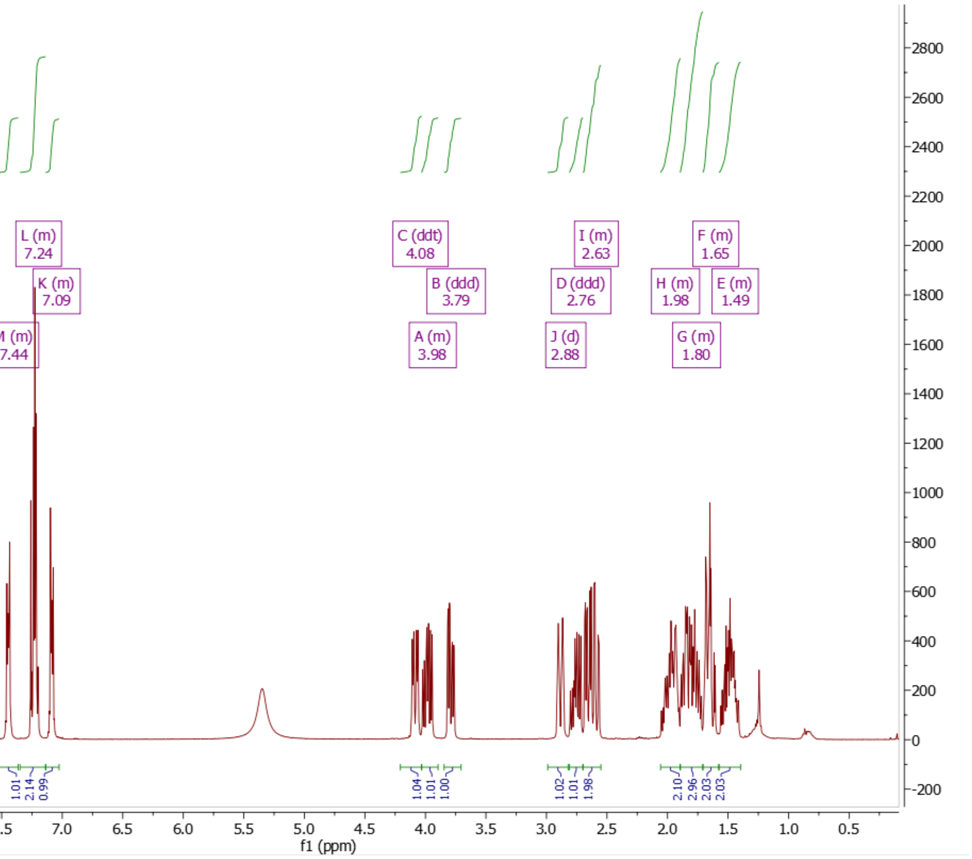
M (m) 7.44
L (m) 7.24
K (m) 7.09
C (ddt) 4.08
A (m) 3.98
B (ddd) 3.79
J (d) 2.88
D (ddd) 2.76
I (m) 2.63
H (m) 1.98
G (m) 1.80
F (m) 1.65
E (m) 1.49
1.01
2.14
0.99
1.04
1.01
1.00
1.02
1.01
1.98
2.10
2.96
2.03
2.03
f1 (ppm)

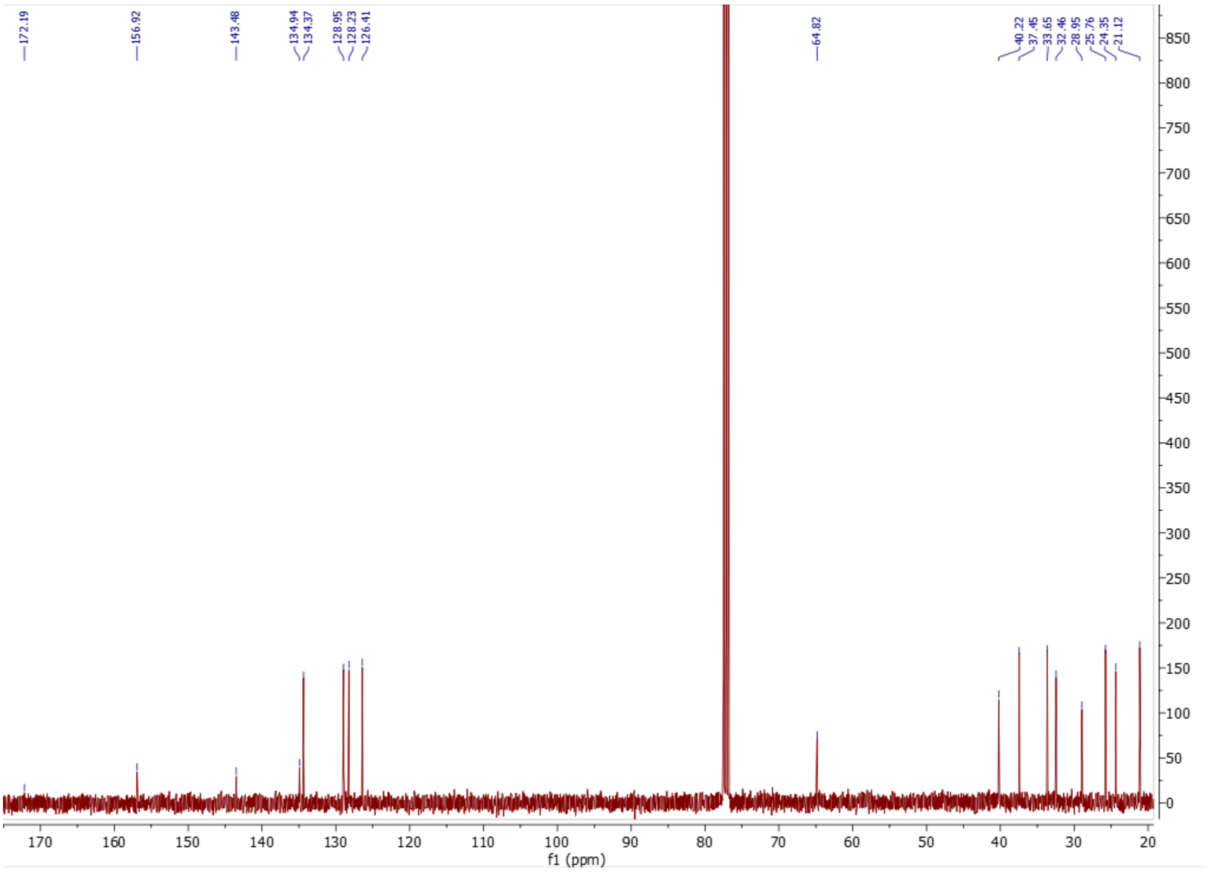
172.19
156.92
143.48
134.94
134.37
128.95
128.23
126.41
64.82
40.22
37.45
33.65
32.46
28.95
25.76
24.35
21.12
f1 (ppm)

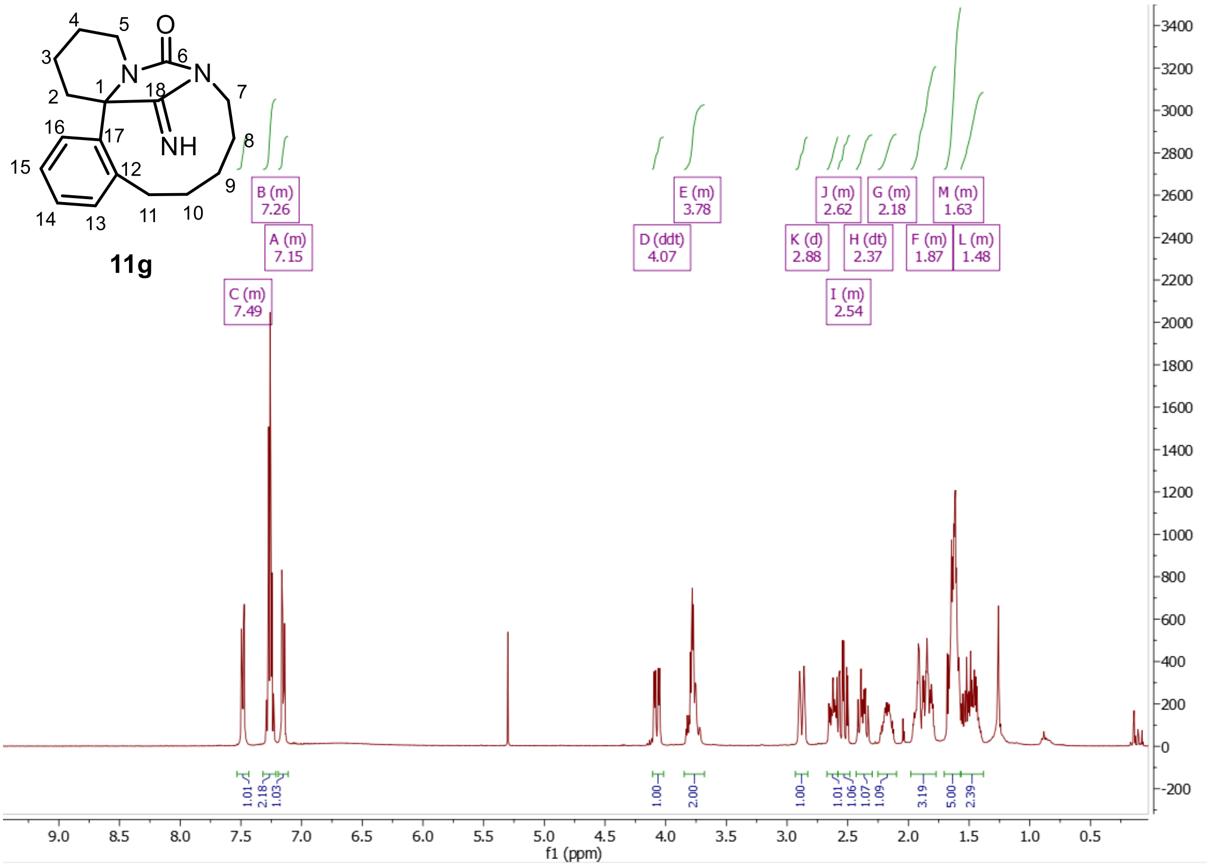
11g
C (m) 7.49
B (m) 7.26
A (m) 7.15
D (ddt) 4.07
E (m) 3.78
K (d) 2.88
J (m) 2.62
I (m) 2.54
H (dt) 2.37
G (m) 2.18
F (m) 1.87
M (m) 1.63
L (m) 1.48
f1 (ppm)

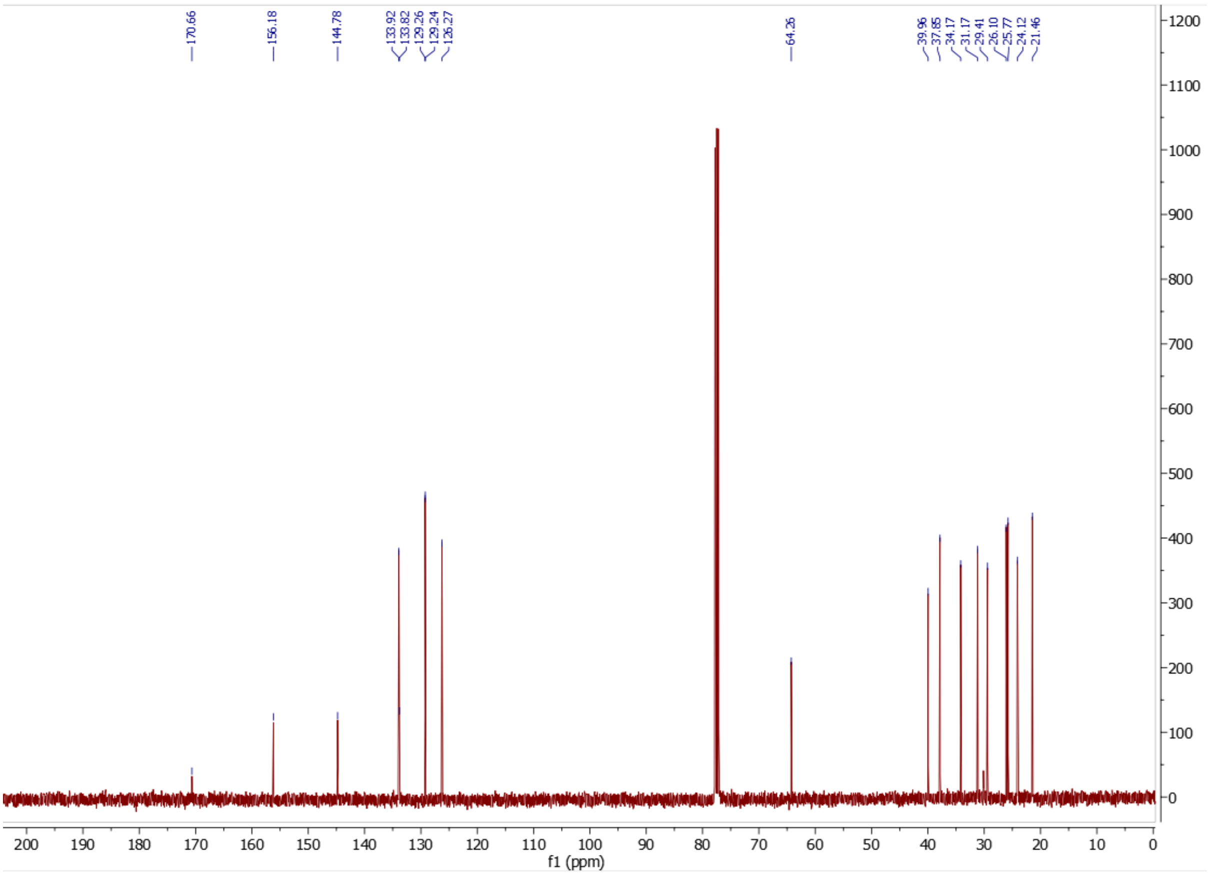
170.66
156.18
144.78
133.92
133.82
129.26
129.24
126.27
64.26
39.96
37.85
34.17
31.17
29.41
26.10
25.77
24.12
21.46
f1 (ppm)

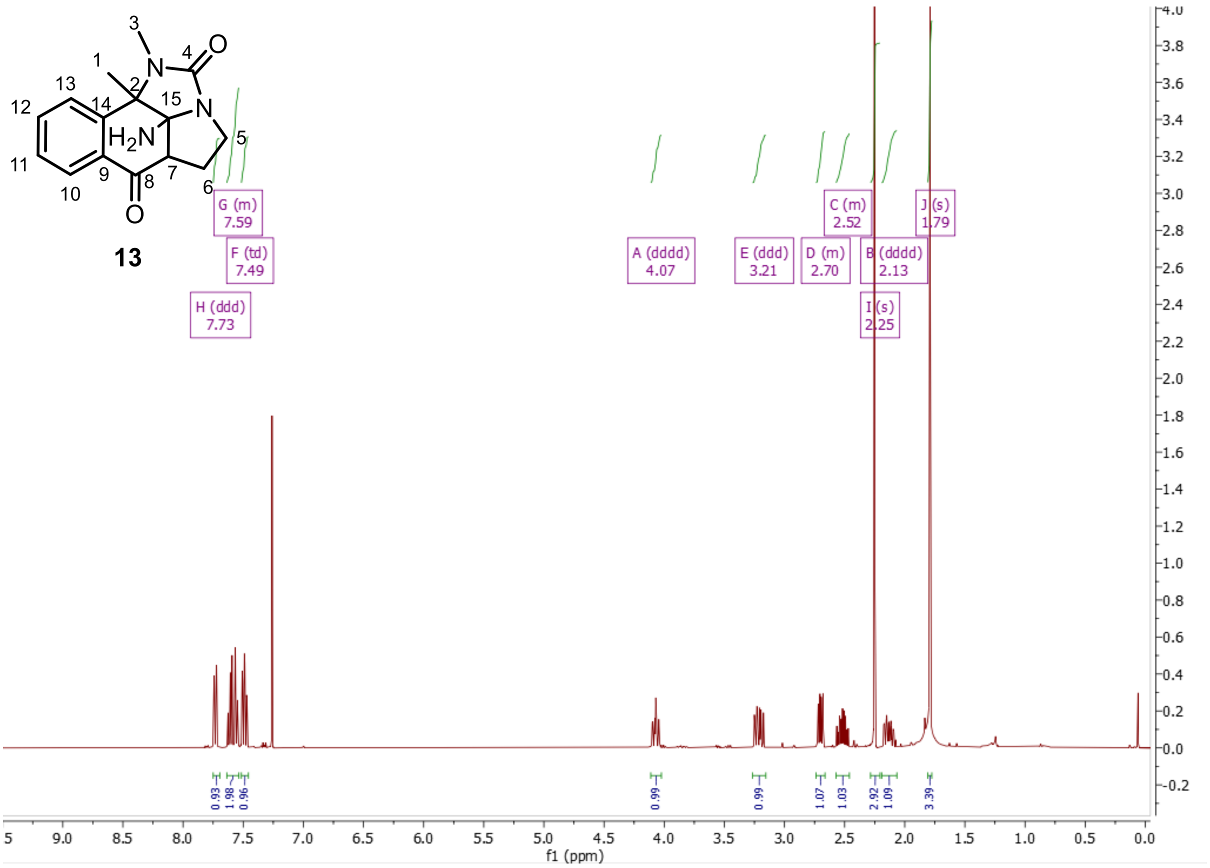
13
G (m) 7.59
F (td) 7.49
H (ddd) 7.73
A (dddd) 4.07
E (ddd) 3.21
D (m) 2.70
C (m) 2.52
B (dddd) 2.13
I (s) 2.25
J (s) 1.79
0.93
1.98
0.96
0.99
0.99
1.07
1.03
2.92
1.09
3.39
f1 (ppm)

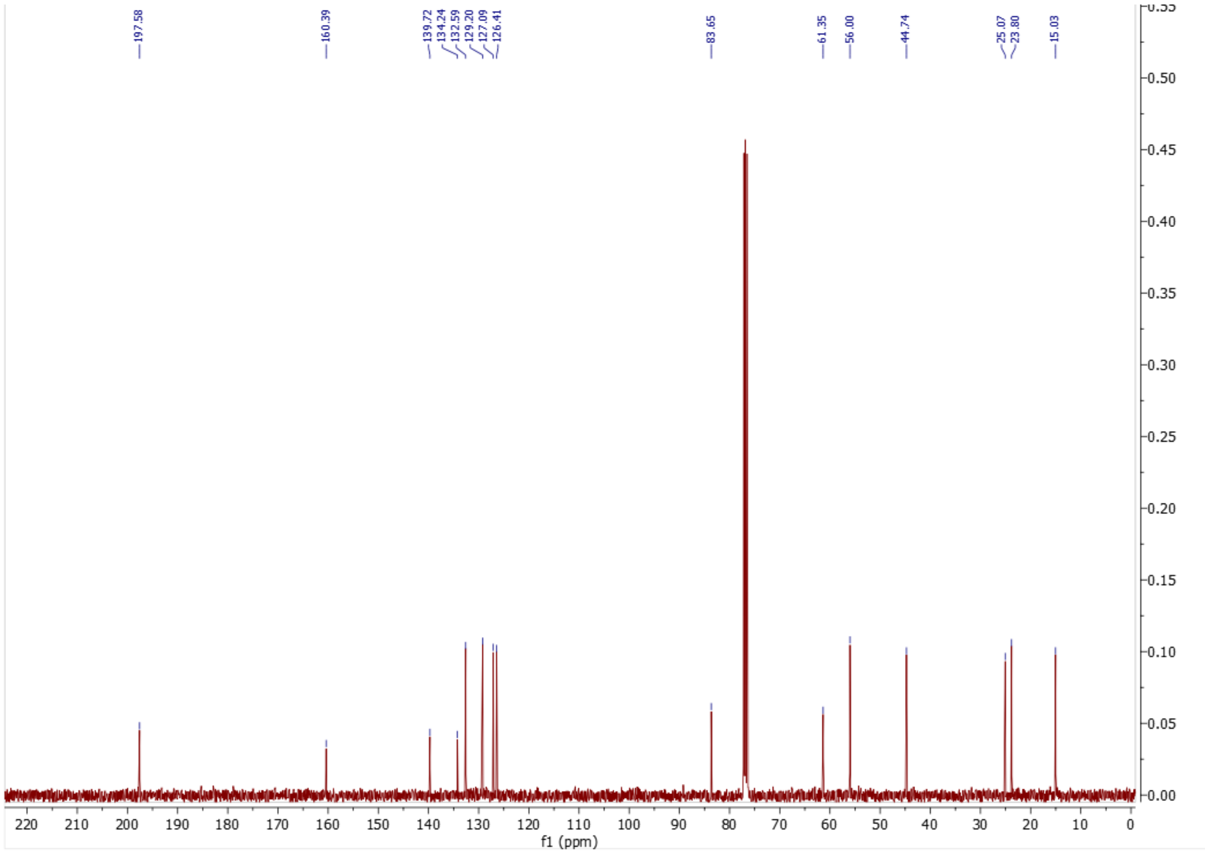
197.58
160.39
139.72
134.24
132.59
129.20
127.09
126.41
83.65
61.35
56.00
44.74
25.07
23.80
15.03
f1 (ppm)

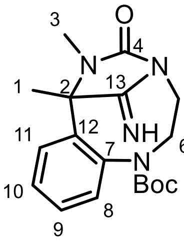
3
O
4
N
N
1
2
13
5
11
12
NH
7
6
N
10
Boc
8
9

15

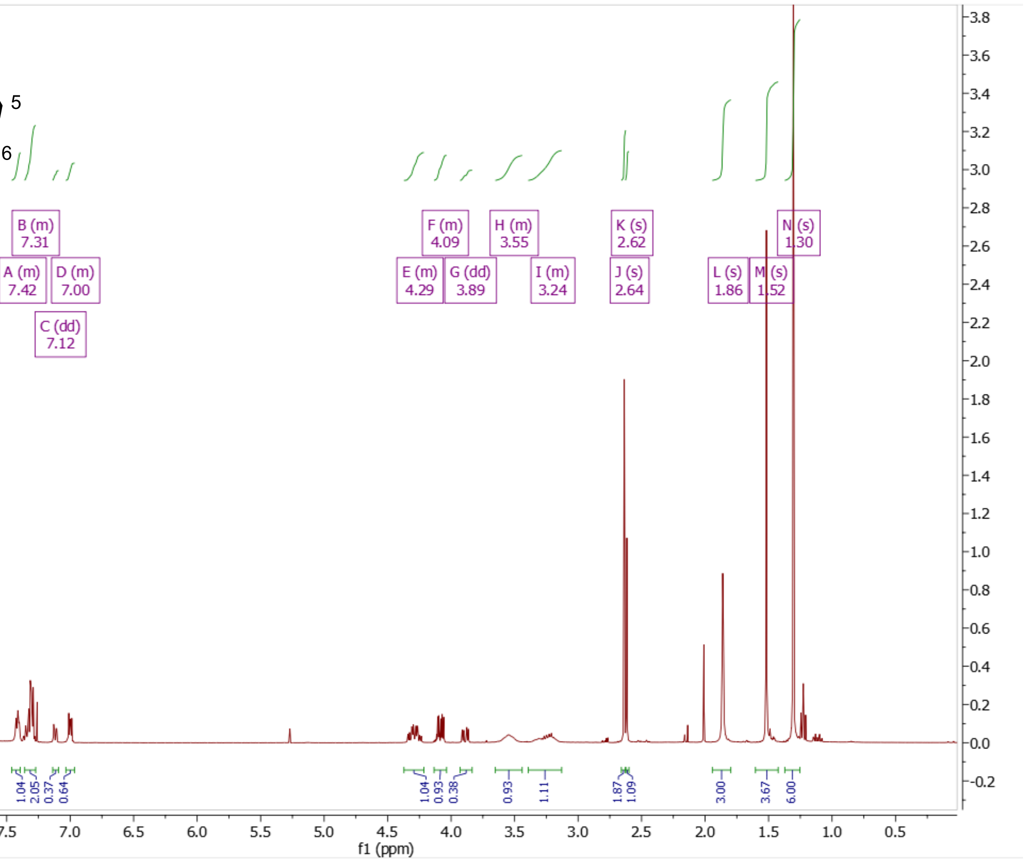
B (m) 7.31
A (m) 7.42
D (m) 7.00
C (dd) 7.12
F (m) 4.09
H (m) 3.55
E (m) 4.29
G (dd) 3.89
I (m) 3.24
K (s) 2.62
J (s) 2.64
L (s) 1.86
M (s) 1.52
N (s) 1.30
1.04
2.05
0.37
0.64
1.04
0.93
0.38
0.93
1.11
1.87
1.09
3.00
3.67
6.00
f1 (ppm)

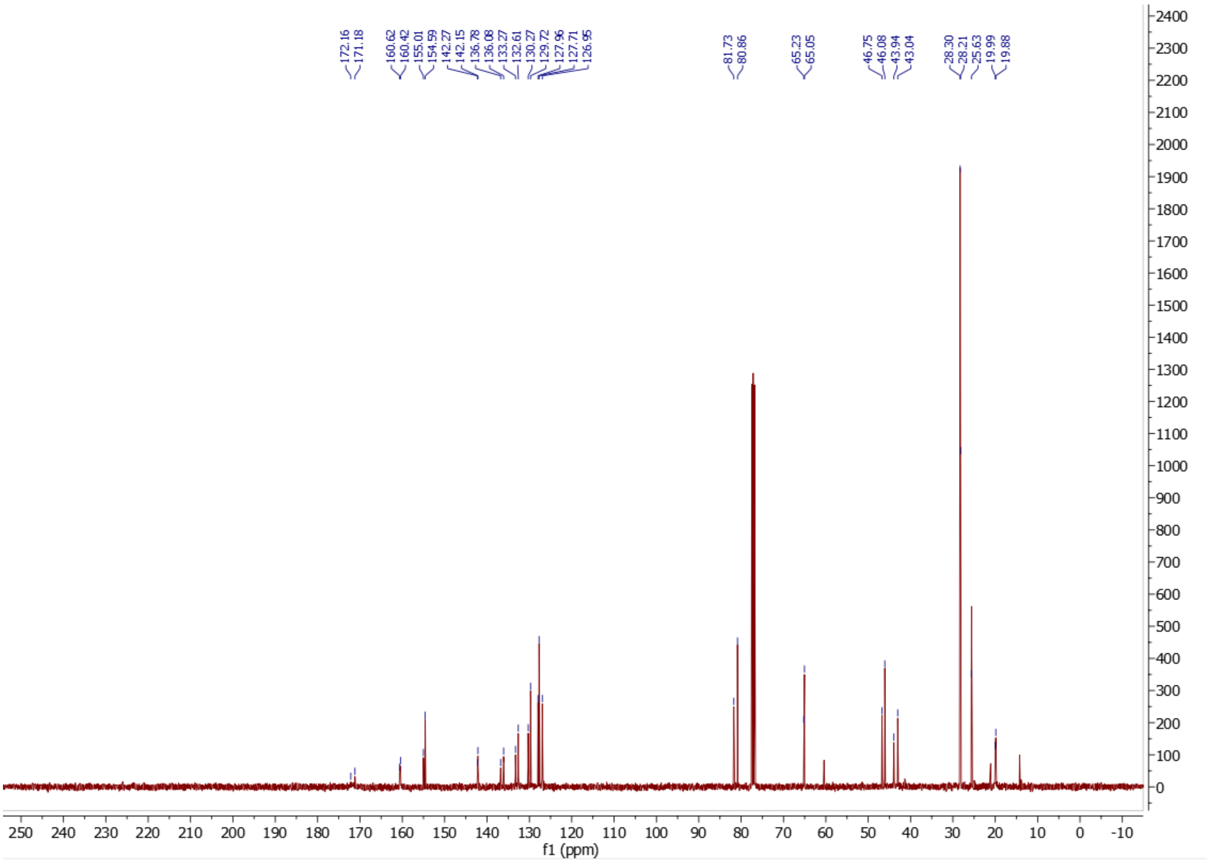
172.16
171.18
160.62
160.42
155.01
154.59
142.27
142.15
136.78
136.08
133.27
132.61
130.27
129.72
127.96
127.71
126.95
81.73
80.86
65.23
65.05
46.75
46.08
43.94
43.04
28.30
28.21
25.63
19.99
19.88
f1 (ppm)

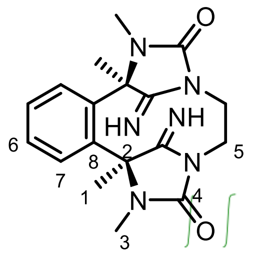

N
O
N
HN
NH
N
5
6
8
2
7
1
N
4
O
3

18a

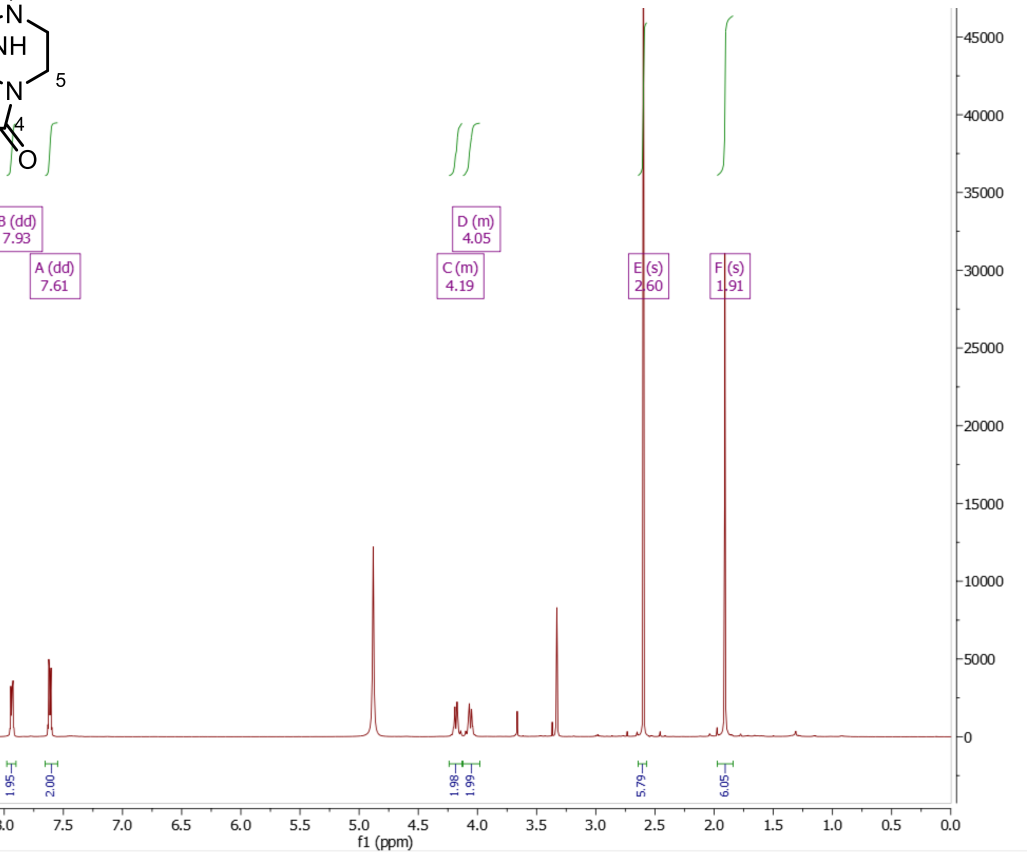

B (dd)
7.93
A (dd)
7.61
D (m)
4.05
C (m)
4.19
E (s)
2.60
F (s)
1.91
1.95
2.00
1.98
1.99
5.79
6.05
f1 (ppm)

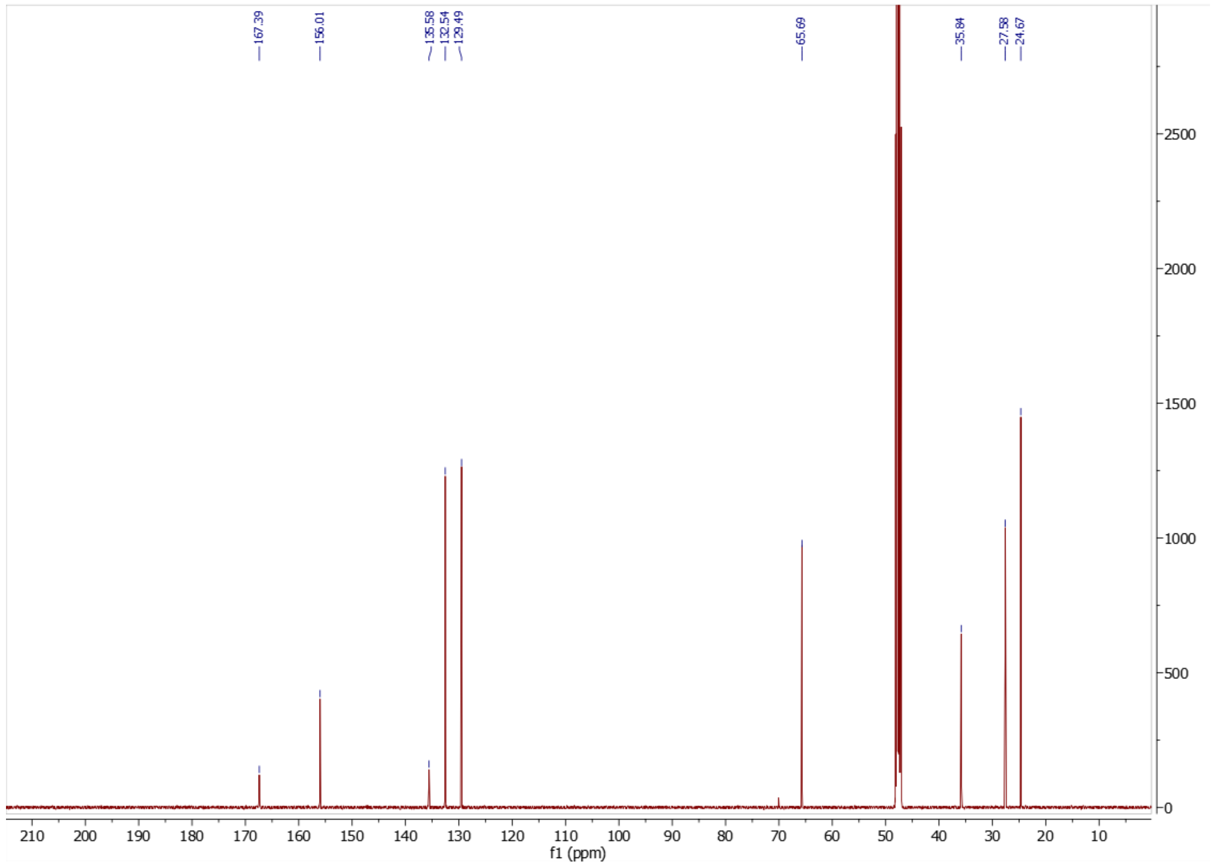

167.39
156.01
135.58
132.54
129.49
65.69
35.84
27.58
24.67
f1 (ppm)

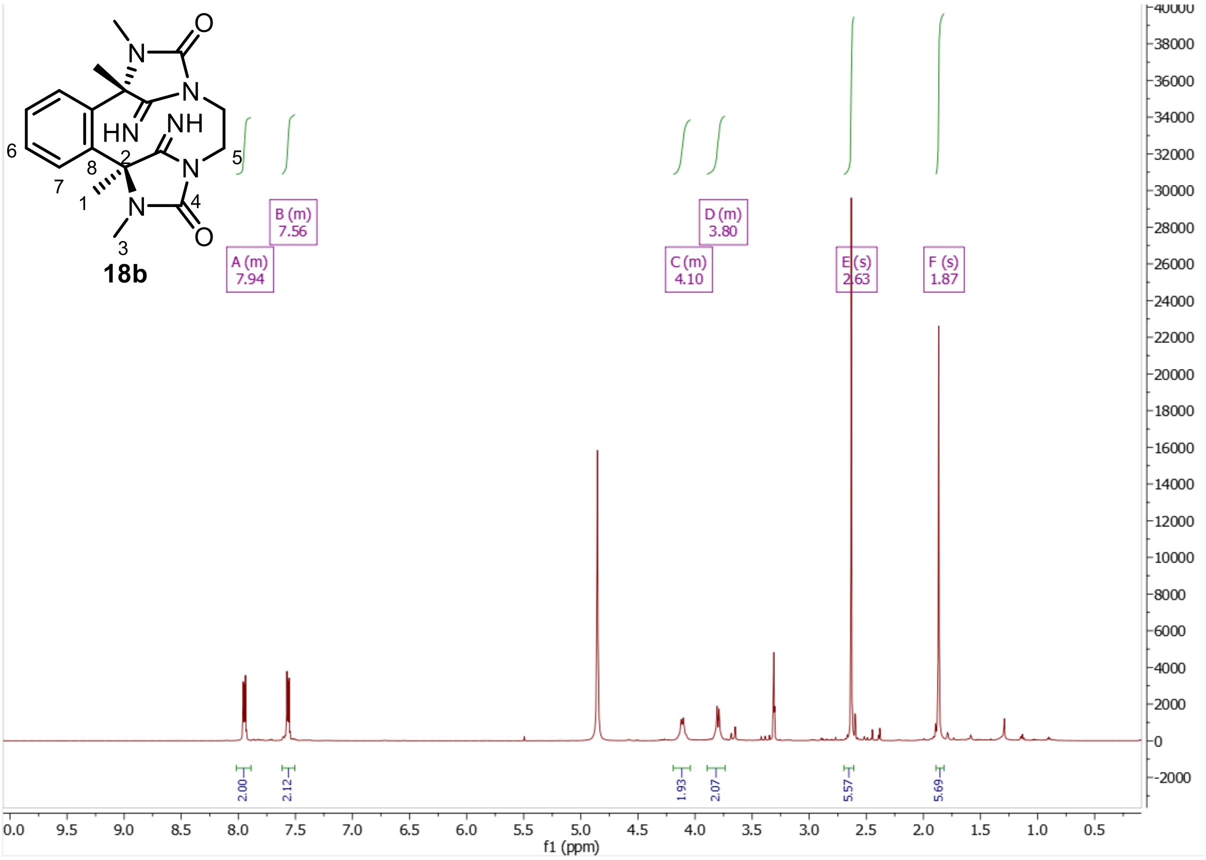
18b
A (m)
7.94
B (m)
7.56
C (m)
4.10
D (m)
3.80
E (s)
2.63
F (s)
1.87
2.00
2.12
1.93
2.07
5.57
5.69
f1 (ppm)

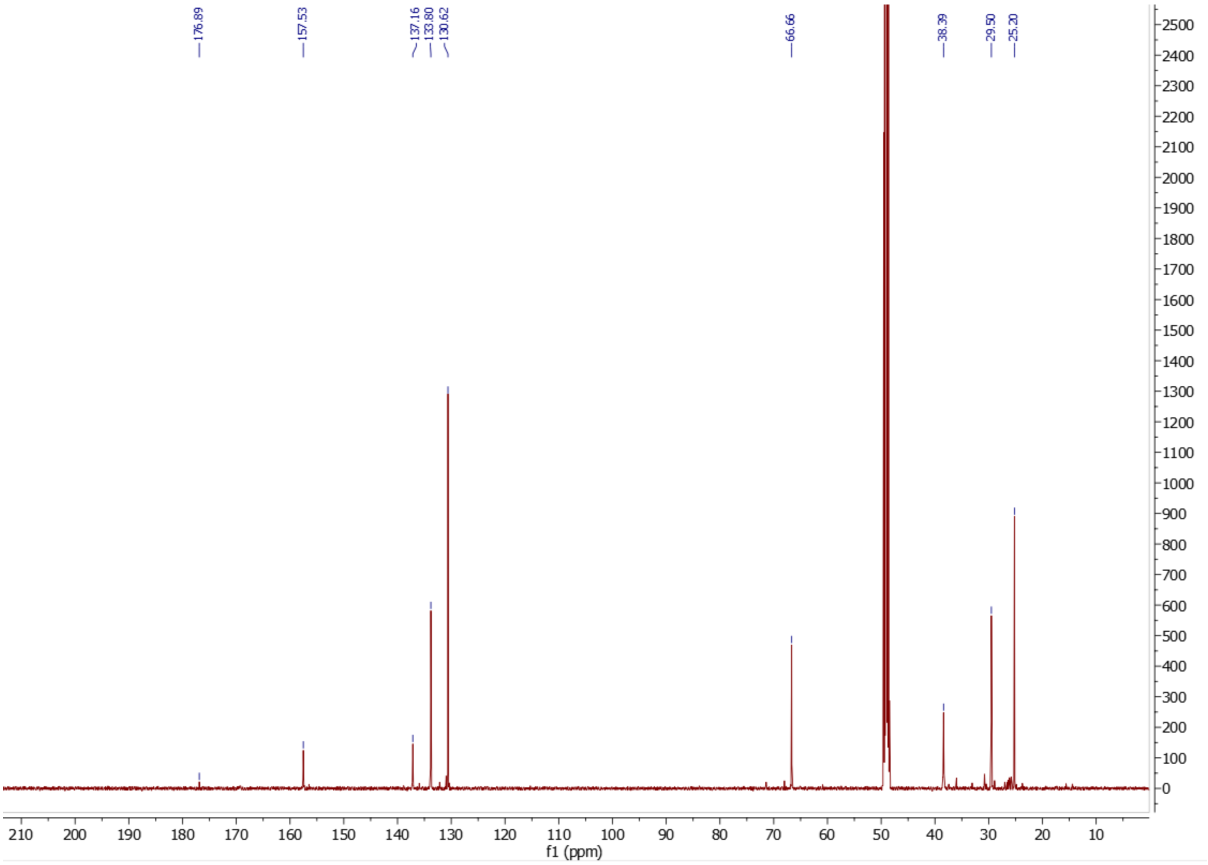
176.89
157.53
137.16
133.80
130.62
66.66
38.39
29.50
25.20
f1 (ppm)

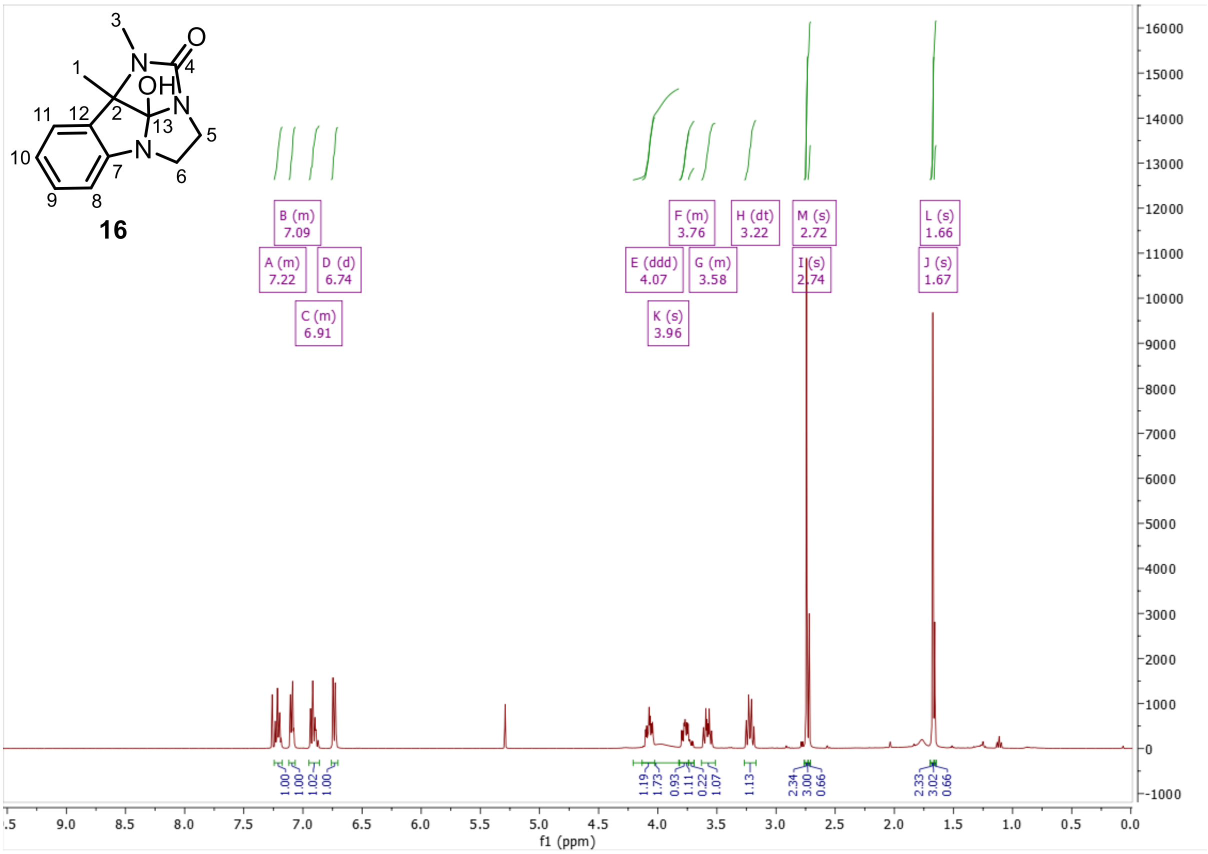
B (m)
7.09
A (m)
7.22
D (d)
6.74
C (m)
6.91
F (m)
3.76
H (dt)
3.22
M (s)
2.72
L (s)
1.66
E (ddd)
4.07
G (m)
3.58
I (s)
2.74
J (s)
1.67
K (s)
3.96
f1 (ppm)

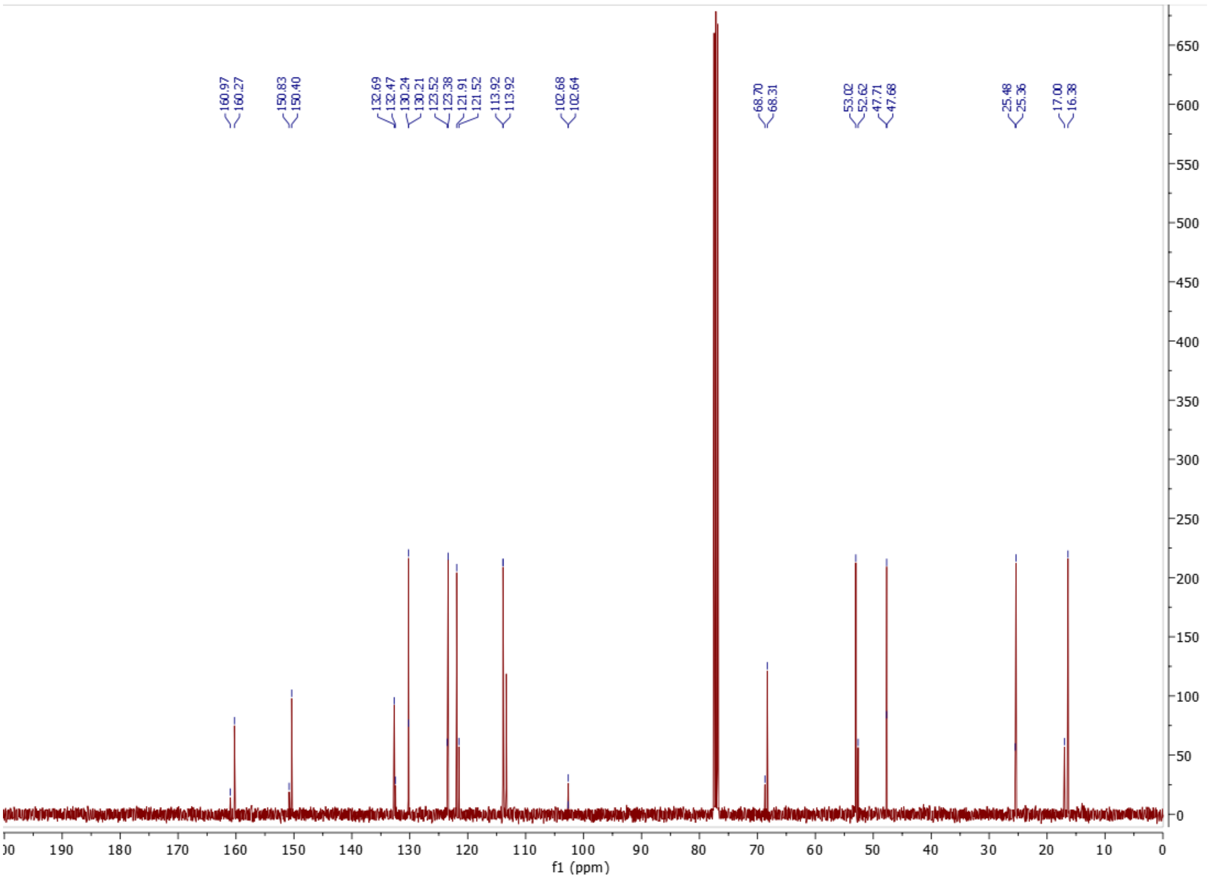
160.97
160.27
150.83
150.40
132.69
132.47
130.24
130.21
123.52
123.38
121.91
121.52
113.92
113.92
102.68
102.64
68.70
68.31
53.02
52.62
47.71
47.68
25.48
25.36
17.00
16.38
f1 (ppm)

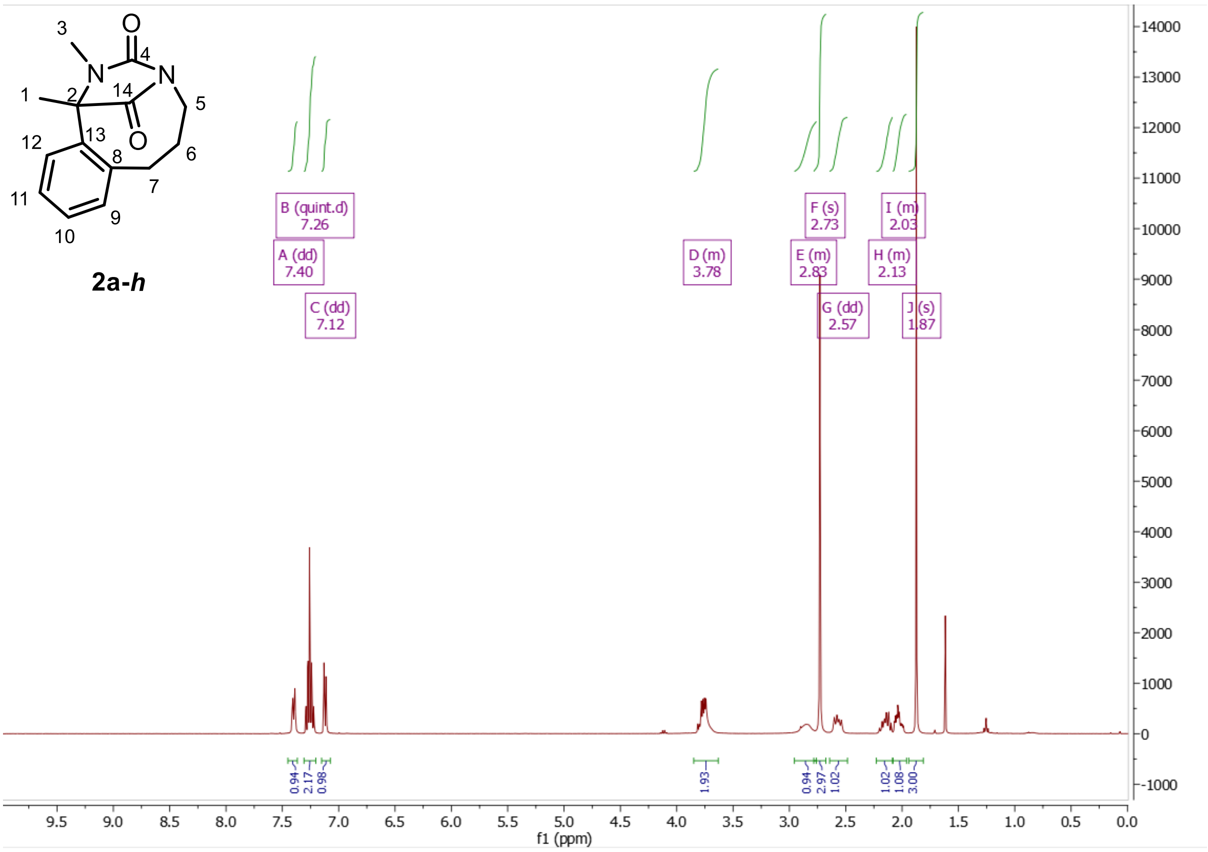

2a-h
B (quint.d) 7.26
A (dd) 7.40
C (dd) 7.12
D (m) 3.78
E (m) 2.83
F (s) 2.73
G (dd) 2.57
H (m) 2.13
I (m) 2.03
J (s) 1.87
0.94 2.17 0.98 1.93 0.94 2.97 1.02 1.02 1.08 3.00
f1 (ppm)

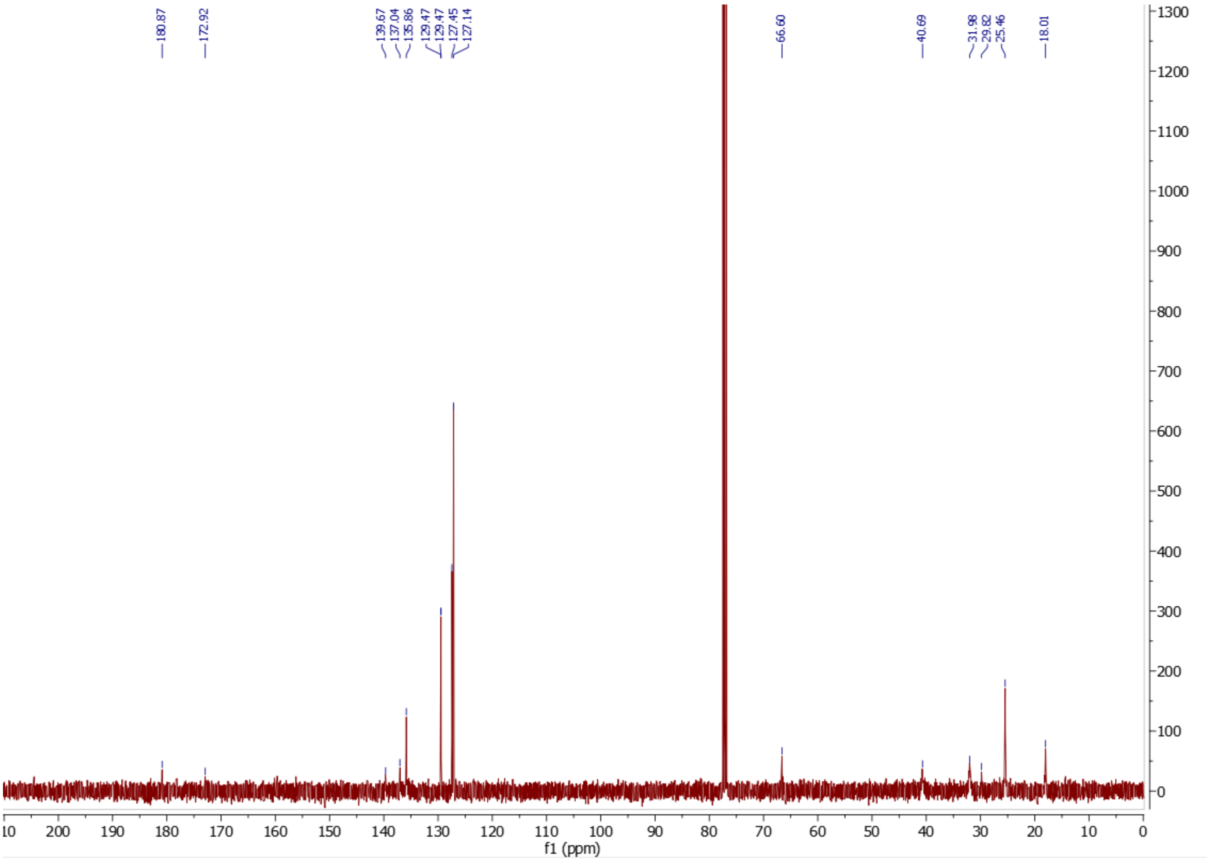

180.87
172.92
139.67 137.04 135.86 129.47 129.47 127.45 127.14
66.60
40.69
31.98 29.82 25.46
18.01
f1 (ppm)

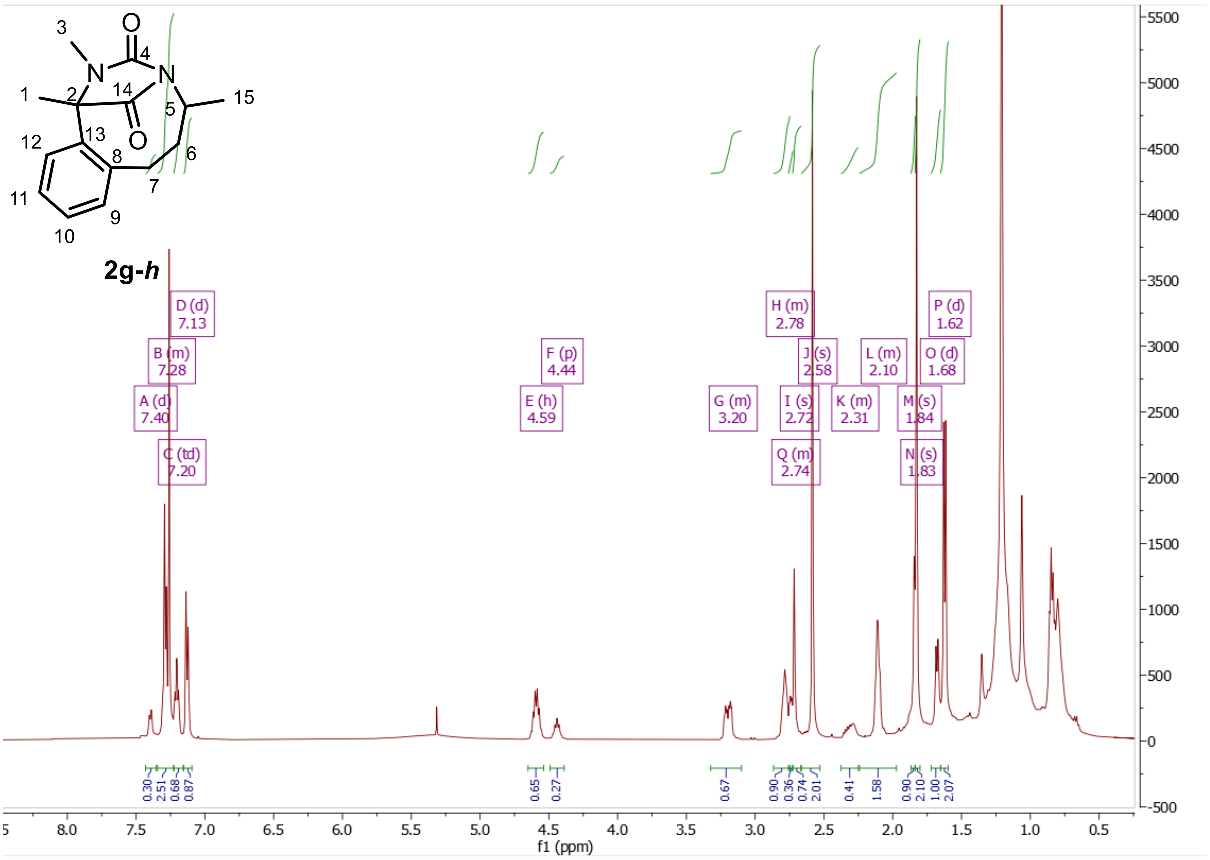
2g-*h*
A (d) 7.40
B (m) 7.28
C (td) 7.20
D (d) 7.13
E (h) 4.59
F (p) 4.44
G (m) 3.20
H (m) 2.78
Q (m) 2.74
I (s) 2.72
J (s) 2.58
K (m) 2.31
L (m) 2.10
M (s) 1.84
N (s) 1.83
O (d) 1.68
P (d) 1.62
f1 (ppm)

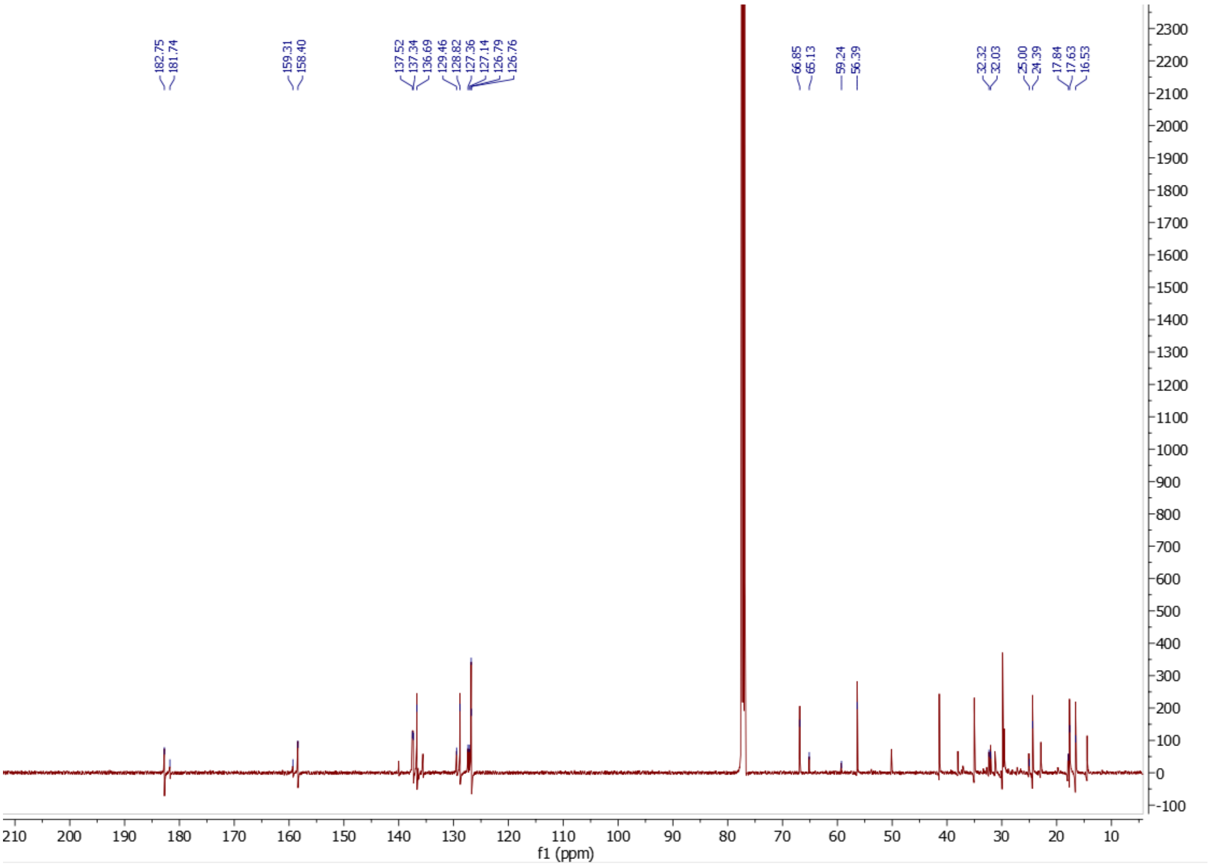
182.75
181.74
159.31
158.40
137.52
137.34
136.69
129.46
128.82
127.36
127.14
126.79
126.76
66.85
65.13
59.24
56.39
32.32
32.03
25.00
24.39
17.84
17.63
16.53
f1 (ppm)

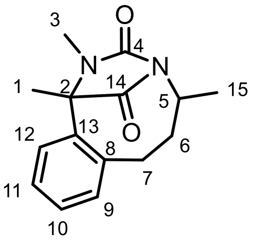
O
N
4
N
3
1
2
14
5
15
13
O
12
8
6
11
7
9
10

2g'-*h*

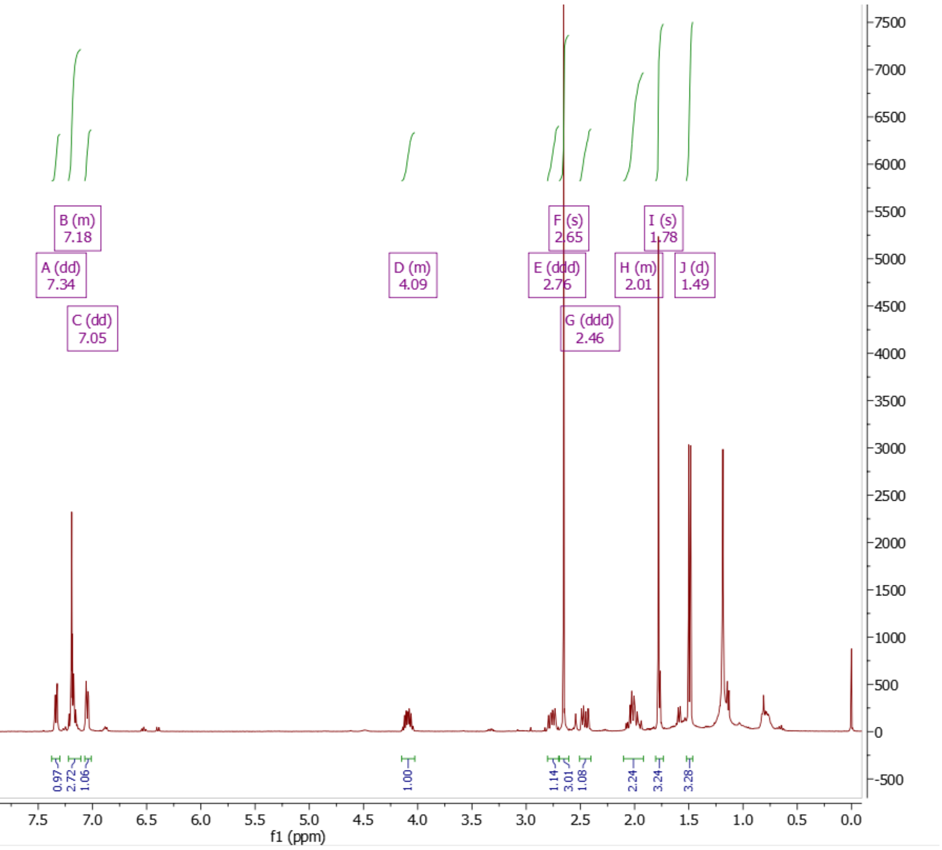
B (m) 7.18
A (dd) 7.34
C (dd) 7.05
D (m) 4.09
F (s) 2.65
E (ddd) 2.76
G (ddd) 2.46
I (s) 1.78
H (m) 2.01
J (d) 1.49
0.97
2.72
1.06
1.00
1.14
3.01
1.08
2.24
3.24
3.28
f1 (ppm)

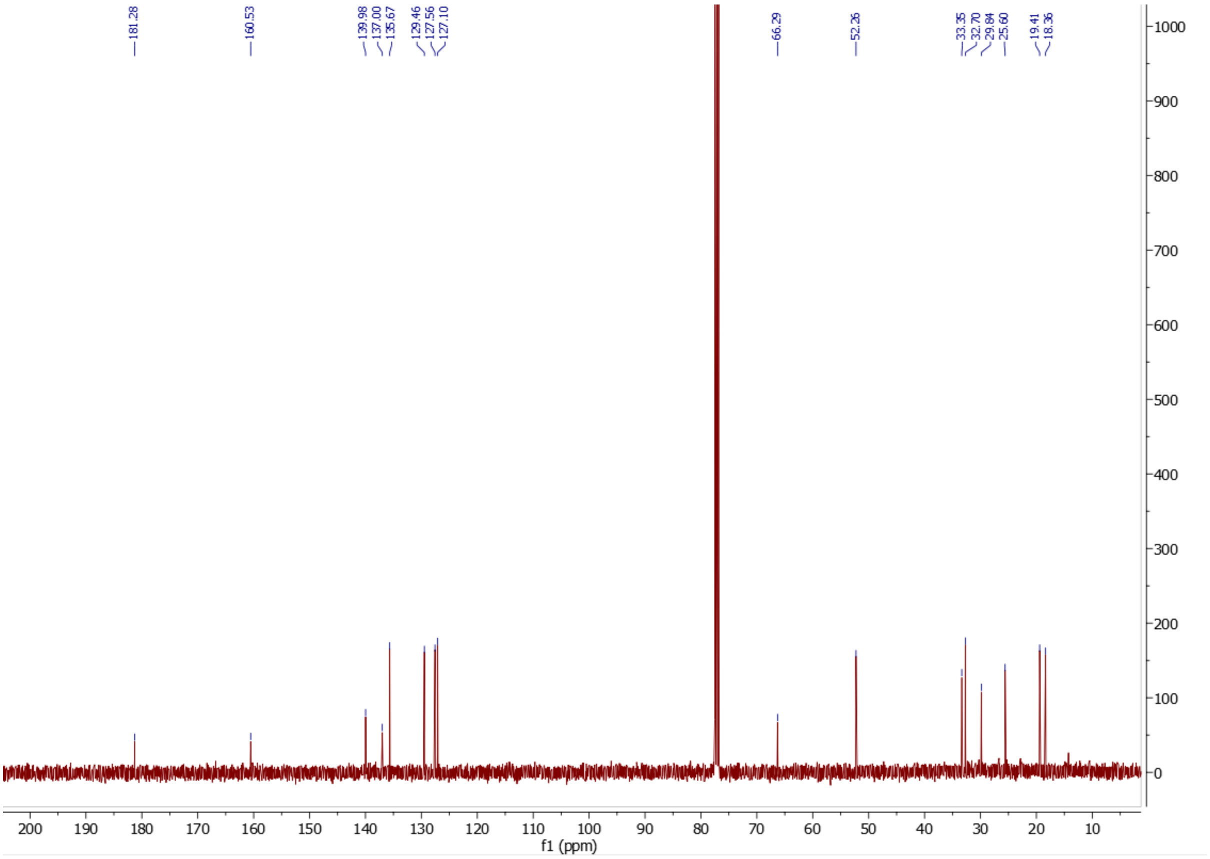
181.28
160.53
139.98
137.00
135.67
129.46
127.56
127.10
66.29
52.26
33.35
32.70
29.84
25.60
19.41
18.36
f1 (ppm)

4. X-Ray Crystal Data

X-ray diffraction experiments were carried out at 100(2) K on a Bruker APEX II diffractometer using Mo-K_α radiation (λ = 0.71073 Å). Intensities were integrated in SAINT[10] from several and absorption corrections based on equivalent reflections were applied using SADABS.[11] Structures were solved using She1XS[12] Superflip,[13,14] or She1XT[15] all of the structures were refined by full matrix least squares against F_2 in She1XL[12,16] using Olex2.[17] All of the non-hydrogen atoms were refined anisotropically. While all of the hydrogen atoms were located geometrically and refined using a riding model. Crystallographic data for compounds **2a**, **2f**, **2g**, **4**, **11a**, **11f**, **11g**, **16**, **18a** and **2g-h** have been deposited with the Cambridge Data Centre as supplementary publication CCDC 1993726-1993735. Copies of the data can be obtained free of charge on application to CCDC, 12 Union Road, Cambridge, CB2 1EZ, UK [fax(+44) 1223 336033, e-mail: deposit@ccdc.cam.ac.uk].

X-Ray Data for (2a):

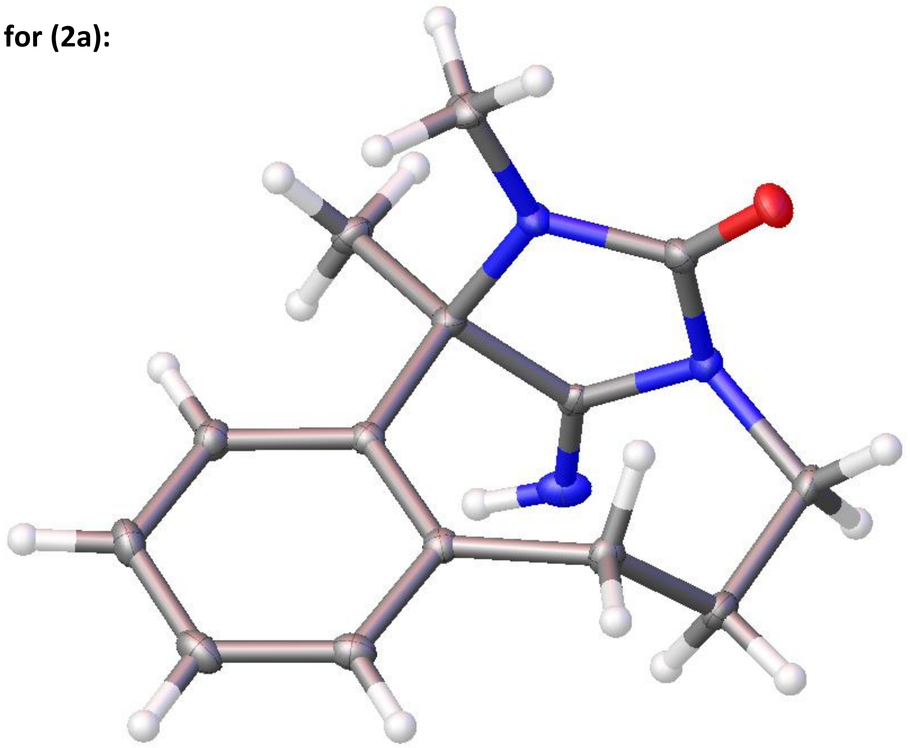

**Empirical formula**	$C_{14}H_{19}N_3O_2$
**Formula weight**	261.32
**Temperature/K**	100(2)
**Crystal system**	monoclinic
**Space group**	$P2_{1/c}$
**a/Å**	8.1323(2)
**b/Å**	8.1695(2)
**c/Å**	19.3253(4)
**α/°**	90
**β/°**	90.8030(10)
**γ/°**	90
**Volume/Å^3**	1283.79(5)
**Z**	4
**ρ_{calc}g/cm^3**	1.352
**μ/mm^{-1}**	0.092
**F(000)**	560.0
**Crystal size/mm^3**	0.492 x 0.406 x 0.373
**Radiation**	MoKα (λ = 0.71073)
**2Θ range for data collection**	5.01 to 55.804
**Index ranges**	-10 ≤ h ≤ 10, -10 ≤ k ≤ 7, -21 ≤ l ≤ 25
**Reflections collected**	11411
**Independent reflections**	3068 [R_{int} = 0.0225, R_{sigma} = 0.0217]
**Data/restraints/parameters**	3068/1/181
**Goodness-of-fit on F^2**	1.119
**Final R indexes [I>=2σ (I)]**	R_1 = 0.0448, wR_2 = 0.1036
**Final R indexes [all data]**	R_1 = 0.0507, wR_2 = 0.1067
**Largest diff. peak/hole/ eÅ^{-3}**	0.45/-0.35

X-Ray Data for (4):

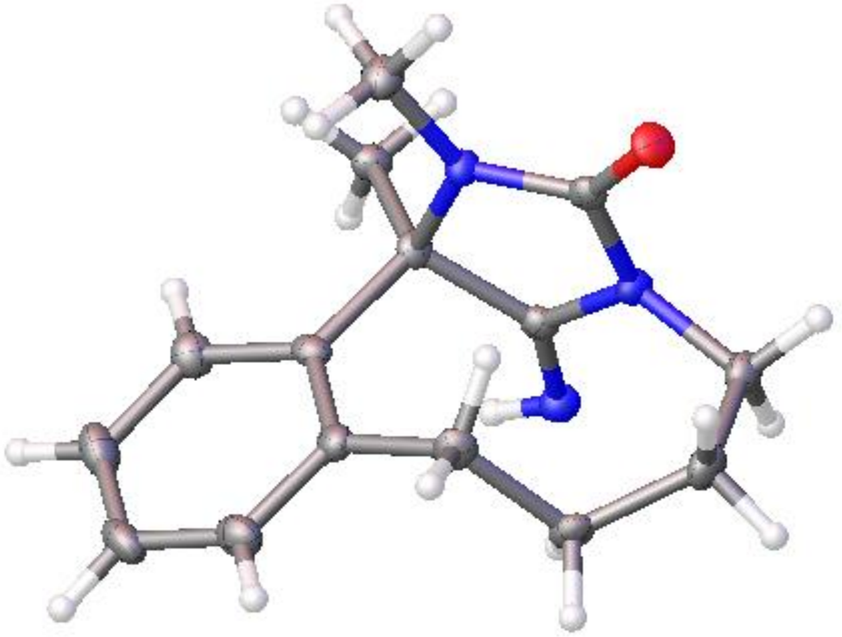

**Empirical formula**	$C_{15}H_{19}N_3O$
**Formula weight**	257.33
**Temperature/K**	10092)
**Crystal system**	trigonal
**Space group**	$P3_1$
**a/Å**	22.0392(6)
**b/Å**	22.0392(6)
**c/Å**	7.1184(2)
**α/°**	90
**β/°**	90
**γ/°**	120
**Volume/Å^3**	2994.36(18)
**Z**	9
**ρ_{calc}g/cm^3**	1.284
**μ/mm^{-1}**	0.0831242.0
**F(000)**	1242.0
**Crystal size/mm^3**	0.389 x 0.323 x 0.234
**Radiation**	MoKα (λ = 0.71073)
**2Θ range for data collection**	3.696 to 51.362
**Index ranges**	-26 ≤ h ≤ 26, -24 ≤ k ≤ 26, -8 ≤ l ≤ 8
**Reflections collected**	22901
**Independent reflections**	7518 [R_{int} = 0.0614, R_{sigma} = 0.0705]
**Data/restraints/parameters**	7518/1/532
**Goodness-of-fit on F^2**	1.023
**Final R indexes [I>=2σ (I)]**	R_1 = 0.0444, wR_2 = 0.0831
**Final R indexes [all data]**	R_1 = 0.0623, wR_2 = 0.0905
**Largest diff. peak/hole/ eÅ^{-3}**	0.16/-0.21

X-Ray Data for (2f):

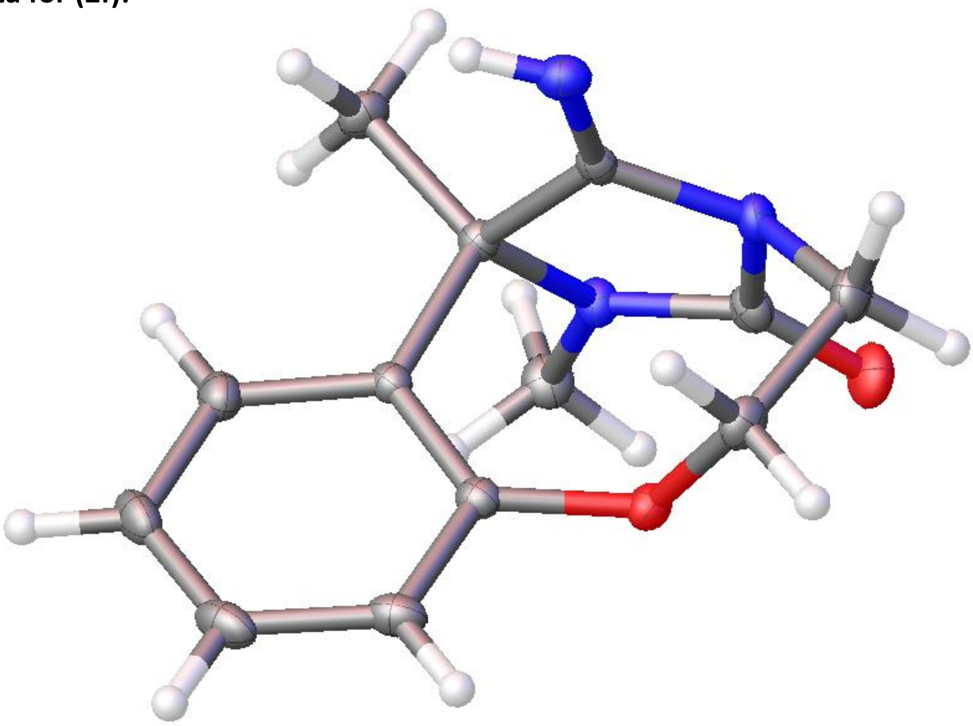

**Empirical formula**	$C_{13}H_{17}N_3O_3$
**Formula weight**	263.29
**Temperature/K**	99.97
**Crystal system**	monoclinic
**Space group**	$P2_{1/c}$
**a/Å**	8.0314(2)
**b/Å**	8.1488(2)
**c/Å**	19.2027(5)
**α/°**	90
**β/°**	90.5479917)
**γ/°**	90
**Volume/Å^3**	1256.69(5)
**Z**	4
**ρ_{calc}g/cm^3**	1.392
**μ/mm^{-1}**	0.101
**F(000)**	560.0
**Crystal size/mm^3**	0.394 x 0.347 x 0.144
**Radiation**	MoKα (λ = 0.71073)
**2Θ range for data collection**	4.242 to 59.146
**Index ranges**	-11 ≤ h ≤ 11, -11 ≤ k ≤ 11, -26 ≤ l ≤ 26
**Reflections collected**	26526
**Independent reflections**	3522 [R_{int} = 0.0329, R_{sigma} = 0.0184]
**Data/restraints/parameters**	3522/0/186
**Goodness-of-fit on F^2**	1.049
**Final R indexes [I>=2σ (I)]**	R_1 = 0.0378, wR_2 = 0.0952
**Final R indexes [all data]**	R_1 = 0.0449, wR_2 = 0.0998
**Largest diff. peak/hole/ eÅ^{-3}**	0.40/-0.22

X-Ray Data for (2g):

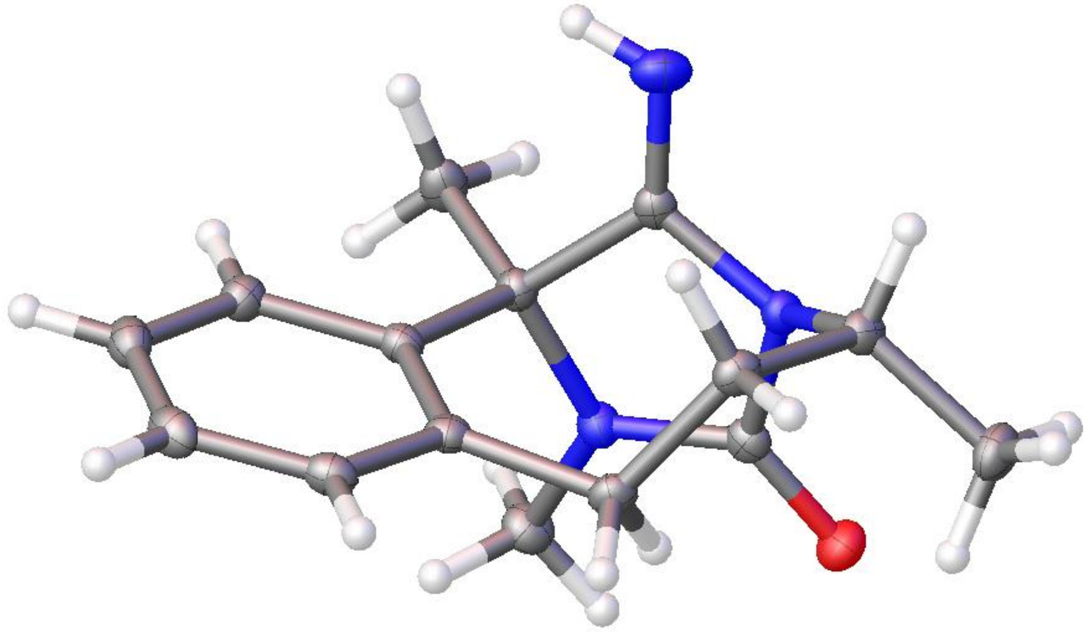

**Empirical formula**	$C_{15}H_{21}N_3O_2$
**Formula weight**	275.35
**Temperature/K**	100(2)
**Crystal system**	triclinic
**Space group**	P-1
**a/Å**	7.1405(3)
**b/Å**	8.5770(4)
**c/Å**	11.9704(6)
**α/°**	73.957(3)
**β/°**	88.399(3)
**γ/°**	78.976(3)
**Volume/Å^3**	691.32(6)
**Z**	2
**ρ_{calc}g/cm^3**	1.323
**μ/mm^{-1}**	0.090
**F(000)**	296.0
**Crystal size/mm^3**	0.382 x 0.326 x 0.149
**Radiation**	MoKα (λ = 0.71073)
**2Θ range for data collection**	3.542 to 55.856
**Index ranges**	-8 ≤ h ≤ 9, -11 ≤ k ≤ 11, -15 ≤ l ≤ 15
**Reflections collected**	12697
**Independent reflections**	3308 [R_{int} = 0.0270, R_{sigma} = 0.0248]
**Data/restraints/parameters**	3308/1/196
**Goodness-of-fit on F^2**	1.045
**Final R indexes [I>=2σ (I)]**	R_1 = 0.0395, wR_2 = 0.0994
**Final R indexes [all data]**	R_1 = 0.0471, wR_2 = 0.1040
**Largest diff. peak/hole/ eÅ^{-3}**	0.41/-0.23

X-Ray Data for (11a):

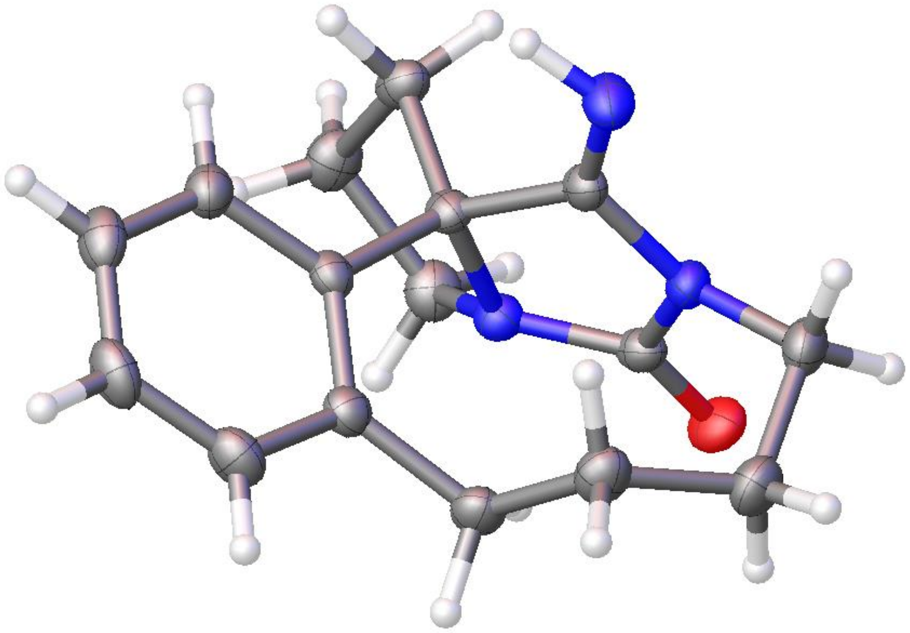

**Empirical formula**	$C_{16}H_{19}N_3O$
**Formula weight**	269.34
**Temperature/K**	100(2)
**Crystal system**	Triclinic
**Space group**	P-1
**a/Å**	6.9675(12)
**b/Å**	8.6877(14)
**c/Å**	12.0676(19)
**α/°**	87.020(11)
**β/°**	74.121(11)
**γ/°**	77.399(12)
**Volume/Å^3**	685.7(2)
**Z**	2
**ρ_{calc}g/cm^3**	1.305
**μ/mm^{-1}**	0.084
**F(000)**	288.0
**Crystal size/mm^3**	0.352 x 0.352 x 0.185
**Radiation**	MoKα (λ = 0.71073)
**2Θ range for data collection**	4.804 to 56.374
**Index ranges**	-9 ≤ h ≤ 9, -11 ≤ k ≤ 11, -15 ≤ l ≤ 15
**Reflections collected**	12669
**Independent reflections**	3290 [R_{int} = 0.0625, R_{sigma} = 0.0578]
**Data/restraints/parameters**	3290/0/185
**Goodness-of-fit on F^2**	1.034
**Final R indexes [I>=2σ (I)]**	R_1 = 0.0466, wR_2 = 0.1020
**Final R indexes [all data]**	R_1 = 0.0735, wR_2 = 0.1140
**Largest diff. peak/hole/ eÅ^{-3}**	0.23/-0.24

X-Ray Data for (11f):

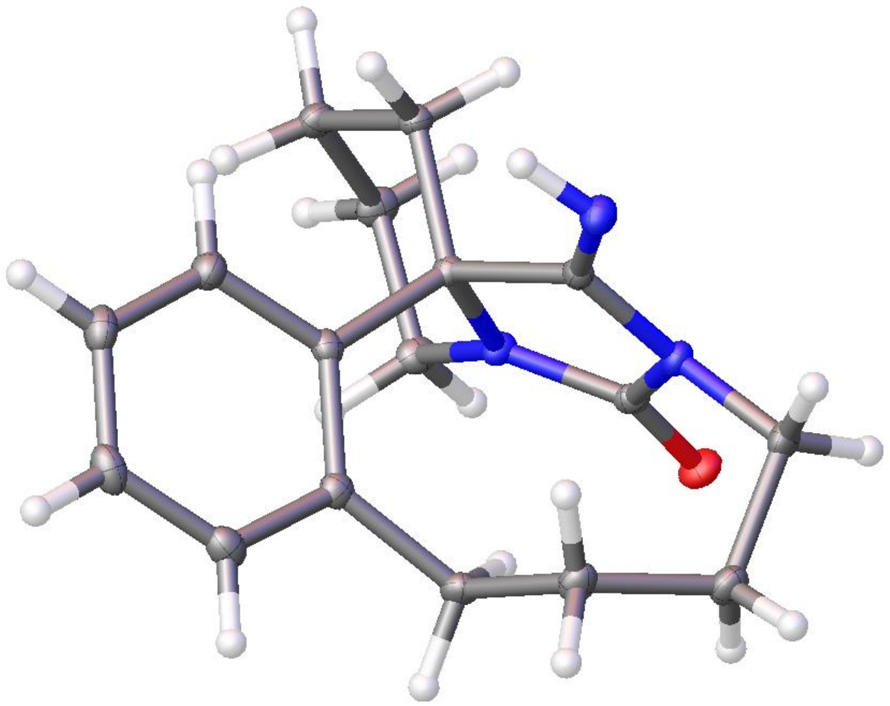

**Empirical formula**	$C_{17}H_{21}N_3O$
**Formula weight**	283.37
**Temperature/K**	100(2)
**Crystal system**	Triclinic
**Space group**	P-1
**a/Å**	7.0488(2)
**b/Å**	8.6329(3)
**c/Å**	12.6463(5)
**α/°**	88.989(2)
**β/°**	74.751(2)
**γ/°**	77.777(2)
**Volume/Å^3**	725.03(4)
**Z**	2
**ρ_{calc}g/cm^3**	1.298
**μ/mm^{-1}**	0.083
**F(000)**	304.0
**Crystal size/mm^3**	0.428 x 0.329 x 0.322
**Radiation**	MoKα (λ = 0.71073)
**2Θ range for data collection**	3.34 to 56.23
**Index ranges**	-9 ≤ h ≤ 9, -11 ≤ k ≤ 11, -16 ≤ l ≤ 16
**Reflections collected**	13480
**Independent reflections**	3531 [R_{int} = 0.0223, R_{sigma} = 0.0203]
**Data/restraints/parameters**	3531/0/194
**Goodness-of-fit on F^2**	1.034
**Final R indexes [I>=2σ (I)]**	R_1 = 0.0378, wR_2 = 0.0899
**Final R indexes [all data]**	R_1 = 0.0433, wR_2 = 0.0940
**Largest diff. peak/hole/ eÅ^{-3}**	0.33/-0.22

X-Ray Data for (11g):

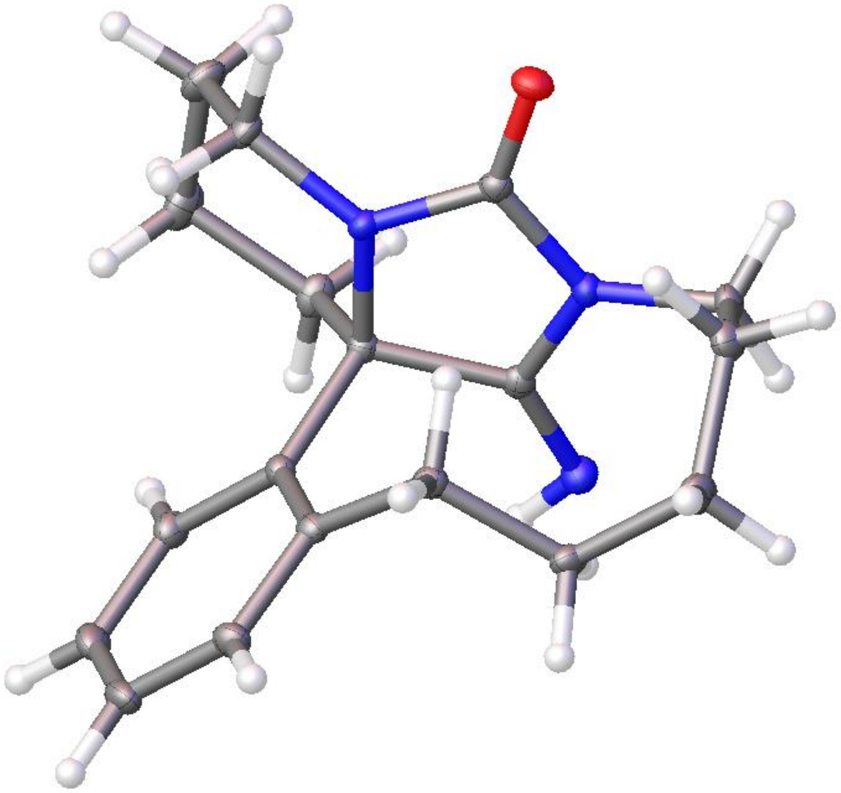

**Empirical formula**	$C_{18}H_{26}N_3O_{2.5}$
**Formula weight**	324.42
**Temperature/K**	100.01
**Crystal system**	triclinic
**Space group**	P-1
**a/Å**	7.6118(2)
**b/Å**	9.3656(3)
**c/Å**	12.1634(4)
**α/°**	93.7306(19)
**β/°**	95.390(2)
**γ/°**	108.1269(18)
**Volume/Å^3**	816.35(4)
**Z**	2
**ρ_{calc}g/cm^3**	1.320
**μ/mm^{-1}**	0.089
**F(000)**	350.0
**Crystal size/mm^3**	0.534 x 0.264 x 0.196
**Radiation**	MoKα (λ = 0.71073)
**2Θ range for data collection**	3.38 to 61.014
**Index ranges**	-10 ≤ h ≤ 10, -13 ≤ k ≤ 13, -17 ≤ l ≤ 17
**Reflections collected**	18692
**Independent reflections**	4993 [R_{int} = 0.0296, R_{sigma} = 0.0292]
**Data/restraints/parameters**	4993/0/232
**Goodness-of-fit on F^2**	1.039
**Final R indexes [I>=2σ (I)]**	R_1 = 0.0428, wR_2 = 0.1072
**Final R indexes [all data]**	R_1 = 0.0557, wR_2 = 0.1150
**Largest diff. peak/hole/ eÅ^{-3}**	0.41/-0.36

X-Ray Data for (16):

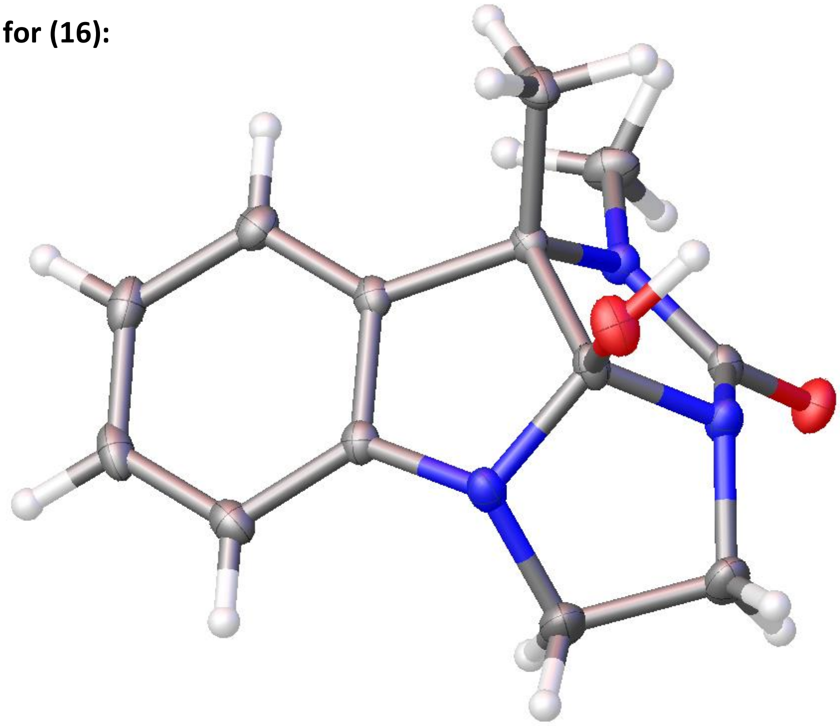

**Empirical formula**	$C_{13}H_{15}N_3O_2$
**Formula weight**	245.28
**Temperature/K**	100(2)
**Crystal system**	orthorhombic
**Space group**	Pbca
**a/Å**	13.4226(6)
**b/Å**	7.6316(4)
**c/Å**	22.4832(11)
**α/°**	90
**β/°**	90
**γ/°**	90
**Volume/Å^3**	2303.09(19)
**Z**	8
**ρ_{calc}g/cm^3**	1.415
**μ/mm^{-1}**	0.098
**F(000)**	1040.0
**Crystal size/mm^3**	0.461 x 0.284 x 0.24
**Radiation**	MoKα (λ = 0.71073)
**2Θ range for data collection**	3.624 to 55.97
**Index ranges**	-17 ≤ h ≤ 17, -10 ≤ k ≤ 10, -29 ≤ l ≤ 28
**Reflections collected**	19730
**Independent reflections**	2763 [R_{int} = 0.0432, R_{sigma} = 0.0251]
**Data/restraints/parameters**	2763/0/169
**Goodness-of-fit on F^2**	1.047
**Final R indexes [I>=2σ (I)]**	R_1 = 0.0413, wR_2 = 0.0977
**Final R indexes [all data]**	R_1 = 0.0543, wR_2 = 0.1046
**Largest diff. peak/hole/ eÅ^{-3}**	0.33/-0.25

X-Ray Data for (18a):

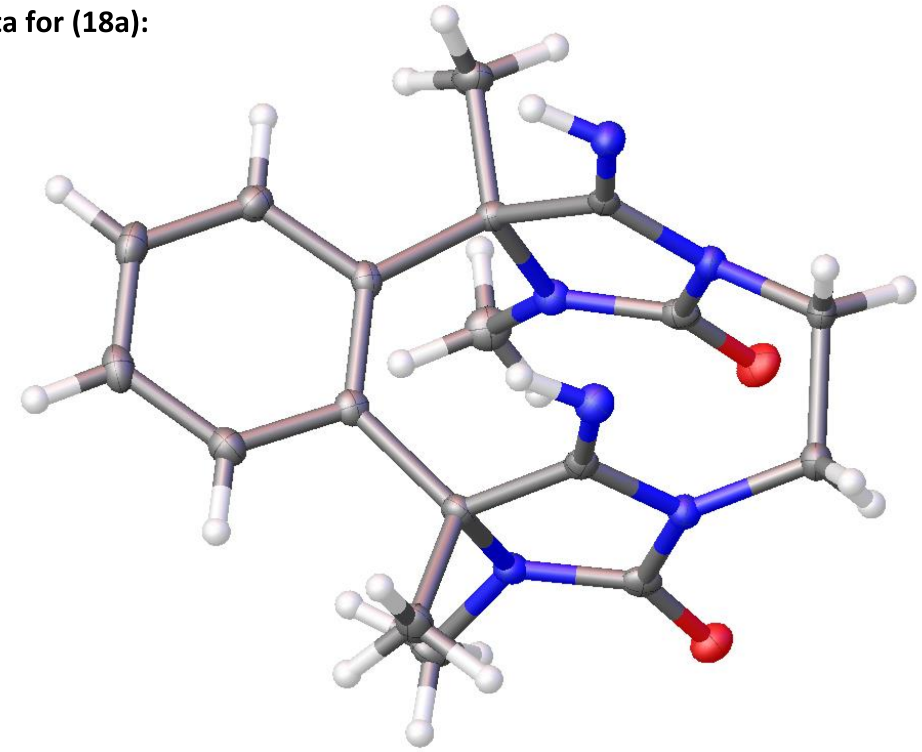

**Empirical formula**	$C_{18}H_{26}N_6O_4$
**Formula weight**	390.45
**Temperature/K**	100(2)
**Crystal system**	Orthorhombic
**Space group**	$P2_12_12_1$
**a/Å**	8.3617(3)
**b/Å**	13.8207(6)
**c/Å**	16.0698(6)
**α/°**	90
**β/°**	90
**γ/°**	90
**Volume/Å^3**	1857.10(13)
**Z**	4
**ρ_{calc}g/cm^3**	1.396
**μ/mm^{-1}**	0.101
**F(000)**	832.0
**Crystal size/mm^3**	0.443 x 0.367 x 0.154
**Radiation**	MoKα (λ = 0.71073)
**2Θ range for data collection**	3.886 to 55.938
**Index ranges**	-11 ≤ h ≤ 11, -18 ≤ k ≤ 18, -20 ≤ l ≤ 21
**Reflections collected**	17119
**Independent reflections**	4432 [R_{int} = 0.0451, R_{sigma} = 0.0416]
**Data/restraints/parameters**	4432/6/281
**Goodness-of-fit on F^2**	1.018
**Final R indexes [I>=2σ (I)]**	R_1 = 0.0355, wR_2 = 0.0754
**Final R indexes [all data]**	R_1 = 0.0444, wR_2 = 0.0800
**Largest diff. peak/hole/ eÅ^{-3}**	0.25/-0.18

X-Ray Data for (2g-h):

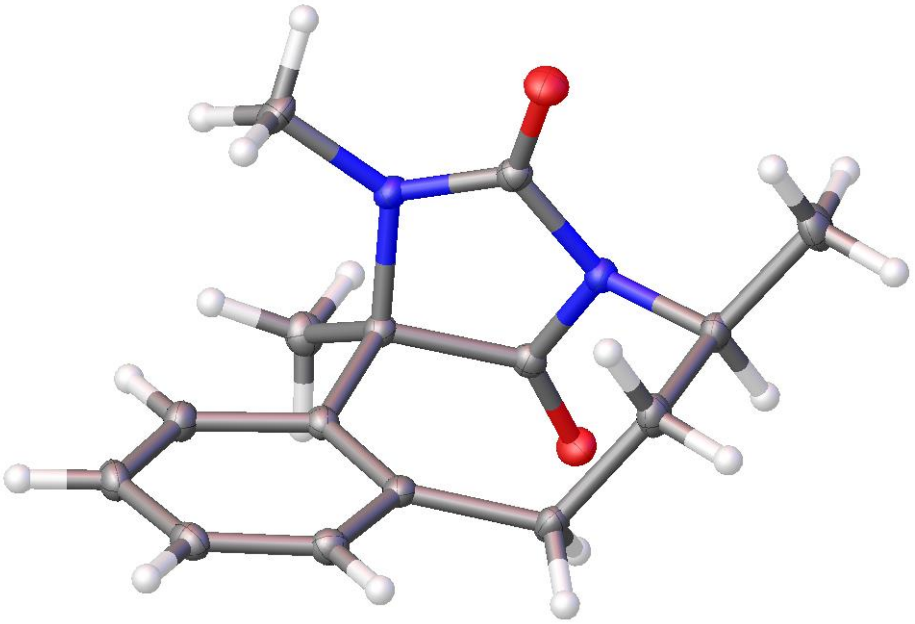

**Empirical formula**	$C_{15}H_{18}N_2O_2$
**Formula weight**	258.31
**Temperature/K**	100.01
**Crystal system**	Monoclinic
**Space group**	$P2_{1/n}$
**a/Å**	9.6859(2)
**b/Å**	8.2156(2)
**c/Å**	16.9618(3)
**α/°**	90
**β/°**	106.1200(10)
**γ/°**	90
**Volume/Å^3**	1296.67(5)
**Z**	4
**ρ_{calc}g/cm^3**	1.323
**μ/mm^{-1}**	0.089
**F(000)**	552.0
**Crystal size/mm^3**	0.543 x 0.24 x 0.163
**Radiation**	MoKα (λ = 0.71073)
**2Θ range for data collection**	4.398 to 60.116
**Index ranges**	-13 ≤ h ≤ 13, -7 ≤ k ≤ 11, -23 ≤ l ≤ 23
**Reflections collected**	26574
**Independent reflections**	3801 [R_{int} = 0.0423, R_{sigma} = 0.0276]
**Data/restraints/parameters**	3801/0/175
**Goodness-of-fit on F^2**	1.014
**Final R indexes [I>=2σ (I)]**	R_1 = 0.0396, wR_2 = 0.0968
**Final R indexes [all data]**	R_1 = 0.0530, wR_2 = 0.1047
**Largest diff. peak/hole/ eÅ^{-3}**	0.41/-0.21

5 References

[1] B. P. Corbet, J. V Matlock, J. Mas-Roselló, J. Clayden, *C. R. Chim.* **2017**, *20*, 634–642.

[2] M. F. T. Koehler, P. Bergeron, E. F. Choo, K. Lau, C. Ndubaku, D. Dudley, P. Gibbons, B. E. Sleebs, C. S. Rye, G. Nikolakopoulos, et al., *ACS Med. Chem. Lett.* **2014**, *5*, 662–667.

[3] J. Mas-Roselló, S. Hachisu, J. Clayden, *Angew. Chemie - Int. Ed.* **2017**, *56*, 10750–10754.

[4] J. L. Kenwright, W. R. J. D. Galloway, L. Wortmann, D. R. Spring, *Synth. Commun.* **2013**, *43*, 1508–1516.

[5] H. Ren, M. Zanger, J. R. McKee, *Synth. Commun.* **2006**, *36*, 355–363.

[6] R. K. Saunthwal, M. T. Cornall, R. Abrams, J. W. Ward, J. Clayden, *Chem. Sci.* **2019**, *10*, 3408–3412.

[7] W. Guerrab, R. Akrad, M. Ansar, J. Taoufik, J. T. Mague, Y. Ramli, *IUCrData* **2017**, *2*, DOI 10.1107/s2414314617015917.

[8] I. Nicolas, O. Jeannin, D. Pichon, M. Fourmigué, *CrystEngComm* **2016**, *18*, 9325–9333.

[9] L. Maini, D. Braga, F. Farinella, E. Melotto, M. Verzini, R. Brescello, I. Michieletto, I. Munari, *Cryst. Growth Des.* **2018**, *18*, 3774–3780.

[10] Bruker, *SAINT+ v8.38A Integration Engine, Data Reduction Software, Bruker Analytical X-Ray Instruments Inc.*, Madison, WI, USA, **2015**.

[11] Bruker, *SADABS 2014/5, Bruker AXS Area Detector Scaling and Absorption Correction, Bruker Analytical X-Ray Instruments Inc.*, Madison, Wisconsin, USA, **n.d.**

[12] G. M. Sheldrick, *Acta Crystallogr. Sect. A Found. Crystallogr.* **2008**, *64*, 112–122.

[13] L. Palatinus, G. Chapuis, *J. Appl. Crystallogr.* **2007**, *40*, 786–790.

[14] L. Palatinus, S. J. Prathapa, S. van Smaalen, *J. Appl. Crystallogr.* **2012**, *45*, 575–580.

[15] G. M. Shelrick, *Acta. Crystallogr. a-Foundtion Adv.* **2015**, *71*, 3–8.

[16] G. M. Shelrick, *Acta. Crystallogr. C* **2015**, *71*, 3–8.

[17] O. V. Dolomanov, L. J. Bourhis, R. J. Gildea, A. K. Howard, H. Puschmann, *J. Appl. Crystallogr.* **2009**, *42*, 339–341.
